# A proteomic profiling of laser-microdissected lung adenocarcinoma cells of early lepidic-types

**DOI:** 10.1186/s40169-015-0064-3

**Published:** 2015-07-03

**Authors:** Yasufumi Kato, Haruhiko Nakamura, Hiromasa Tojo, Masaharu Nomura, Toshitaka Nagao, Takeshi Kawamura, Tatsuhiko Kodama, Tatsuo Ohira, Norihiko Ikeda, Thomas Fehniger, György Marko-Varga, Toshihide Nishimura, Harubumi Kato

**Affiliations:** Department of Thoracic and Thyroid Surgery, Tokyo Medical University, Tokyo, Japan; Department of Chest Surgery, St. Mariana University School of Medicine, Kanagawa, Japan; Department of Biophysics and Biochemistry, Osaka University Graduate School of Medicine, Suita, Japan; Division of Diagnostic Pathology, Tokyo Medical University Hospital, Tokyo, Japan; Laboratory for Systems Biology and Medicine, Research Center for Advanced Science and Technology, The University of Tokyo, Tokyo, Japan; Center of Excellence in Biological and Medical Mass Spectrometry, Lund University, BMC, Lund, Sweden; Clinical Protein Science & Imaging, Biomedical Center, Department of Biomedical Engineering, Lund University, BMC, Lund, Sweden; Niizashiki Central General Hospital, Saitama, Japan

**Keywords:** Lung cancer, Adenocarcinoma, Lepidic type adenocarcinoma, Adenocarcinoma in situ, Minimally invasive adenocarcinoma, Comparative proteomics, Formalin-fixed and paraffin-embedded tissue sections, Laser microdissection, Mass spectrometry, Protein-protein interaction

## Abstract

**Background:**

In the new pathologic classification of lung adenocarcinoma proposed by IASLC/ATS/ERS in 2011, lepidic type adenocarcinomas are constituted by three subtypes; adenocarcinoma in situ (AIS), minimally invasive adenocarcinoma (MIA) and lepidic predominant invasive adenocarcinoma (LPIA). Although these subtypes are speculated to show sequential progression from preinvasive lesion to invasive lung cancer, changes of protein expressions during these processes have not been fully studied yet. This study aims to glimpse a proteomic view of the early lepidic type lung adenocarcinomas.

**Methods:**

A total of nine formalin-fixed and paraffin-embedded (FFPE) lepidic type lung adenocarcinoma tissues were selected from our archives, three tissues each in AIS, MIA and LPIA. The tumor and peripheral non-tumor cells in these FFPE tissues were collected with laser microdissection (LMD). Using liquid chromatography-tandem mass spectrometry (MS/MS), protein compositions were compared with respect to the peptide separation profiles among tumors collected from three types of tissues, AIS, MIA and LPIA. Proteins identified were semi-quantified by spectral counting-based or identification-based approach, and statistical evaluation was performed by pairwise G-tests.

**Results:**

A total of 840 proteins were identified. Spectral counting-based semi-quantitative comparisons of all identified proteins through AIS to LPIA have revealed that the protein expression profile of LPIA was significantly differentiated from other subtypes. 70 proteins including HPX, CTTN, CDH1, EGFR, MUC1 were found as LPIA-type marker candidates, 15 protein candidates for MIA-type marker included CRABP2, LMO7, and RNPEP, and 26 protein candidates for AIS-type marker included LTA4H and SOD2. The STRING gene set enrichment resulted from the protein-protein interaction (PPI) network analysis suggested that AIS was rather associated with pathways of focal adhesion, adherens junction, tight junction, that MIA had a strong association predominantly with pathways of proteoglycans in cancer and with PI3K-Akt. In contrast, LPIA was associated broadly with numerous tumor-progression pathways including ErbB, Ras, Rap1 and HIF-1 signalings.

**Conclusions:**

The proteomic profiles obtained in this study demonstrated the technical feasibility to elucidate protein candidates differentially expressed in FFPE tissues of LPIA. Our results may provide candidates of disease-oriented proteins which may be related to mechanisms of the early-stage progression of lung adenocarcinoma.

**Electronic supplementary material:**

The online version of this article (doi:10.1186/s40169-015-0064-3) contains supplementary material, which is available to authorized users.

## Background

Lung cancer is the leading cause of cancer-related mortality worldwide [1]. In Japan, annual deaths from lung cancer are increasing and currently approach about 70,000 [2], while in the United States with a recent decreasing trend in mortality, more than 160,000 succumb annually [3]. In an increasing trend worldwide, advances in chest high-resolution computed tomography (HRCT) scanning technology have enabled the localization of small adenocarcinoma nodules [4] at an earlier and potentially more curable stage of development than previously possible [5]. There are 90 million current and ex-smokers in the United States who are at increased risk of lung cancer. The published data from the National Lung Screening Trial (NLST) suggest that yearly screening with low-dose thoracic CT scan in heavy smokers can reduce lung cancer mortality by 20 % and all-cause mortality by 7-% [6].

In 2011, the new pathologic classification of lung adenocarcinoma was proposed by the International Association for the Study of Lung Cancer (IASLC), the American Thoracic Society (ATS) and the European Respiratory Society (ERS) [7]. In the new classification, the concept of adenocarcinoma in situ (AIS) and minimally invasive adenocarcinoma (MIA) were newly introduced and the term bronchioloalveolar carcinoma (BAC) was abolished. Additionally, invasive adenocarcinomas were categorized into 6 subtypes, lepidic, acinar, papillary, micropapillary, solid, and variants, according to the predominant histologic pattern. Both AIS and MIA were defined as tumors ≤ 3 cm in size. AIS is a preinvasive lesion showing pure lepidic growth without invasion. MIA is also lepidic predominant tumor but with ≤ 5 mm invasion. LPIA is an invasive adenocarcinoma showing former nonmucinous BAC pattern with > 5 mm invasion. These 3 lepidic type adenocarcinomas are speculated to show step-wise progression from AIS, MIA, to LPIA. After complete resection of AIS or MIA, usually 100 % of recurrence-free 5-year survival can be obtained [7], while some recurrent cases are found after resection of LPIA [8–10]. Since postoperative prognoses between the AIS plus MIA group and LPIA are different, differential protein expressions associated with invasiveness of cancer cells in each subtype should play important roles to determine local recurrences and survivals. However, precise proteomic analyses using individual cells in these early adenocarcinomas have not yet been performed. To the best of our knowledge, this is the first report performing proteomic analysis using micro-dissected early phase lung adenocarcinoma cells.

Recent advancements in shotgun sequencing and quantitative mass spectrometry for protein analyses could make proteomics amenable to clinical biomarker discovery [11, 12]. Laser microdissection (LMD) made it possible to collect target cells from a variety of formalin fixed paraffin embedded (FFPE) cancer tissues. This study attempts to capture a proteomic view of LPIA in comparison with other early stage lung adenocarcimomas by utilizing a label-free identification-based (or spectral counting-based) semi-quantitative shotgun proteomics approach following LMD [13–19].

## Results and discussion

### Group comparisons by *Rsc* and G-statistics

We used Abacus [20] to select high-scoring proteins using the thresholds of PeptideProphet probability > 0.99 and ProteinProphet probability > 0.9 as described in “MATERIALS and METHODS”, resulting in identifying a total of 840 proteins and obtaining their values of raw fold change in log2 (*Rsc*). For G-test (*p* < 0.05) [21], the raw *SpC*s of all patients in each group were pooled, thereby improving the performance of G-test and decreasing false positive rates significantly [15, 22]. Next, the values of *Rsc* that is a measure of fold changes for protein expression levels were calculated as described in “Materials and Methods” using the spectral counts of these proteins.

**Fig. 1:**
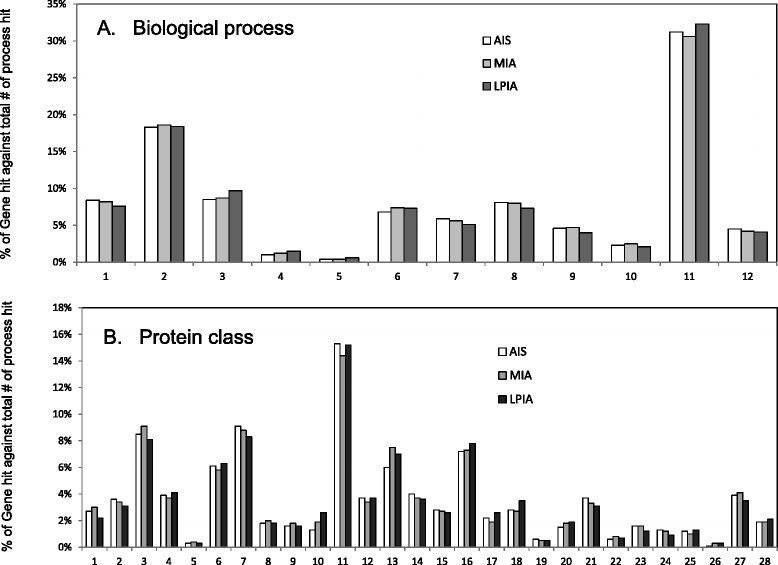
Gene ontology (GO) analysis for three cancer groups, AIS, MIA and LPIA, in which utilized were 544, 607, and 789 proteins, respectively, identified with *SpC* > 2. **a** Biological process: 1, cellular component organization or biogenesis (GO:0071840); 2, cellular process (GO:0009987); 3, localization (GO:0051179); 4, apoptotic process (GO:0006915); 5, reproduction (GO:0000003); 6, biological regulation (GO:0065007); 7, response to stimulus (GO:0050896); 8, developmental process (GO:0032502); 9, multicellular organismal process (GO:0032501); 10, biological adhesion (GO:0022610); 11, metabolic process (GO:0008152); 12, immune system process (GO:0002376). **b** Protein class: 1 extracellular matrix protein (PC00102); 2, protease (PC00190); 3, cytoskeletal protein (PC00085); 4, transporter (PC00227); 5, transmembrane receptor regulatory/adaptor protein (PC00226); 6, transferase (PC00220); 7, oxidoreductase (PC00176); 8, lyase (PC00144); 9, cell adhesion molecule (PC00069); 10, ligase (PC00142); 11, nucleic acid binding (PC00171); 12, signaling molecule (PC00207); 13, enzyme modulator (PC00095); 14, calcium-binding protein (PC00060); 15, defense/immunity protein (PC00090); 16, hydrolase (PC00121); 17, transfer/carrier protein (PC00219); 18, membrane traffic protein (PC00150); 19, phosphatase (PC00181); 20, transcription factor (PC00218); 21, chaperone (PC00072); 22, cell junction protein (PC00070); 23, surfactant (PC00212); 24, structural protein (PC00211); 25, kinase (PC00137); 26, storage protein (PC00210); 27, receptor (PC00197); 28, isomerase (PC00135)

The full lists of 840 proteins identified were provided as Additional file 1: Table S3. Proteins in LPIA, MIA and AIS identified under *SpC*_total_ > 2 for a protein were 789, 607, and 544, respectively, and were subjected to gene ontology (GO) analysis by using PANTHER Ver. 10.0 (http://www.pantherdb.org/). Results of (A) biological processes and (B) protein classes are shown in Fig. 1.

A marker candidate for the LPIA-type was chosen under the following criterions so that a protein had the pairwise *p*-value < 0.05 in G-test and *Rsc* < −1 against MIA and AI, [LPIA] (the retative abundance in spectral count) higher than [MIA] and [AIS], and total spectral counts throughout the desease states > 5. Table 1 summarizes 70 protein candidates thus obtained for LPIA from total 840 proteins identified, which are listed in increasing order of the *Rsc* (LPIA *vs.* MIA) values; the negatively larger the *Rsc* value of a given protein, the greater its expression level in LPIA compared with MIA and AIS. Those included beta-actin-like protein 2 (ACTBL2), tubulin alpha-1C chain (TUBA1C), band 7 protein family protein, HLA class I histocompatibility antigen, A-2 alpha chain (HLA-A), ARPC4-TTLL3 fusion protein, epiplakin (EPPK1), synaptogyrin-2 (SYNGR2), hemopexin (HPX), small nuclear ribonucleoprotein G-like protein (SNRPF), src substrate cortactin (CTTN), cadherin-1 (CDH1) (known as E-cadherin), epidermal growth factor receptor (EGFR), mucin-1 (MUC1), and promyelocytic leukemia protein (PML). The high expression of beta-actin-like protein 2 (ACTBL2) and tubulin alpha-1C chain (TUBA1C) might be related to active actin polymerization associated with invasiveness of LPIA.

**Table 1:** Seventy protein candidates characterizing LPIA listed in increasing order of the *Rsc* (LPIA vs. MIA) values; the negatively larger the Rsc value of a given protein, the greater its expression level in LPIA compared with MIA and AIS

					Spectral counts (SpCs)				Relative % thougtout stages			Fold change in log2 (R_SC_)			*p*-value in G-test		
No	Accession Number/Code	Gene ID	Description	Protein length (AA)	LPIA	MIA	AIS	Total	[LPIA]	[MIA]	[AIS]	LPIA vs MIA	LPIA vs AIS	LPIA vs pN	LPIA vs MIA	LPIA vs AIS	LPIA vs pN
1	HIP000323690	Band 7 protein family protein	Band7 protein family protein	2858	77	0	0	77	100.0	0.0	0.0	-5.816	-5.758	-4.482	2.33E-22	5.80E-22	1.67E-13
2	P01892	HLA-A	HLA class I histocompatibility antigen, A-2 alpha chain	365	34	0	0	34	100.0	0.0	0.0	-4.663	-4.605	-3.329	3.75E-10	5.59E-10	2.94E-06
3	Q15233	NONO	Non-POU domain-containing octamer-binding protein	471	32	0	6	38	84.2	0.0	15.8	-4.579	-1.984	-3.244	1.38E-09	9.10E-05	6.39E-06
4	P31948	STIP1	Stress-induced-phosphoprotein 1	543	27	0	5	32	84.4	0.0	15.6	-4.344	-1.963	-3.009	3.63E-08	3.43E-04	4.45E-05
5	P10253	GAA	Lysosomal alpha-glucosidase	952	26	0	0	26	100.0	0.0	0.0	-4.292	-4.233	-2.957	6.99E-08	9.48E-08	6.57E-05
6	Q07065	CKAP4	Cytoskeleton-associated protein 4	602	24	0	5	29	82.8	0.0	17.2	-4.181	-1.801	-2.847	2.58E-07	1.43E-03	1.43E-04
7	P17858	PFKL	6-phosphofructokinase, liver type	780	22	0	2	24	91.7	0.0	8.3	-4.062	-2.625	-2.727	9.55E-07	9.43E-05	3.11E-04
8	Q07960	ARHGAP1	Rho GTPase-activating protein 1	439	20	0	0	20	100.0	0.0	0.0	-3.932	-3.874	-2.598	3.53E-06	4.47E-06	6.77E-04
9	P06865	HEXA	Beta-hexosaminidase subunit alpha	529	19	0	0	19	100.0	0.0	0.0	-3.863	-3.804	-2.528	6.80E-06	8.49E-06	9.99E-04
10	P53007	SLC25A1	Tricarboxylate transport protein, mitochondrial	311	18	0	0	18	100.0	0.0	0.0	-3.790	-3.731	-2.455	1.31E-05	1.61E-05	1.47E-03
11	P36871	PGM1	Phosphoglucomutase-1	562	18	0	0	18	100.0	0.0	0.0	-3.790	-3.731	-2.455	1.31E-05	1.61E-05	1.47E-03
12	A0A0A6YYG9	ARPC4-TTLL3	ARPC4-TTLL3 fusion protein	625	18	0	0	18	100.0	0.0	0.0	-3.790	-3.731	-2.455	1.31E-05	1.61E-05	1.47E-03
13	Q96HE7	ERO1L	ERO1-like protein alpha	468	16	0	0	16	100.0	0.0	0.0	-3.631	-3.573	-2.296	4.84E-05	5.84E-05	3.21E-03
14	Q02218	OGDH	2-oxoglutarate dehydrogenase, mitochondrial	1023	16	0	1	17	94.1	0.0	5.9	-3.631	-2.725	-2.296	4.84E-05	5.45E-04	3.21E-03
15	P58107	EPPK1	Epiplakin	5090	15	0	0	15	100.0	0.0	0.0	-3.545	-3.487	-2.210	9.32E-05	1.11E-04	4.75E-03
16	P49588	AARS	Alanine--tRNA ligase, cytoplasmic	968	15	0	0	15	100.0	0.0	0.0	-3.545	-3.487	-2.210	9.32E-05	1.11E-04	4.75E-03
17	P16615	ATP2A2	Sarcoplasmic/endoplasmic reticulum calcium ATPase 2	997	15	0	0	15	100.0	0.0	0.0	-3.545	-3.487	-2.210	9.32E-05	1.11E-04	4.75E-03
18	P62873	GNB1	Guanine nucleotide-binding protein G(I)/G(S)/G(T) subunit beta-1	340	14	0	0	14	100.0	0.0	0.0	-3.453	-3.395	-2.119	1.80E-04	2.11E-04	7.01E-03
19	O43760	SYNGR2	Synaptogyrin-2	224	14	0	0	14	100.0	0.0	0.0	-3.453	-3.395	-2.119	1.80E-04	2.11E-04	7.01E-03
20	O60701	UGDH	UDP-glucose 6-dehydrogenase	494	12	0	0	12	100.0	0.0	0.0	-3.250	-3.192	-1.916	6.66E-04	7.66E-04	1.53E-02
21	Q5T2N8	ATAD3C	ATPase family AAA domain-containing protein 3C	411	11	0	0	11	100.0	0.0	0.0	-3.137	-3.079	-1.802	1.28E-03	1.46E-03	2.27E-02
22	P46782	RPS5	40S ribosomal protein S5	204	11	0	0	11	100.0	0.0	0.0	-3.137	-3.079	-1.802	1.28E-03	1.46E-03	2.27E-02
23	Q8NBJ7	SUMF2	Sulfatase-modifying factor 2	301	11	0	2	13	84.6	0.0	15.4	-3.137	-1.700	-1.802	1.28E-03	3.46E-02	2.27E-02
24	Q96AE4	FUBP1	Far upstream element-binding protein 1	644	10	0	0	10	100.0	0.0	0.0	-3.014	-2.956	-1.679	2.48E-03	2.78E-03	3.36E-02
25	P46783	RPS10	40S ribosomal protein S10	165	10	0	0	10	100.0	0.0	0.0	-3.014	-2.956	-1.679	2.48E-03	2.78E-03	3.36E-02
26	P17516	AKR1C4	Aldo-keto reductase family 1 member C4	323	10	0	0	10	100.0	0.0	0.0	-3.014	-2.956	-1.679	2.48E-03	2.78E-03	3.36E-02
27	P15531	NME1	Nucleoside diphosphate kinase A	152	10	0	0	10	100.0	0.0	0.0	-3.014	-2.956	-1.679	2.48E-03	2.78E-03	3.36E-02
28	P15428	HPGD	15-hydroxyprostaglandin dehydrogenase [NAD(+)]	266	10	0	0	10	100.0	0.0	0.0	-3.014	-2.956	-1.679	2.48E-03	2.78E-03	3.36E-02
29	O43776	NARS	Asparagine--tRNA ligase, cytoplasmic	548	10	0	0	10	100.0	0.0	0.0	-3.014	-2.956	-1.679	2.48E-03	2.78E-03	3.36E-02
30	P68036	UBE2L3	Ubiquitin-conjugating enzyme E2 L3	154	9	0	0	9	100.0	0.0	0.0	-2.880	-2.821	-1.545	4.78E-03	5.30E-03	4.97E-02
31	P54802	NAGLU	Alpha-N-acetylglucosaminidase	743	9	0	0	9	100.0	0.0	0.0	-2.880	-2.821	-1.545	4.78E-03	5.30E-03	4.97E-02
32	P11586	MTHFD1	C-1-tetrahydrofolate synthase, cytoplasmic	935	9	0	0	9	100.0	0.0	0.0	-2.880	-2.821	-1.545	4.78E-03	5.30E-03	4.97E-02
33	P02790	HPX	Hemopexin	462	9	0	0	9	100.0	0.0	0.0	-2.880	-2.821	-1.545	4.78E-03	5.30E-03	4.97E-02
34	A8MWD9	SNRPF	Small nuclear ribonucleoprotein G-like protein	76	9	0	0	9	100.0	0.0	0.0	-2.880	-2.821	-1.545	4.78E-03	5.30E-03	4.97E-02
35	Q9Y3U8	RPL36	60S ribosomal protein L36	105	8	0	0	8	100.0	0.0	0.0	-2.732	-2.673	-1.397	9.22E-03	1.01E-02	7.37E-02
36	Q14247	CTTN	Src substrate cortactin	634	8	0	0	8	100.0	0.0	0.0	-2.732	-2.673	-1.397	9.22E-03	1.01E-02	7.37E-02
37	P62491	RAB11A	Ras-related protein Rab-11A	216	8	0	0	8	100.0	0.0	0.0	-2.732	-2.673	-1.397	9.22E-03	1.01E-02	7.37E-02
38	P56192	MARS	Methionine--tRNA ligase, cytoplasmic	900	8	0	0	8	100.0	0.0	0.0	-2.732	-2.673	-1.397	9.22E-03	1.01E-02	7.37E-02
39	P12830	CDH1	Cadherin-1	882	8	0	0	8	100.0	0.0	0.0	-2.732	-2.673	-1.397	9.22E-03	1.01E-02	7.37E-02
40	P05166	PCCB	Propionyl-CoA carboxylase beta chain, mitochondrial	559	8	0	0	8	100.0	0.0	0.0	-2.732	-2.673	-1.397	9.22E-03	1.01E-02	7.37E-02
41	O75347	TBCA	Tubulin-specific chaperone A	108	8	0	0	8	100.0	0.0	0.0	-2.732	-2.673	-1.397	9.22E-03	1.01E-02	7.37E-02
42	O43684	BUB3	Mitotic checkpoint protein BUB3	328	8	0	0	8	100.0	0.0	0.0	-2.732	-2.673	-1.397	9.22E-03	1.01E-02	7.37E-02
43	Q9UM22	EPDR1	Mammalian ependymin-related protein 1	224	7	0	0	7	100.0	0.0	0.0	-2.567	-2.508	-1.232	1.78E-02	1.93E-02	1.09E-01
44	Q14376	GALE	UDP-glucose 4-epimerase	348	7	0	0	7	100.0	0.0	0.0	-2.567	-2.508	-1.232	1.78E-02	1.93E-02	1.09E-01
45	P48637	GSS	Glutathione synthetase	474	7	0	0	7	100.0	0.0	0.0	-2.567	-2.508	-1.232	1.78E-02	1.93E-02	1.09E-01
46	P47897	QARS	Glutamine--tRNA ligase	775	7	0	0	7	100.0	0.0	0.0	-2.567	-2.508	-1.232	1.78E-02	1.93E-02	1.09E-01
47	P15941	MUC1	Mucin-1	475	7	0	0	7	100.0	0.0	0.0	-2.567	-2.508	-1.232	1.78E-02	1.93E-02	1.09E-01
48	O60763	USO1	General vesicular transport factor p115	962	7	0	0	7	100.0	0.0	0.0	-2.567	-2.508	-1.232	1.78E-02	1.93E-02	1.09E-01
49	P46977	STT3A	Dolichyl-diphosphooligosaccharide--protein glycosyltransferase subunit STT3A	705	12	1	0	13	92.3	7.7	0.0	-2.402	-3.192	-1.916	4.91E-03	7.66E-04	1.53E-02
50	O43488	AKR7A2	Aflatoxin B1 aldehyde reductase member 2	358	12	1	1	14	85.7	7.1	7.1	-2.402	-2.344	-1.916	4.91E-03	5.59E-03	1.53E-02
51	Q9Y3I0	C22orf28	tRNA-splicing ligase RtcB homolog	505	6	0	0	6	100.0	0.0	0.0	-2.380	-2.322	-1.045	3.45E-02	3.70E-02	1.63E-01
52	Q9NRV9	HEBP1	Heme-binding protein 1	189	6	0	0	6	100.0	0.0	0.0	-2.380	-2.322	-1.045	3.45E-02	3.70E-02	1.63E-01
53	Q9BPW8	NIPSNAP1	Protein NipSnap homolog 1	284	6	0	0	6	100.0	0.0	0.0	-2.380	-2.322	-1.045	3.45E-02	3.70E-02	1.63E-01
54	P49419	ALDH7A1	Alpha-aminoadipic semialdehyde dehydrogenase	539	6	0	0	6	100.0	0.0	0.0	-2.380	-2.322	-1.045	3.45E-02	3.70E-02	1.63E-01
55	P29590	PML	Protein PML	633	6	0	0	6	100.0	0.0	0.0	-2.380	-2.322	-1.045	3.45E-02	3.70E-02	1.63E-01
56	P14868	DARS	Aspartate--tRNA ligase, cytoplasmic	501	6	0	0	6	100.0	0.0	0.0	-2.380	-2.322	-1.045	3.45E-02	3.70E-02	1.63E-01
57	P00533	EGFR	Epidermal growth factor receptor	705	6	0	0	6	100.0	0.0	0.0	-2.380	-2.322	-1.045	3.45E-02	3.70E-02	1.63E-01
58	Q02252	ALDH6A1	Methylmalonate-semialdehyde dehydrogenase [acylating], mitochondrial	535	29	4	9	42	69.0	9.5	21.4	-2.372	-1.348	-3.108	2.84E-05	4.68E-03	2.05E-05
59	Q99829	CPNE1	Copine-1	537	26	4	7	37	70.3	10.8	18.9	-2.221	-1.510	-2.957	1.38E-04	3.51E-03	6.57E-05
60	Q15363	TMED2	Transmembrane emp24 domain-containing protein 2	201	10	1	0	11	90.9	9.1	0.0	-2.166	-2.956	-1.679	1.57E-02	2.78E-03	3.36E-02
61	P35221	CTNNA1	Catenin alpha-1	906	28	5	10	43	65.1	11.6	23.3	-2.072	-1.165	-3.059	1.61E-04	1.31E-02	3.02E-05
62	O75340	PDCD6	Programmed cell death protein 6	191	30	6	3	39	76.9	15.4	7.7	-1.953	-2.665	-3.155	1.78E-04	5.33E-06	1.39E-05
63	O00764	PDXK	Pyridoxal kinase	312	11	2	2	15	73.3	13.3	13.3	-1.759	-1.700	-1.802	3.11E-02	3.46E-02	2.27E-02
64	P13667	PDIA4	Protein disulfide-isomerase A4	645	70	18	26	114	61.4	15.8	22.8	-1.735	-1.174	-4.346	1.64E-07	7.79E-05	2.52E-12
65	Q9NTX5	ECHDC1	Ethylmalonyl-CoA decarboxylase	307	28	7	1	36	77.8	19.4	2.8	-1.671	-3.487	-3.059	1.17E-03	4.05E-07	3.02E-05
66	Q7Z4W1	DCXR	L-xylulose reductase	244	23	7	0	30	76.7	23.3	0.0	-1.400	-4.065	-2.788	9.91E-03	6.51E-07	2.11E-04
67	Q9Y2Q3	GSTK1	Glutathione S-transferase kappa 1	226	14	4	3	21	66.7	19.0	14.3	-1.383	-1.629	-2.119	4.58E-02	2.20E-02	7.01E-03
68	P49748	ACADVL	Very long-chain specific acyl-CoA dehydrogenase, mitochondrial	655	30	10	12	52	57.7	19.2	23.1	-1.319	-1.024	-3.155	4.98E-03	2.05E-02	1.39E-05
69	P42224	STAT1	Signal transducer and activator of transcription 1- alpha/beta	750	16	5	0	21	76.2	23.8	0.0	-1.309	-3.573	-2.296	4.13E-02	5.84E-05	3.21E-03
70	O95994	AGR2	Anterior gradient protein 2 homolog	175	58	23	14	95	61.1	24.2	14.7	-1.135	-1.746	-4.079	5.21E-04	1.57E-06	2.65E-10

Src substrate cortactin (CTTN), epidermal growth factor receptor (EGFR) and mucin-1 (MUC1) expressed in LPIA might reflect its invasiveness with aggressive proliferation. Invasive carcinoma cells degrade and invade through the extracellular matrix (ECM) by invadopodia, where an EGFR–Src–Arg–cortactin pathway is considered to mediate functional maturation of invadopodia [23–25]. Overexpression of cortactin protein (CTTN) has been currently considered to be an important biomarker for invasive cancers because of its frequent link to various invasive cancers, including melanoma, colorectal, and glioblastoma [25].

Proteins expressed increasingly along the disease stages from AIS to LPIA, which might be considered to be disease progression-related, included were alpha-enolase (ENO1), plectin (PLEC), major vault protein (MVP), heterogeneous nuclear ribonucleoprotein M (HNRNPM), 14-3-3 protein sigma (SFN), lysophosphatidylcholine acyltransferase 1 (LPCAT1), anterior gradient protein 2 homolog (AGR2), phospholipase D3 (PLD3), hypoxia up-regulated protein 1 (HYOU1), fatty acid synthase (FASN), programmed cell death protein 6 (PDCD6), and ethylmalonyl-CoA decarboxylase (ECHDC1). Among proteins expressed characteristically in the AIS and MIA disease stages, leukotriene A-4 hydrolase (LTA4H) in AIS and cellular retinoic acid-binding protein 2 (CRABP2) in MIA, respectively, were representative. Proteins significant to AIS and MIA are provided in Additional file 1: Table S1 and S2. Enhanced AGR2 expression has been observed in most human adenocarcinomas, including pancreas, lung, ovary, breast and prostate, frequently suggesting its association with tumor progression and metastasis [13, 14, 26–30]. The HYOU1 protein (hypoxia up-regulated 1, alternatively known as Orp150), belonging to the heat shock protein 70 family, demonstrated its increased expression in prostate, bladder and invasive breast cancer, is suggested to be associated with tumor invasiveness [31].

### Protein-protein network analysis of expressed proteins

**Fig. 2:**
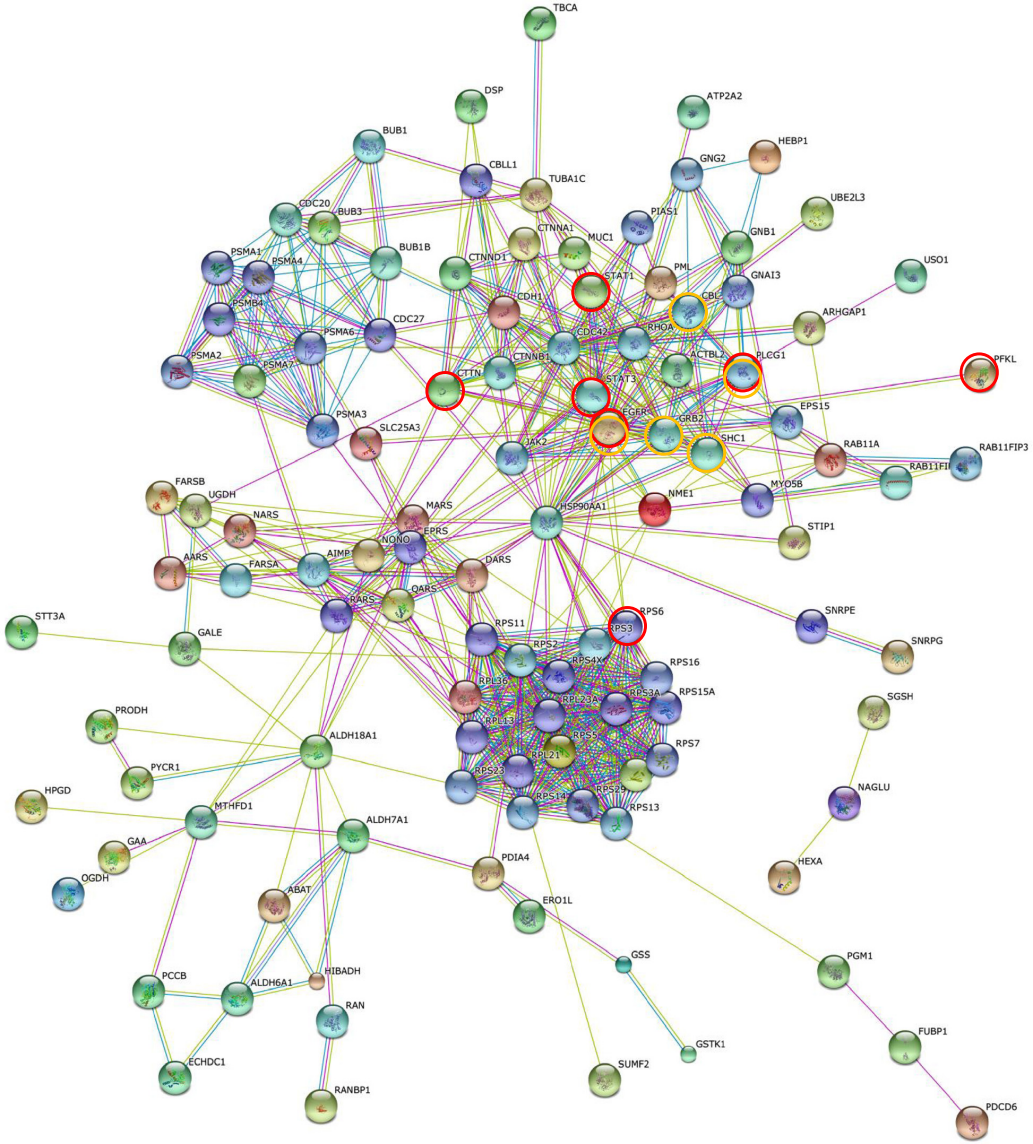
The high-resolution evidence-view of STRING PPI networks obtained on LPIA by using 70 proteins significantly expressed (listed in Table 1), which were generated using default setting in network depth of 50 interactions under medium confidence (0.4) and standard criteria for linkage only with experiments, databases, and textmining

**Fig. 3:**
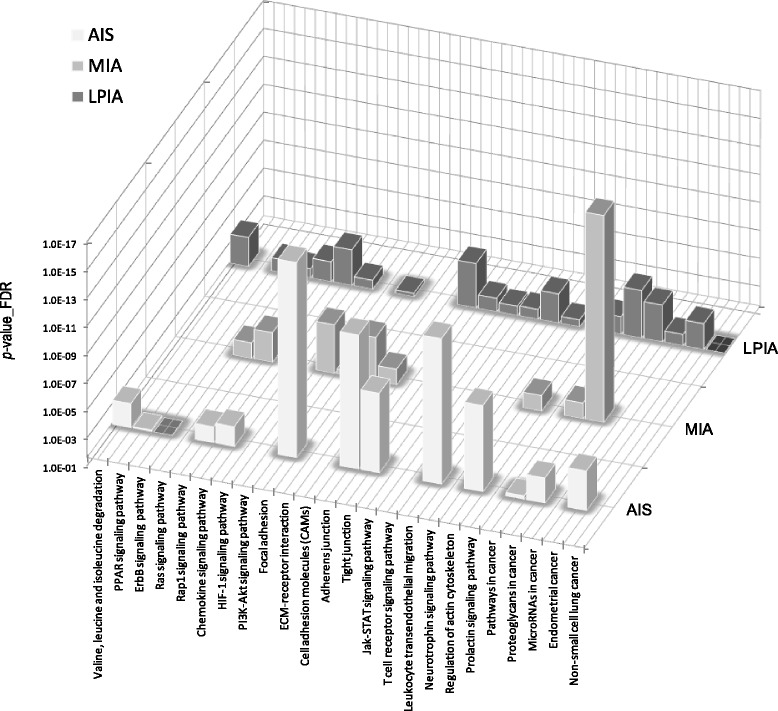
Cancer-related pathways associated with AIS, MIA and LPIA, which were obtained by the STRING enrichment analysis under the statistical significance of *p* <0.05

Network analysis of significant proteins is also helpful to understand how they interplay with other key proteins and pathways. In this study the network analysis of protein-protein interaction was performed by utilizing the STRING database version 10 [32, 33]. Therein only experiments, databases and text mining were utilized to avoid less confident predicted interactions. The STRING PPI networks were obtained by applying 70 proteins expressed significantly to LPIA (given in Table 1) and shown in Fig. 2 (also given as Additional file 2: Figure S1). The STRING PPI networks obtained for AIS and MIA are provided as Additional file 3: Figure S2 and Additional file 4: Figure S3. Figure 3 illustrates results of the STRING gene set enrichments (GSEs) for LPIA, MIA, and AIS obtained against cancer related KEGG pathways, which were elucidated with their significance rank *p* < 0.05 after correction by false discovery rate (FDR). All results are provided in Additional file 1: Table S4. Enrichments on AIS indicated the strong association with pathways of focal adhesion (*p* = 2.69 × 10^−16^), adherens junction (*p* = 6.45 × 10^−12^) and leukocyte transendothelial migration (*p* = 9.79 × 10^−13^). MIA was found to be associated with PI3K-Akt signaling (*p* = 8.25 × 10^−6^) and predominantly with proteoglycans in cancer (*p* = 3.99 × 10^−17^). In contrast, LPIA was associated broadly with numerous cancer-related pathways which included proteoglycans in cancer (*p* = 5.94 × 10^−5^), ErbB signaling (*p* = 2.99 × 10^−3^), Ras signaling (*p* = 5.77 × 10^−3^), Rap1 signaling (*p* = 9.72 × 10^−4^), chemokine signaling (*p* = 7.23 × 10^−5^), and HIF-1 signaling (*p* = 5.77 × 10^−3^).

Proteoglycans are known to be important molecular effectors of cell surface and pericellular microenvironments and to have multiple functions in cancer and angiogenesis by interacting with both ligands and receptors that regulate neoplastic growth and neovascularization [34]. Molecules participating in the proteoglycan-related cancer pathway were denoted by red circles in Additional file 3: Figure S2. The ErbB signaling pathway is associated with many cancer pathways. The ErbB family belong epidermal growth factor receptors which play an important role in tumor growth. Over-expression of EGFR occurs around 60 % of non-small cell lung cancer (NSCLC), in which adenocarcinoma has the higher frequency [35]. Hypoxia-inducible factors (HIFs) regulate the transcription of genes that mediate the response to hypoxia (reduced O_2_ availability) [36]. It is considered that diverse products of HIF-1 action such as induction of the Met protein, hepatocyte growth factor (HGF), followed by Met receptor activation may result in the poor prognosis attached to hypoxic tumors, which indeed turn out to be more aggressive that their well-oxygenerated counterparts. Molecules participating in the ErbB and HIF-1 signaling pathways were denoted by orange and red circles, respectively, in Fig. 2. Numerous clinical data demonstrated that increased levels of HIF-1 proteins consequenced a poor prognosis and increased patient mortality in many different human cancers including NSCLC [37].

## Conclusion

Former localized BAC (≦2 cm) lesions have been histologically classified into types A, B and C by Noguchi et al. based on finding of local cancer progression [38]. These lesions, now identified as AIS, MIA or LPIA usually show focal ground-glass opacity (GGO) on chest HRCT (high resolution computed tomography). Generally, AIS shows pure GGO, and representative MIA and LPIA lesions show GGO with some intratumoral areas of collapsed shadow suggesting invasion. There are multiple studies [39, 40] describing that limited lung resection including wedge resection or segmentectomy can cure early adenocarcinomas showing pure GGO. From histologic and radiologic points of view, it is hypothesized that preinvasive AIS progresses to the invasive lesions MIA and LPIA sequentially. Postsurgical 5-year recurrence-free survival rates for AIS and MIA are 100 %, while these for LPIA ranged 71.9 to 93.8 % [8–10].

The molecular biological background predisposing the worse prognosis of LPIA compared with AIS and MIA may be in part due to the forms of altered protein expressions found in our present study. Proteins appearing in the step from AIS to MIA are probably important at the initial step of microinvasion. As LPIA prepares characteristics of matured lung cancer, it is reasonable that LPIA expresses a variety of proteins associated with cancer invasion. We believe that some of these proteins are candidates for molecular target therapy to suppress local invasion or distant metastases.

In the new adenocarcinoma subtyping, prognoses of solid or micropapillary predominant invasive adenocarcinomas were reported to be apparently worse than these of other subtypes including lepidic type adenocarcinomas [10, 41, 42]. We imagine that in the future comparative proteomic analyses such as that presented here will contribute to elucidate protein expressions determining malignant grade of various lung adenocarcinoma subtypes, which will further provide important knowledge to understand the carcinogenetic process and tumor lineages of lung adenocarcinomas for the benefit of patients with more efficient diagnosis and treatment of these tumors.

## Method

### Ethics approval

The study protocol conformed to the principles of the Declaration of Helsinki. All patients were provided with informed consent and the study protocol was approved by Tokyo Medical University Hospital institutional ethics committee.

### FFPE tissues and sample preparation

Surgically removed lung tissues were fixed with a buffered formalin solution containing 10 – 15-% methanol, and embedded by a conventional method. Archived paraffin blocks of formalin-fixed adenocarcinoma tissues obtained from cases of AIS (*n* = 3), MIA (*n* = 3), and LPIA (*n* = 3), which were retrieved with the approval from Ethical Committee of Tokyo Medical University Hospital (Acception No. 1964). Patients’ characteristics are listed in Table 2. Paraffin blocks were cut into 4-μm sections for diagnosis and 10-μm sections for proteomics. The 10-μm sections were stained with only haematoxylin. Three pathologists (M.N., Y.K.) independently confirmed adenocarcinoma subtypes using the 4-μm sections stained with haematoxylin-eosin (HE).

**Table 2:** Patients’ characteristics

Patient number	Age	Sex	CEA (ng/ml)	Location	Tumor size on CT (mm)	Histological type		TNM classification	EGFR mutation
2	55	M	3.9	Rt.S1	11	pseudo-Normal^a^	AIS	T1aN0M0	Unkown
4	59	W	8.1	Lt.S1 + 2	15	pseudo-Normal^a^	LPIA	T1bN0M0	Unkown
9	55	W	5.3	Rt.S4	37	pseudo-Normal^a^	LPIA	T1bN0M0	Unkown
1	63	W	1.9	Rt.S2	15	Adenocarcinoma	AIS	T1aN0M0	Unkown
2	55	M	3.9	Rt.S1	11	Adenocarcinoma	AIS	T1aN0M0	Unkown
3	56	W	2	Lt.S8	10	Adenocarcinoma	AIS	T1aN0M0	Unkown
4	59	W	8.1	Lt.S3	28	Adenocarcinoma	MIA	T1bN0M0	Unkown
5	73	W	5.4	Rt.S5	25	Adenocarcinoma	MIA	T1bN0M0	Unkown
6	74	W	1.5	Lt.S3	25	Adenocarcinoma	MIA	T1bN0M0	L858R
7	78	W	1.5	Rt.S3	37	Adenocarcinoma	LPIA	T2aN0M0	L858R
8	72	W	0.6	Rt.S1	51	Adenocarcinoma	LPIA	T2aN0M0	Unkown
9	55	W	5.3	Rt.S4	37	Adenocarcinoma	LPIA	T1bN0M0	Unkown

^a^lesions judged pathologically as non-cancerous regions adjacent to tumors

### Laser capture and protein solubilization

**Fig. 4:**
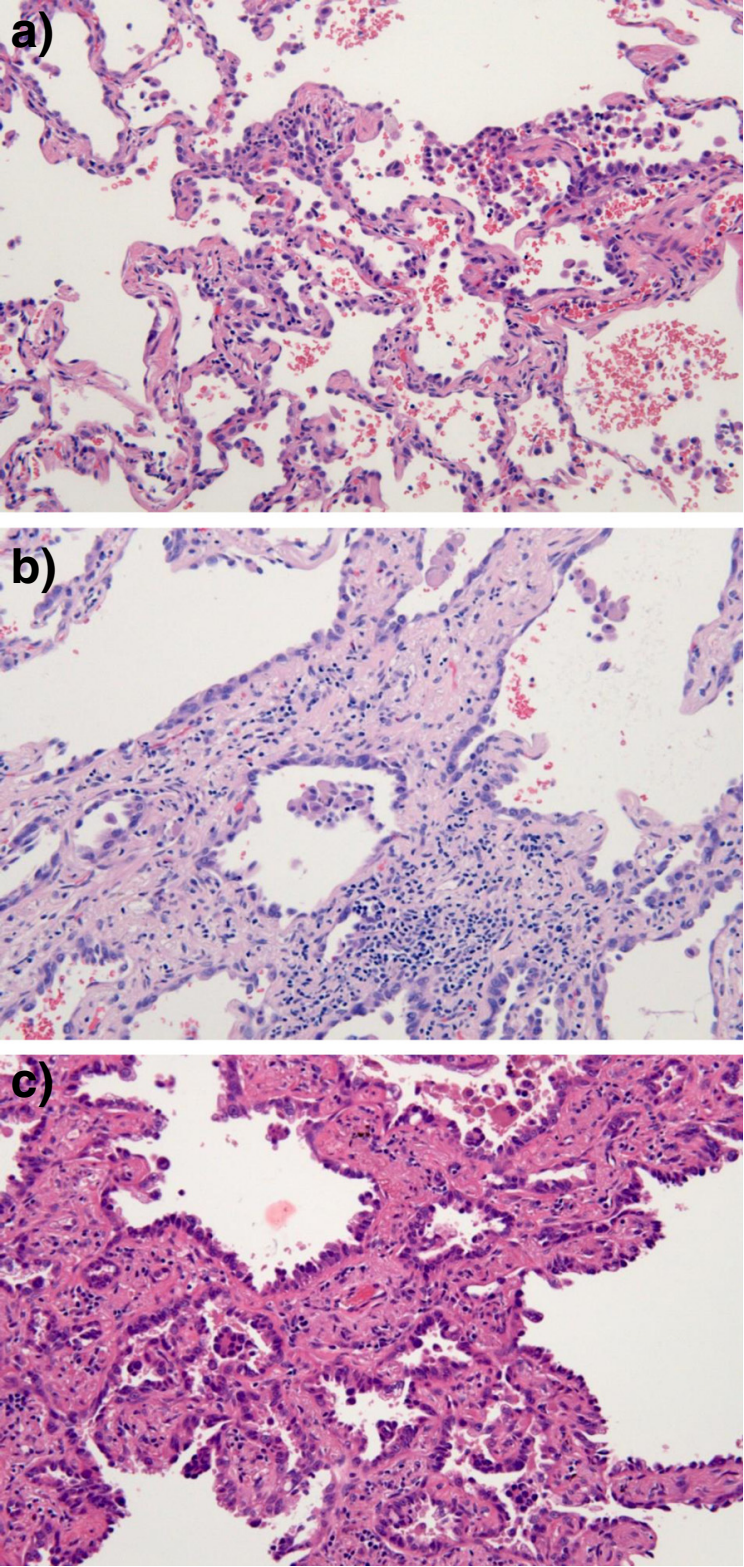
Representative HE-stained images of **a**) adenocarcinoma in situ (AIS), **b**) minimally invasive adenocarcinoma (MIA), and **c**) lepidic predominant invasive adenocarcinoma (LPIA). In AIS, no foci of invasion or scarring could not be seen, and atypical pneumocytes were proliferating along the slightly thickened alveolar wall. In MIA, showing a small area of invasion (<0.5 cm), tumor cells grew mostly in lepidic pattern along the surface of alveolar walls. In LPIA, showing a larger area of invasion (≧0.5 cm), type II pneumocytes and Clara cells were proliferating along the surface of thickened alveolar walls. Alveolar epitheliums are substituted in tumor cells, together with examples of targeted lesions before and after laser-microdissections (LMD)

Cancerous lesions were identified on serial tissue sections stained with hematoxylin and eosin (HE). For proteomic analysis, a 10-μm thick section prepared from the same tissue block was attached onto DIRECTOR™ slides (OncoPlexDx, Rockville, MD, USA), de-paraffinized twice with xylene for 5-min, rehydrated with graded ethanol solutions and distilled water, and stained by hematoxylin. Those slides were air-dried and subjected to laser microdissection with a Leica LMD6000 (Leica Micro-systems GmbH, Ernst-Leitz-Strasse, Wetzlar, Germany). At least 30,000 cells (8.17 ± 0.03 mm^2^) per a tissue were collected directly into a 1.5-mL low-binding plastic tube. From individual three types tissues non-cancerous lesions far from tumors were also collect the same numbers of cells as the pseudo-normal (pN) group (*n* = 3). Representative HE-stained images of adenocarcinoma tissues of AIS, MIA and LPIA were shown in Fig. 4 together with examples of targeted lesions before and after laser-microdissections (LMD). Proteins were extracted and digested with trypsin using Liquid Tissue™ MS Protein Prep kits (OncoPlexDx, Rockville, MD, USA) according to the manufacturer’s protocol [43].

### Exploratory liquid chromatography-tandem mass spectrometry

The digested samples were analyzed in triplicate and in random order by liquid chromatography (LC)-tandem mass spectrometry (MS/MS) using reverse-phase liquid chromatography (RP-LC) interfaced with a LTQ-Orbitrap XL hybrid mass spectrometer (Thermo Fisher Scientific, Bremen, Germany) via a closed nano-electrospray device (ADVANCE Captive Spray Source; AMR Inc. Japan). The RP-LC system consisted of Paradigm MS4 (Michrom Bioresources, USA), a peptide Cap-Trap cartridge (0.3 × 5.0 mm) and a capillary separation column (an L-column Micro of 0.1 × 150 mm packed with reverse phase L-C18 gels of 3 μm in diameter and 12-nm pore size, (CERI, Tokyo, Japan)). An autosampler (HTC-PAL, CTC Analytics, Switzerland) loaded an aliquot of samples onto the trap, which then was washed with solvent A (98 % distilled water with 2 % acetonitrile and 0.1 % formic acid) for concentrating peptides on the trap and desalting. Subsequently, the trap was connected in series to the separation column, and the whole columns were developed for 100 min with a linear acetonitrile concentration gradient made from 5 to 35 % solvent B (10 % distilled water and 90 % acetonitrile containing 0.1 % formic acid) at the flow-rate of 300 nL/min.

An LTQ was operated in the data-dependent MS/MS mode to automatically acquire up to three successive MS/MS scans in the centroid mode. The three most intense precursor ions for these MS/MS scans could be selected from a high-resolution MS spectrum (a survey scan) that an Orbitrap previously acquired during a predefined short time window in the profile mode at the resolution of 30,000 and the lock mass of m/z 536.1654 in the m/z range of 350 to 1500. The sets of acquired high-resolution MS and MS/MS spectra for peptides were converted to single data files and they were merged into Mascot generic format files for database searching.

### Protein identification

To extract protein candidates characterizing lepidic type adenocarcinoma from the shotgun proteomic datasets experimentally acquired, consisting of 36 runs (triplicate runs of 12 samples from 4 groups, AIS, MIA, LPIA, and pseudo-normal, N), we have utilized a label-free spectral counting approach for proteomic data analysis on protein identification and semi-quantitative comparison.

Tha mass spectral raw data were analyzed using the one-path method of X! Tandem wth a *k*-score plugin in Trans-Proteomic pipeline (TPP) [44, 45] against the combined protein fasta file from Human-Invitational database (H-InvDB) [46], RefSeq, and UniProtKB/Swiss-Prot appended with reversed decoy sequences. Peptide mass tolerance was 10 ppm, fragment mass tolerance 0.5 Da, and up to two missed cleavages and non-tryptic cleavage at one end of a peptide were allowed. Methionine oxidation is considered as variable modification. The output files from the search engine were converted to the pepXML files and subjected to peptide-spectrum match (PSM) posterior probability calculation with PeptideProphet [47] and then to ProteinProphet for identification at the protein level in Trans-proteomic pipeline (TPP) [44, 45].

### Semi-quantitative comparison

For calculating spectral counts (*SpC*s) at the protein level, triplicate X!Tandem [48] results for each patient were simultaneously analyzed with PeptideProphet and the single output file was subjected to ProteinProphet [49]. In addition, all PeptideProphet results were simultaneously analyzed with ProteinProphet. Then, all PeptideProphet and ProtinProphet results were used for extracting significant proteins and computing *SpC*s with Abacus [20] using the following thresholds: maxiniProb threshold = 0.99 for the minimum PeptideProphet score, and Combined File Prob threshold = 0.9 for the minimum ProteinProphet score in the combined file.

Fold changes of expressed proteins in the base 2 logarithmic scale (*R*_*SC*_) were calculated using spectral counting as described [15]. Proteins expressed significantly between two groups were chosen so that their *R*_*SC*_ satisfy >1 or < −1, which correspond to their fold changes >2 or <0.5, and *p*-value < 0.05 in G-test [19]. Although G-test does not require replicates, spectral counts for each protein from triplicates were pooled and used for G-statistic calculation using a two-way contingency table arranged in two rows for a target protein and any other proteins, and two columns for cancer groups on an Excel macro. The Yates correction for continuity was applied to the 2 × 2 tables. The correction could enable us to handle the data containing small spectral counts including zero.

### Network analysis of protein-protein interactions

Network analysis of protein-protein interactions was carried out by using the Search Tool for the Retrieval of Interacting Genes/Proteins (STRING) database version 10 [32, 33], in which nodes are proteins and edges are the predicted functional associations based on primary databases comprising of KEGG and GO, and primary literature. STRING can predict these interactions based on neighbourhood, gene fusion products, homology and similarity of coexpression patterning, experiments, databases and textmining. Network interaction scores for each node are expressed as a joint probability derived from curated databases of experimental information, textmining and computationally predicted by genetic proximity [32]. In this study, STRING networks were calculated under the criteria for linkage only with experiments, databases, and textmining with the default settings - medium confidence score: 0.400, network depth: 0 and up to 50 interactions.

## Additional files

Additional file 1: Table S1. Proteins characteristic to AIS, which were under *p* < 0.05 (AIS vs LPIA), *Rsc* > 1 (LPIA vs. AIS), and [AIS] greater than [LPIA] and [MIA]. **Table S2.** Proteins characteristic to MIA, which were under *p* < 0.05 (MIA vs LPIA), *Rsc* > 1 (LPIA vs. MIA), and [MIA] greater than [LPIA] and [AIS]. **Table S3.** The list of total 840 proteins identified. **Table S4.** The STRING network enrichment results on KEGG pathways. Additional file 2: Figure S1. The high-resolution evidence-view of STRING PPI networks obtained on LPIA by using 70 proteins significantly expressed (listed in Table 1), which were generated using default setting in network depth of 50 interactions under medium confidence (0.4) and standard criteria for linkage only with experiments, databases, and text mining. Additional file 3: Figure S2. The high-resolution evidence-view of STRING PPI networks obtained on AIS by using 26 proteins significantly expressed (listed in Additional file 1: Table S1), which were generated using default setting in network depth of 50 interactions under medium confidence (0.4) and standard criteria for linkage only with experiments, databases, and text mining. Additional file 4: Figure S3. The high-resolution evidence-view of STRING PPI networks obtained on MIA by using 15 proteins significantly expressed (listed in Additional file 1: Table S2), which were generated using default setting in network depth of 50 interactions under medium confidence (0.4) and standard criteria for linkage only with experiments, databases, and text mining.

## Abbreviations

BAC: Bronchioloaveolar carcinoma
AIS: Adenocarcinoma in situ
MIA: Minimally invasive adenocarcinoma
LPIA: Lepidic predominant invasive adenocarcinoma
NSCLC: Non-small cell lung carcinoma
LC: Liquid chromatography
MS: Mass spectrometry
FFPE: Formalin-fixed paraffin embedded
LMD: Laser microdissection
HPLC: High pressure liquid chromatography
MS/MS: Tandem mass spectrometry
SpC: Spectral count
STRING: Search tool for the retrieval of interacting genes/proteins database
KEGG: Kyoto encyclopedia of genes and genomes
GO: Gene ontology
GSE: Gene set enrichment
