# Supplementary material for: A proteomic profiling of laser-microdissected lung adenocarcinoma cells of early lepidic-types
Source: Clin Transl Med. 2015 Jul 3;4:24. doi: 10.1186/s40169-015-0064-3 (PMC4501340; doi:10.1186/s40169-015-0064-3)
Supplement: Additional file 1: Table S1. — Proteins characteristic to AIS, which were under p < 0.05 (AIS vs LPIA), Rsc > 1 (LPIA vs. AIS), and [AIS] greater than [LPIA] and [MIA]. Table S2. Proteins characteristic to MIA, which were under p < 0.05 (MIA vs LPIA), Rsc > 1 (LPIA vs. MIA), and [MIA] greater than [LPIA] and [AIS]. Table S3. The list of total 840 proteins identified. Table S4. The STRING network enrichment results on KEGG pathways. [file 40169_2015_64_MOESM1_ESM.doc]

**SUPPLEMENTAL MATERIALS**

Table S1. Proteins characteristic to AIS, which were under *p* < 0.05 (AIS vs LPIA), *Rsc* > 1 (LPIA vs. AIS), and [AIS] greater than [LPIA] and [MIA].

|  |  |  |  |  |  | Spectral Counts *(SpCs*) | | | |  | Relative % thougtout Stages | | | |  | Fold change in log2 (*R_SC_*) | | |  | *p*-value in G-test | | |
| --- | --- | --- | --- | --- | --- | --- | --- | --- | --- | --- | --- | --- | --- | --- | --- | --- | --- | --- | --- | --- | --- | --- |
| No | Accession Number/Code | Gene ID | Description | Protein length (AA) |  | LPIA | MIA | AIIS | Total |  | [LPIA] | [MIA] | [AIS] | [pN] |  | LPIA vs MIA | LPIA vs AIS | LPIA vs pN |  | LPIA vs MIA | LPIA vs AIS | LPIA vs pN |
|  |  |  |  |  |  |  |  |  |  |  |  |  |  |  |  |  |  |  |  |  |  |  |
| 1 | P21333 | FLNA | Filamin-A | 2647 |  | 215 | 369 | 429 | 102 |  | 19.3 | 33.1 | 38.5 | 9.1 |  | 0.9441 | 1.2244 | 0.4290 |  | 1.186E-13 | 3.963E-23 | 9.816E-01 |
| 2 | P35555 | FBN1 | Fibrillin-1 | 2871 |  | 39 | 91 | 133 | 34 |  | 13.1 | 30.6 | 44.8 | 11.4 |  | 1.3566 | 1.9595 | 1.3032 |  | 5.402E-07 | 1.311E-15 | 1.396E-02 |
| 3 | P35749 | MYH11 | Myosin-11 | 1945 |  | 103 | 180 | 229 | 0 |  | 20.1 | 35.2 | 44.7 | 0.0 |  | 0.9602 | 1.3677 | -4.8971 |  | 1.435E-07 | 1.851E-15 | 6.911E-18 |
| 4 | P04264 | KRT1 | Keratin, type II cytoskeletal 1 | 644 |  | 111 | 202 | 213 | 194 |  | 15.4 | 28.1 | 29.6 | 26.9 |  | 1.0198 | 1.1555 | 2.3163 |  | 4.167E-09 | 2.924E-11 | 1.951E-29 |
| 5 | P18206 | VCL | Vinculin | 1066 |  | 64 | 105 | 149 | 80 |  | 16.1 | 26.4 | 37.4 | 20.1 |  | 0.8629 | 1.4246 | 1.8173 |  | 2.733E-04 | 3.319E-11 | 1.046E-08 |
| 6 | Q01995 | TAGLN | Transgelin | 201 |  | 41 | 101 | 108 | 18 |  | 15.3 | 37.7 | 40.3 | 6.7 |  | 1.4357 | 1.5904 | 0.3576 |  | 3.264E-08 | 7.104E-10 | 9.050E-01 |
| 7 | P55083 | MFAP4 | Microfibril-associated glycoprotein 4 | 255 |  | 14 | 58 | 62 | 30 |  | 8.5 | 35.4 | 37.8 | 18.3 |  | 2.1174 | 2.2704 | 2.5304 |  | 2.274E-08 | 1.407E-09 | 2.049E-06 |
| 8 | Q15063 | POSTN | Periostin | 836 |  | 30 | 58 | 80 | 11 |  | 16.8 | 32.4 | 44.7 | 6.1 |  | 1.0813 | 1.5970 | 0.1401 |  | 9.873E-04 | 1.029E-07 | 5.756E-01 |
| 9 | Q13228 | SELENBP1 | Selenium-binding protein 1 | 472 |  | 73 | 92 | 132 | 34 |  | 22.1 | 27.8 | 39.9 | 10.3 |  | 0.4868 | 1.0632 | 0.4177 |  | 5.203E-02 | 1.165E-06 | 9.710E-01 |
| 10 | P09960 | LTA4H | Leukotriene A-4 hydrolase | 611 |  | 5 | 11 | 31 | 2 |  | 10.2 | 22.4 | 63.3 | 4.1 |  | 1.1276 | 2.5839 | 0.5478 |  | 1.536E-01 | 2.782E-06 | 8.382E-01 |
| 11 | Q16555 | DPYSL2 | Dihydropyrimidinase-related protein 2 | 572 |  | 60 | 78 | 111 | 61 |  | 19.4 | 25.2 | 35.8 | 19.7 |  | 0.5296 | 1.0928 | 1.5213 |  | 5.206E-02 | 4.994E-06 | 4.390E-05 |
| 12 | P12429 | ANXA3 | Annexin A3 | 323 |  | 37 | 49 | 74 | 49 |  | 17.7 | 23.4 | 35.4 | 23.4 |  | 0.5510 | 1.1939 | 1.8909 |  | 1.149E-01 | 5.819E-05 | 3.464E-06 |
| 13 | P20231 | TPSB2 | Tryptase beta-2 | 275 |  | 18 | 29 | 42 | 0 |  | 20.2 | 32.6 | 47.2 | 0.0 |  | 0.8092 | 1.3844 | -2.4548 |  | 7.996E-02 | 6.137E-04 | 1.475E-03 |
| 14 | P04179 | SOD2 | Superoxide dismutase [Mn], mitochondrial | 222 |  | 10 | 14 | 30 | 0 |  | 18.5 | 25.9 | 55.6 | 0.0 |  | 0.5955 | 1.6901 | -1.6794 |  | 4.133E-01 | 6.635E-04 | 3.355E-02 |
| 15 | P00352 | ALDH1A1 | Retinal dehydrogenase 1 | 501 |  | 14 | 17 | 36 | 25 |  | 15.2 | 18.5 | 39.1 | 27.2 |  | 0.4157 | 1.5048 | 2.2780 |  | 5.536E-01 | 6.820E-04 | 8.363E-05 |
| 16 | Q99439 | CNN2 | Calponin-2 | 309 |  | 5 | 19 | 21 | 0 |  | 11.1 | 42.2 | 46.7 | 0.0 |  | 1.8533 | 2.0477 | -0.8311 |  | 3.422E-03 | 9.500E-04 | 2.422E-01 |
| 17 | P27658 | COL8A1 | Collagen alpha-1(VIII) chain | 744 |  | 23 | 38 | 47 | 27 |  | 17.0 | 28.1 | 34.8 | 20.0 |  | 0.8522 | 1.2091 | 1.7146 |  | 3.340E-02 | 1.249E-03 | 2.268E-03 |
| 18 | Q14894 | CRYM | Thiomorpholine-carboxylate dehydrogenase | 314 |  | 10 | 14 | 27 | 0 |  | 19.6 | 27.5 | 52.9 | 0.0 |  | 0.5955 | 1.5443 | -1.6794 |  | 4.133E-01 | 2.693E-03 | 3.355E-02 |
| 19 | P31146 | CORO1A | Coronin-1A | 461 |  | 4 | 7 | 17 | 8 |  | 11.1 | 19.4 | 47.2 | 22.2 |  | 0.8086 | 2.0131 | 2.3094 |  | 4.574E-01 | 3.257E-03 | 3.087E-02 |
| 20 | P24752 | ACAT1 | Acetyl-CoA acetyltransferase, mitochondrial | 427 |  | 24 | 33 | 45 | 2 |  | 23.1 | 31.7 | 43.3 | 1.9 |  | 0.5969 | 1.0895 | -1.4677 |  | 1.683E-01 | 4.038E-03 | 6.488E-03 |
| 21 | P23526 | AHCY | Adenosylhomocysteinase | 432 |  | 6 | 8 | 17 | 0 |  | 19.4 | 25.8 | 54.8 | 0.0 |  | 0.5079 | 1.5473 | -1.0453 |  | 6.716E-01 | 1.743E-02 | 1.626E-01 |
| 22 | P04233 | CD74 | HLA class II histocompatibility antigen gamma chain | 296 |  | 6 | 12 | 17 | 0 |  | 17.1 | 34.3 | 48.6 | 0.0 |  | 1.0267 | 1.5473 | -1.0453 |  | 1.729E-01 | 1.743E-02 | 1.626E-01 |
| 23 | P23142 | FBLN1 | Fibulin-1 | 703 |  | 11 | 9 | 23 | 0 |  | 25.6 | 20.9 | 53.5 | 0.0 |  | -0.1009 | 1.2008 | -1.8023 |  | 9.707E-01 | 2.653E-02 | 2.267E-02 |
| 24 | P00387 | CYB5R3 | NADH-cytochrome b5 reductase 3 | 301 |  | 11 | 11 | 23 | 5 |  | 22.0 | 22.0 | 46.0 | 10.0 |  | 0.1564 | 1.2008 | 0.5205 |  | 9.863E-01 | 2.653E-02 | 8.449E-01 |
| 25 | P45880 | VDAC2 | Voltage-dependent anion-selective channel protein 2 | 294 |  | 2 | 8 | 9 | 0 |  | 10.5 | 42.1 | 47.4 | 0.0 |  | 1.6657 | 1.8723 | 0.1125 |  | 8.061E-02 | 4.182E-02 | 8.264E-01 |
| 26 | Q15019 | SEPT2 | Septin-2 | 361 |  | 12 | 17 | 23 | 0 |  | 23.1 | 32.7 | 44.2 | 0.0 |  | 0.6186 | 1.0875 | -1.9156 |  | 3.324E-01 | 4.317E-02 | 1.533E-02 |
|  |  |  |  |  |  |  |  |  |  |  |  |  |  |  |  |  |  |  |  |  |  |  |
|  |  |  |  |  |  |  |  |  |  |  |  |  |  |  |  |  |  |  |  |  |  |  |

Table S2.　 Proteins characteristic to MIA, which were under *p* < 0.05 (MIA vs LPIA), *Rsc* > 1 (LPIA vs. MIA), and [MIA] greater than [LPIA] and [AIS].

|  |  |  |  |  |  | Spectral Counts *(SpCs*) | | | |  | Relative % thougtout Stages | | | |  | Fold change in log2 (*R_SC_*) | | |  | *p*-value in G-test | | |
| --- | --- | --- | --- | --- | --- | --- | --- | --- | --- | --- | --- | --- | --- | --- | --- | --- | --- | --- | --- | --- | --- | --- |
| No | Accession Number/Code | Gene ID | Description | Protein length (AA) |  | LPIA | MIA | AIIS | Total |  | [LPIA] | [MIA] | [AIS] | [pN] |  | LPIA vs MIA | LPIA vs AIS | LPIA vs pN |  | LPIA vs MIA | LPIA vs AIS | LPIA vs pN |
|  |  |  |  |  |  |  |  |  |  |  |  |  |  |  |  |  |  |  |  |  |  |  |
| 1 | P98160 | HSPG2 | Basement membrane-specific heparan sulfate proteoglycan core protein | 4391 |  | 125 | 273 | 208 | 136 |  | 16.8 | 36.8 | 28.0 | 18.3 |  | 1.2864 | 0.9507 | 1.6274 |  | 9.125E-17 | 3.352E-08 | 3.115E-11 |
| 2 | P15502 | ELN | Elastin | 786 |  | 90 | 163 | 132 | 93 |  | 18.8 | 34.1 | 27.6 | 19.5 |  | 1.0099 | 0.7647 | 1.5482 |  | 1.669E-07 | 3.507E-04 | 2.227E-07 |
| 3 | Q8IWL1 | SFTPA2 | Pulmonary surfactant-associated protein A2 | 248 |  | 7 | 35 | 16 | 22 |  | 8.8 | 43.8 | 20.0 | 27.5 |  | 2.2937 | 1.2795 | 2.9892 |  | 4.227E-06 | 5.189E-02 | 3.435E-06 |
| 4 | P40199 | CEACAM6 | Carcinoembryonic antigen-related cell adhesion molecule 6 | 344 |  | 26 | 56 | 13 | 0 |  | 27.4 | 58.9 | 13.7 | 0.0 |  | 1.2295 | -0.7213 | -2.9567 |  | 2.997E-04 | 1.091E-01 | 6.569E-05 |
| 5 | Q05682 | CALD1 | Caldesmon | 563 |  | 12 | 35 | 34 | 0 |  | 14.8 | 43.2 | 42.0 | 0.0 |  | 1.6099 | 1.6280 | -1.9156 |  | 3.764E-04 | 4.345E-04 | 1.533E-02 |
| 6 | Q14767 | LTBP2 | Latent-transforming growth factor beta-binding protein 2 | 1821 |  | 5 | 21 | 20 | 0 |  | 10.9 | 45.7 | 43.5 | 0.0 |  | 1.9893 | 1.9813 | -0.8311 |  | 1.182E-03 | 1.644E-03 | 2.422E-01 |
| 7 | P11216 | PYGB | Glycogen phosphorylase, brain form | 843 |  | 26 | 48 | 38 | 0 |  | 23.2 | 42.9 | 33.9 | 0.0 |  | 1.0118 | 0.7422 | -2.9567 |  | 4.835E-03 | 6.984E-02 | 6.569E-05 |
| 8 | P29373 | CRABP2 | Cellular retinoic acid-binding protein 2 | 138 |  | 1 | 10 | 0 | 0 |  | 9.1 | 90.9 | 0.0 | 0.0 |  | 2.4788 | -0.6334 | 0.6431 |  | 7.387E-03 | 9.567E-01 | 7.108E-01 |
| 9 | Q8WWI1 | LMO7 | LIM domain only protein 7 | 1557 |  | 3 | 14 | 0 | 1 |  | 16.7 | 77.8 | 0.0 | 5.6 |  | 2.0003 | -1.5510 | 0.5736 |  | 7.503E-03 | 2.641E-01 | 8.187E-01 |
| 10 | P17844 | DDX5 | Probable ATP-dependent RNA helicase DDX5 | 614 |  | 15 | 32 | 17 | 4 |  | 22.1 | 47.1 | 25.0 | 5.9 |  | 1.1905 | 0.3824 | -0.1391 |  | 8.222E-03 | 6.285E-01 | 4.204E-01 |
| 11 | Q12905 | ILF2 | Interleukin enhancer-binding factor 2 | 390 |  | 8 | 21 | 15 | 0 |  | 18.2 | 47.7 | 34.1 | 0.0 |  | 1.4235 | 1.0282 | -1.3969 |  | 1.295E-02 | 1.291E-01 | 7.367E-02 |
| 12 | Q99714 | HSD17B10 | 3-hydroxyacyl-CoA dehydrogenase type-2 | 261 |  | 1 | 9 | 5 | 0 |  | 6.7 | 60.0 | 33.3 | 0.0 |  | 2.3445 | 1.6889 | 0.6431 |  | 1.420E-02 | 1.666E-01 | 7.108E-01 |
| 13 | Q9H4A4 | RNPEP | Aminopeptidase B | 650 |  | 8 | 20 | 0 | 0 |  | 28.6 | 71.4 | 0.0 | 0.0 |  | 1.3571 | -2.6733 | -1.3969 |  | 2.000E-02 | 1.012E-02 | 7.367E-02 |
| 14 | Q96NY7 | CLIC6 | Chloride intracellular channel protein 6 | 704 |  | 9 | 21 | 0 | 0 |  | 30.0 | 70.0 | 0.0 | 0.0 |  | 1.2753 | -2.8215 | -1.5450 |  | 2.386E-02 | 5.301E-03 | 4.970E-02 |
| 15 | P54819 | AK2 | Adenylate kinase 2, mitochondrial | 232 |  | 10 | 22 | 21 | 0 |  | 18.9 | 41.5 | 39.6 | 0.0 |  | 1.2045 | 1.1994 | -1.6794 |  | 2.783E-02 | 3.462E-02 | 3.355E-02 |
|  |  |  |  |  |  |  |  |  |  |  |  |  |  |  |  |  |  |  |  |  |  |  |

Table S3. The list of total 840 proteins identified.

|  |  |  |  |  |  | Spectral Counts *(SpCs*) | | | |  | Relative % thougtout Stages | | | |  | Fold change in log2 (*R_SC_*) | | |  | *p*-value in G-test | | |
| --- | --- | --- | --- | --- | --- | --- | --- | --- | --- | --- | --- | --- | --- | --- | --- | --- | --- | --- | --- | --- | --- | --- |
| No | Accession Number/Code | Gene ID | Description | Protein length (AA) |  | LPIA | MIA | AIIS | Total |  | [LPIA] | [MIA] | [AIS] | [pN] |  | LPIA vs MIA | LPIA vs AIS | LPIA vs pN |  | LPIA vs MIA | LPIA vs AIS | LPIA vs pN |
| 1 | hi|HIP000323690|pHIT000107737|HIX0201467| | Band 7 protein family protein | Band 7 protein family protein;NA;cat | 2858 |  | 77 | 0 | 0 | 0 |  | 100.0 | 0.0 | 0.0 | 0.0 |  | -5.8165 | -5.7581 | -4.4816 |  | 2.333E-22 | 5.796E-22 | 1.669E-13 |
| 2 | P01892 | HLA-A | HLA class I histocompatibility antigen, A-2 alpha chain | 365 |  | 34 | 0 | 0 | 0 |  | 100.0 | 0.0 | 0.0 | 0.0 |  | -4.6634 | -4.6050 | -3.3285 |  | 3.745E-10 | 5.587E-10 | 2.939E-06 |
| 3 | P10253 | GAA | Lysosomal alpha-glucosidase | 952 |  | 26 | 0 | 0 | 0 |  | 100.0 | 0.0 | 0.0 | 0.0 |  | -4.2915 | -4.2332 | -2.9567 |  | 6.988E-08 | 9.483E-08 | 6.569E-05 |
| 4 | Q07960 | ARHGAP1 | Rho GTPase-activating protein 1 | 439 |  | 20 | 0 | 0 | 0 |  | 100.0 | 0.0 | 0.0 | 0.0 |  | -3.9324 | -3.8740 | -2.5975 |  | 3.535E-06 | 4.468E-06 | 6.770E-04 |
| 5 | P06865 | HEXA | Beta-hexosaminidase subunit alpha | 529 |  | 19 | 0 | 0 | 0 |  | 100.0 | 0.0 | 0.0 | 0.0 |  | -3.8628 | -3.8044 | -2.5279 |  | 6.799E-06 | 8.493E-06 | 9.991E-04 |
| 6 | P53007 | SLC25A1 | Tricarboxylate transport protein, mitochondrial | 311 |  | 18 | 0 | 0 | 0 |  | 100.0 | 0.0 | 0.0 | 0.0 |  | -3.7897 | -3.7313 | -2.4548 |  | 1.308E-05 | 1.615E-05 | 1.475E-03 |
| 7 | P36871 | PGM1 | Phosphoglucomutase-1 | 562 |  | 18 | 0 | 0 | 0 |  | 100.0 | 0.0 | 0.0 | 0.0 |  | -3.7897 | -3.7313 | -2.4548 |  | 1.308E-05 | 1.615E-05 | 1.475E-03 |
| 8 | A0A0A6YYG9 | ARPC4-TTLL3 | ARPC4-TTLL3 fusion protein | 625 |  | 18 | 0 | 0 | 0 |  | 100.0 | 0.0 | 0.0 | 0.0 |  | -3.7897 | -3.7313 | -2.4548 |  | 1.308E-05 | 1.615E-05 | 1.475E-03 |
| 9 | Q96HE7 | ERO1L | ERO1-like protein alpha | 468 |  | 16 | 0 | 0 | 0 |  | 100.0 | 0.0 | 0.0 | 0.0 |  | -3.6313 | -3.5729 | -2.2964 |  | 4.844E-05 | 5.840E-05 | 3.215E-03 |
| 10 | P58107 | EPPK1 | Epiplakin | 5090 |  | 15 | 0 | 0 | 0 |  | 100.0 | 0.0 | 0.0 | 0.0 |  | -3.5451 | -3.4867 | -2.2102 |  | 9.324E-05 | 1.111E-04 | 4.748E-03 |
| 11 | P49588 | AARS | Alanine--tRNA ligase, cytoplasmic | 968 |  | 15 | 0 | 0 | 0 |  | 100.0 | 0.0 | 0.0 | 0.0 |  | -3.5451 | -3.4867 | -2.2102 |  | 9.324E-05 | 1.111E-04 | 4.748E-03 |
| 12 | P16615 | ATP2A2 | Sarcoplasmic/endoplasmic reticulum calcium ATPase 2 | 997 |  | 15 | 0 | 0 | 0 |  | 100.0 | 0.0 | 0.0 | 0.0 |  | -3.5451 | -3.4867 | -2.2102 |  | 9.324E-05 | 1.111E-04 | 4.748E-03 |
| 13 | P62873 | GNB1 | Guanine nucleotide-binding protein G(I)/G(S)/G(T) subunit beta-1 | 340 |  | 14 | 0 | 0 | 0 |  | 100.0 | 0.0 | 0.0 | 0.0 |  | -3.4534 | -3.3950 | -2.1185 |  | 1.795E-04 | 2.114E-04 | 7.015E-03 |
| 14 | O43760 | SYNGR2 | Synaptogyrin-2 | 224 |  | 14 | 0 | 0 | 0 |  | 100.0 | 0.0 | 0.0 | 0.0 |  | -3.4534 | -3.3950 | -2.1185 |  | 1.795E-04 | 2.114E-04 | 7.015E-03 |
| 15 | O60701 | UGDH | UDP-glucose 6-dehydrogenase | 494 |  | 12 | 0 | 0 | 0 |  | 100.0 | 0.0 | 0.0 | 0.0 |  | -3.2504 | -3.1920 | -1.9156 |  | 6.660E-04 | 7.658E-04 | 1.533E-02 |
| 16 | Q5T2N8 | ATAD3C | ATPase family AAA domain-containing protein 3C | 411 |  | 11 | 0 | 0 | 0 |  | 100.0 | 0.0 | 0.0 | 0.0 |  | -3.1372 | -3.0788 | -1.8023 |  | 1.284E-03 | 1.459E-03 | 2.267E-02 |
| 17 | P46782 | RPS5 | 40S ribosomal protein S5 | 204 |  | 11 | 0 | 0 | 0 |  | 100.0 | 0.0 | 0.0 | 0.0 |  | -3.1372 | -3.0788 | -1.8023 |  | 1.284E-03 | 1.459E-03 | 2.267E-02 |
| 18 | Q96AE4 | FUBP1 | Far upstream element-binding protein 1 | 644 |  | 10 | 0 | 0 | 0 |  | 100.0 | 0.0 | 0.0 | 0.0 |  | -3.0142 | -2.9559 | -1.6794 |  | 2.475E-03 | 2.780E-03 | 3.355E-02 |
| 19 | P46783 | RPS10 | 40S ribosomal protein S10 | 165 |  | 10 | 0 | 0 | 0 |  | 100.0 | 0.0 | 0.0 | 0.0 |  | -3.0142 | -2.9559 | -1.6794 |  | 2.475E-03 | 2.780E-03 | 3.355E-02 |
| 20 | P17516 | AKR1C4 | Aldo-keto reductase family 1 member C4 | 323 |  | 10 | 0 | 0 | 0 |  | 100.0 | 0.0 | 0.0 | 0.0 |  | -3.0142 | -2.9559 | -1.6794 |  | 2.475E-03 | 2.780E-03 | 3.355E-02 |
| 21 | P15531 | NME1 | Nucleoside diphosphate kinase A | 152 |  | 10 | 0 | 0 | 0 |  | 100.0 | 0.0 | 0.0 | 0.0 |  | -3.0142 | -2.9559 | -1.6794 |  | 2.475E-03 | 2.780E-03 | 3.355E-02 |
| 22 | P15428 | HPGD | 15-hydroxyprostaglandin dehydrogenase [NAD(+)] | 266 |  | 10 | 0 | 0 | 0 |  | 100.0 | 0.0 | 0.0 | 0.0 |  | -3.0142 | -2.9559 | -1.6794 |  | 2.475E-03 | 2.780E-03 | 3.355E-02 |
| 23 | O43776 | NARS | Asparagine--tRNA ligase, cytoplasmic | 548 |  | 10 | 0 | 0 | 0 |  | 100.0 | 0.0 | 0.0 | 0.0 |  | -3.0142 | -2.9559 | -1.6794 |  | 2.475E-03 | 2.780E-03 | 3.355E-02 |
| 24 | P68036 | UBE2L3 | Ubiquitin-conjugating enzyme E2 L3 | 154 |  | 9 | 0 | 0 | 0 |  | 100.0 | 0.0 | 0.0 | 0.0 |  | -2.8799 | -2.8215 | -1.5450 |  | 4.776E-03 | 5.301E-03 | 4.970E-02 |
| 25 | P54802 | NAGLU | Alpha-N-acetylglucosaminidase | 743 |  | 9 | 0 | 0 | 0 |  | 100.0 | 0.0 | 0.0 | 0.0 |  | -2.8799 | -2.8215 | -1.5450 |  | 4.776E-03 | 5.301E-03 | 4.970E-02 |
| 26 | P11586 | MTHFD1 | C-1-tetrahydrofolate synthase, cytoplasmic | 935 |  | 9 | 0 | 0 | 0 |  | 100.0 | 0.0 | 0.0 | 0.0 |  | -2.8799 | -2.8215 | -1.5450 |  | 4.776E-03 | 5.301E-03 | 4.970E-02 |
| 27 | P02790 | HPX | Hemopexin | 462 |  | 9 | 0 | 0 | 0 |  | 100.0 | 0.0 | 0.0 | 0.0 |  | -2.8799 | -2.8215 | -1.5450 |  | 4.776E-03 | 5.301E-03 | 4.970E-02 |
| 28 | A8MWD9 | SNRPF | Small nuclear ribonucleoprotein G-like protein | 76 |  | 9 | 0 | 0 | 0 |  | 100.0 | 0.0 | 0.0 | 0.0 |  | -2.8799 | -2.8215 | -1.5450 |  | 4.776E-03 | 5.301E-03 | 4.970E-02 |
| 29 | Q9Y3U8 | RPL36 | 60S ribosomal protein L36 | 105 |  | 8 | 0 | 0 | 0 |  | 100.0 | 0.0 | 0.0 | 0.0 |  | -2.7317 | -2.6733 | -1.3969 |  | 9.224E-03 | 1.012E-02 | 7.367E-02 |
| 30 | Q14247 | CTTN | Src substrate cortactin | 634 |  | 8 | 0 | 0 | 0 |  | 100.0 | 0.0 | 0.0 | 0.0 |  | -2.7317 | -2.6733 | -1.3969 |  | 9.224E-03 | 1.012E-02 | 7.367E-02 |
| 31 | P62491 | RAB11A | Ras-related protein Rab-11A | 216 |  | 8 | 0 | 0 | 0 |  | 100.0 | 0.0 | 0.0 | 0.0 |  | -2.7317 | -2.6733 | -1.3969 |  | 9.224E-03 | 1.012E-02 | 7.367E-02 |
| 32 | P56192 | MARS | Methionine--tRNA ligase, cytoplasmic | 900 |  | 8 | 0 | 0 | 0 |  | 100.0 | 0.0 | 0.0 | 0.0 |  | -2.7317 | -2.6733 | -1.3969 |  | 9.224E-03 | 1.012E-02 | 7.367E-02 |
| 33 | P12830 | CDH1 | Cadherin-1 | 882 |  | 8 | 0 | 0 | 0 |  | 100.0 | 0.0 | 0.0 | 0.0 |  | -2.7317 | -2.6733 | -1.3969 |  | 9.224E-03 | 1.012E-02 | 7.367E-02 |
| 34 | P05166 | PCCB | Propionyl-CoA carboxylase beta chain, mitochondrial | 559 |  | 8 | 0 | 0 | 0 |  | 100.0 | 0.0 | 0.0 | 0.0 |  | -2.7317 | -2.6733 | -1.3969 |  | 9.224E-03 | 1.012E-02 | 7.367E-02 |
| 35 | O75347 | TBCA | Tubulin-specific chaperone A | 108 |  | 8 | 0 | 0 | 0 |  | 100.0 | 0.0 | 0.0 | 0.0 |  | -2.7317 | -2.6733 | -1.3969 |  | 9.224E-03 | 1.012E-02 | 7.367E-02 |
| 36 | O43684 | BUB3 | Mitotic checkpoint protein BUB3 | 328 |  | 8 | 0 | 0 | 0 |  | 100.0 | 0.0 | 0.0 | 0.0 |  | -2.7317 | -2.6733 | -1.3969 |  | 9.224E-03 | 1.012E-02 | 7.367E-02 |
| 37 | Q9UM22 | EPDR1 | Mammalian ependymin-related protein 1 | 224 |  | 7 | 0 | 0 | 0 |  | 100.0 | 0.0 | 0.0 | 0.0 |  | -2.5666 | -2.5082 | -1.2318 |  | 1.783E-02 | 1.933E-02 | 1.093E-01 |
| 38 | Q14376 | GALE | UDP-glucose 4-epimerase | 348 |  | 7 | 0 | 0 | 0 |  | 100.0 | 0.0 | 0.0 | 0.0 |  | -2.5666 | -2.5082 | -1.2318 |  | 1.783E-02 | 1.933E-02 | 1.093E-01 |
| 39 | P48637 | GSS | Glutathione synthetase | 474 |  | 7 | 0 | 0 | 0 |  | 100.0 | 0.0 | 0.0 | 0.0 |  | -2.5666 | -2.5082 | -1.2318 |  | 1.783E-02 | 1.933E-02 | 1.093E-01 |
| 40 | P47897 | QARS | Glutamine--tRNA ligase | 775 |  | 7 | 0 | 0 | 0 |  | 100.0 | 0.0 | 0.0 | 0.0 |  | -2.5666 | -2.5082 | -1.2318 |  | 1.783E-02 | 1.933E-02 | 1.093E-01 |
| 41 | P15941 | MUC1 | Mucin-1 | 475 |  | 7 | 0 | 0 | 0 |  | 100.0 | 0.0 | 0.0 | 0.0 |  | -2.5666 | -2.5082 | -1.2318 |  | 1.783E-02 | 1.933E-02 | 1.093E-01 |
| 42 | O60763 | USO1 | General vesicular transport factor p115 | 962 |  | 7 | 0 | 0 | 0 |  | 100.0 | 0.0 | 0.0 | 0.0 |  | -2.5666 | -2.5082 | -1.2318 |  | 1.783E-02 | 1.933E-02 | 1.093E-01 |
| 43 | Q9Y3I0 | C22orf28 | tRNA-splicing ligase RtcB homolog | 505 |  | 6 | 0 | 0 | 0 |  | 100.0 | 0.0 | 0.0 | 0.0 |  | -2.3801 | -2.3217 | -1.0453 |  | 3.453E-02 | 3.698E-02 | 1.626E-01 |
| 44 | Q9NRV9 | HEBP1 | Heme-binding protein 1 | 189 |  | 6 | 0 | 0 | 0 |  | 100.0 | 0.0 | 0.0 | 0.0 |  | -2.3801 | -2.3217 | -1.0453 |  | 3.453E-02 | 3.698E-02 | 1.626E-01 |
| 45 | Q9BPW8 | NIPSNAP1 | Protein NipSnap homolog 1 | 284 |  | 6 | 0 | 0 | 0 |  | 100.0 | 0.0 | 0.0 | 0.0 |  | -2.3801 | -2.3217 | -1.0453 |  | 3.453E-02 | 3.698E-02 | 1.626E-01 |
| 46 | P49419 | ALDH7A1 | Alpha-aminoadipic semialdehyde dehydrogenase | 539 |  | 6 | 0 | 0 | 0 |  | 100.0 | 0.0 | 0.0 | 0.0 |  | -2.3801 | -2.3217 | -1.0453 |  | 3.453E-02 | 3.698E-02 | 1.626E-01 |
| 47 | P29590 | PML | Protein PML | 633 |  | 6 | 0 | 0 | 0 |  | 100.0 | 0.0 | 0.0 | 0.0 |  | -2.3801 | -2.3217 | -1.0453 |  | 3.453E-02 | 3.698E-02 | 1.626E-01 |
| 48 | P14868 | DARS | Aspartate--tRNA ligase, cytoplasmic | 501 |  | 6 | 0 | 0 | 0 |  | 100.0 | 0.0 | 0.0 | 0.0 |  | -2.3801 | -2.3217 | -1.0453 |  | 3.453E-02 | 3.698E-02 | 1.626E-01 |
| 49 | P00533 | EGFR | Epidermal growth factor receptor | 705 |  | 6 | 0 | 0 | 0 |  | 100.0 | 0.0 | 0.0 | 0.0 |  | -2.3801 | -2.3217 | -1.0453 |  | 3.453E-02 | 3.698E-02 | 1.626E-01 |
| 50 | Q9Y230 | RUVBL2 | RuvB-like 2 | 463 |  | 5 | 0 | 0 | 0 |  | 100.0 | 0.0 | 0.0 | 0.0 |  | -2.1659 | -2.1076 | -0.8311 |  | 6.700E-02 | 7.092E-02 | 2.422E-01 |
| 51 | Q13283 | G3BP1 | Ras GTPase-activating protein-binding protein 1 | 466 |  | 5 | 0 | 0 | 0 |  | 100.0 | 0.0 | 0.0 | 0.0 |  | -2.1659 | -2.1076 | -0.8311 |  | 6.700E-02 | 7.092E-02 | 2.422E-01 |
| 52 | Q04637 | EIF4G1 | Eukaryotic translation initiation factor 4 gamma 1 | 1599 |  | 5 | 0 | 0 | 0 |  | 100.0 | 0.0 | 0.0 | 0.0 |  | -2.1659 | -2.1076 | -0.8311 |  | 6.700E-02 | 7.092E-02 | 2.422E-01 |
| 53 | P46777 | RPL5 | 60S ribosomal protein L5 | 297 |  | 5 | 0 | 0 | 0 |  | 100.0 | 0.0 | 0.0 | 0.0 |  | -2.1659 | -2.1076 | -0.8311 |  | 6.700E-02 | 7.092E-02 | 2.422E-01 |
| 54 | P31751 | AKT2 | RAC-beta serine/threonine-protein kinase | 481 |  | 5 | 0 | 0 | 0 |  | 100.0 | 0.0 | 0.0 | 0.0 |  | -2.1659 | -2.1076 | -0.8311 |  | 6.700E-02 | 7.092E-02 | 2.422E-01 |
| 55 | P18859 | ATP5J | ATP synthase-coupling factor 6, mitochondrial | 108 |  | 5 | 0 | 0 | 0 |  | 100.0 | 0.0 | 0.0 | 0.0 |  | -2.1659 | -2.1076 | -0.8311 |  | 6.700E-02 | 7.092E-02 | 2.422E-01 |
| 56 | P11279 | LAMP1 | Lysosome-associated membrane glycoprotein 1 | 417 |  | 5 | 0 | 0 | 0 |  | 100.0 | 0.0 | 0.0 | 0.0 |  | -2.1659 | -2.1076 | -0.8311 |  | 6.700E-02 | 7.092E-02 | 2.422E-01 |
| 57 | P10155 | TROVE2 | 60 kDa SS-A/Ro ribonucleoprotein | 538 |  | 5 | 0 | 0 | 0 |  | 100.0 | 0.0 | 0.0 | 0.0 |  | -2.1659 | -2.1076 | -0.8311 |  | 6.700E-02 | 7.092E-02 | 2.422E-01 |
| 58 | P05165 | PCCA | Propionyl-CoA carboxylase alpha chain, mitochondrial | 728 |  | 5 | 0 | 0 | 0 |  | 100.0 | 0.0 | 0.0 | 0.0 |  | -2.1659 | -2.1076 | -0.8311 |  | 6.700E-02 | 7.092E-02 | 2.422E-01 |
| 59 | Q9Y2B0 | CNPY2 | Protein canopy homolog 2 | 182 |  | 4 | 0 | 0 | 0 |  | 100.0 | 0.0 | 0.0 | 0.0 |  | -1.9143 | -1.8560 | -0.5795 |  | 1.304E-01 | 1.365E-01 | 3.621E-01 |
| 60 | Q9UJS0 | SLC25A13 | Calcium-binding mitochondrial carrier protein Aralar2 | 675 |  | 4 | 0 | 0 | 0 |  | 100.0 | 0.0 | 0.0 | 0.0 |  | -1.9143 | -1.8560 | -0.5795 |  | 1.304E-01 | 1.365E-01 | 3.621E-01 |
| 61 | Q9HCY8 | S100A14 | Protein S100-A14 | 104 |  | 4 | 0 | 0 | 0 |  | 100.0 | 0.0 | 0.0 | 0.0 |  | -1.9143 | -1.8560 | -0.5795 |  | 1.304E-01 | 1.365E-01 | 3.621E-01 |
| 62 | Q9HB40 | SCPEP1 | Retinoid-inducible serine carboxypeptidase | 452 |  | 4 | 0 | 0 | 0 |  | 100.0 | 0.0 | 0.0 | 0.0 |  | -1.9143 | -1.8560 | -0.5795 |  | 1.304E-01 | 1.365E-01 | 3.621E-01 |
| 63 | Q9H0W9 | C11orf54 | Ester hydrolase C11orf54 | 315 |  | 4 | 0 | 0 | 0 |  | 100.0 | 0.0 | 0.0 | 0.0 |  | -1.9143 | -1.8560 | -0.5795 |  | 1.304E-01 | 1.365E-01 | 3.621E-01 |
| 64 | Q9BXW7 | CECR5 | Cat eye syndrome critical region protein 5 | 423 |  | 4 | 0 | 0 | 0 |  | 100.0 | 0.0 | 0.0 | 0.0 |  | -1.9143 | -1.8560 | -0.5795 |  | 1.304E-01 | 1.365E-01 | 3.621E-01 |
| 65 | Q9BQE5 | APOL2 | Apolipoprotein L2 | 337 |  | 4 | 0 | 0 | 0 |  | 100.0 | 0.0 | 0.0 | 0.0 |  | -1.9143 | -1.8560 | -0.5795 |  | 1.304E-01 | 1.365E-01 | 3.621E-01 |
| 66 | Q7KZF4 | SND1 | Staphylococcal nuclease domain-containing protein 1 | 910 |  | 4 | 0 | 0 | 0 |  | 100.0 | 0.0 | 0.0 | 0.0 |  | -1.9143 | -1.8560 | -0.5795 |  | 1.304E-01 | 1.365E-01 | 3.621E-01 |
| 67 | Q15274 | QPRT | Nicotinate-nucleotide pyrophosphorylase [carboxylating] | 297 |  | 4 | 0 | 0 | 0 |  | 100.0 | 0.0 | 0.0 | 0.0 |  | -1.9143 | -1.8560 | -0.5795 |  | 1.304E-01 | 1.365E-01 | 3.621E-01 |
| 68 | Q14258 | TRIM25 | E3 ubiquitin/ISG15 ligase TRIM25 | 630 |  | 4 | 0 | 0 | 0 |  | 100.0 | 0.0 | 0.0 | 0.0 |  | -1.9143 | -1.8560 | -0.5795 |  | 1.304E-01 | 1.365E-01 | 3.621E-01 |
| 69 | P98161 | PKD1 | Polycystin-1 | 4087 |  | 4 | 0 | 0 | 0 |  | 100.0 | 0.0 | 0.0 | 0.0 |  | -1.9143 | -1.8560 | -0.5795 |  | 1.304E-01 | 1.365E-01 | 3.621E-01 |
| 70 | P78346 | RPP30 | Ribonuclease P protein subunit p30 | 268 |  | 4 | 0 | 0 | 0 |  | 100.0 | 0.0 | 0.0 | 0.0 |  | -1.9143 | -1.8560 | -0.5795 |  | 1.304E-01 | 1.365E-01 | 3.621E-01 |
| 71 | P56557 | TMEM50B | Transmembrane protein 50B | 158 |  | 4 | 0 | 0 | 0 |  | 100.0 | 0.0 | 0.0 | 0.0 |  | -1.9143 | -1.8560 | -0.5795 |  | 1.304E-01 | 1.365E-01 | 3.621E-01 |
| 72 | P07686 | HEXB | Beta-hexosaminidase subunit beta | 556 |  | 4 | 0 | 0 | 0 |  | 100.0 | 0.0 | 0.0 | 0.0 |  | -1.9143 | -1.8560 | -0.5795 |  | 1.304E-01 | 1.365E-01 | 3.621E-01 |
| 73 | O95777 | NAA38 | N-alpha-acetyltransferase 38, NatC auxiliary subunit | 96 |  | 4 | 0 | 0 | 0 |  | 100.0 | 0.0 | 0.0 | 0.0 |  | -1.9143 | -1.8560 | -0.5795 |  | 1.304E-01 | 1.365E-01 | 3.621E-01 |
| 74 | O95372 | LYPLA2 | Acyl-protein thioesterase 2 | 231 |  | 4 | 0 | 0 | 0 |  | 100.0 | 0.0 | 0.0 | 0.0 |  | -1.9143 | -1.8560 | -0.5795 |  | 1.304E-01 | 1.365E-01 | 3.621E-01 |
| 75 | O15382 | BCAT2 | Branched-chain-amino-acid aminotransferase, mitochondrial | 392 |  | 4 | 0 | 0 | 0 |  | 100.0 | 0.0 | 0.0 | 0.0 |  | -1.9143 | -1.8560 | -0.5795 |  | 1.304E-01 | 1.365E-01 | 3.621E-01 |
| 76 | O15347 | HMGB3 | High mobility group protein B3 | 200 |  | 4 | 0 | 0 | 0 |  | 100.0 | 0.0 | 0.0 | 0.0 |  | -1.9143 | -1.8560 | -0.5795 |  | 1.304E-01 | 1.365E-01 | 3.621E-01 |
| 77 | HIP000043995 | HIP000043995 | RNA-directed DNA polymerase (reverse transcriptase) family protein;NA;cat | 136 |  | 4 | 0 | 0 | 0 |  | 100.0 | 0.0 | 0.0 | 0.0 |  | -1.9143 | -1.8560 | -0.5795 |  | 1.304E-01 | 1.365E-01 | 3.621E-01 |
| 78 | Q9Y3A6 | TMED5 | Transmembrane emp24 domain-containing protein 5 | 229 |  | 3 | 0 | 0 | 0 |  | 100.0 | 0.0 | 0.0 | 0.0 |  | -1.6094 | -1.5510 | -0.2746 |  | 2.554E-01 | 2.641E-01 | 5.442E-01 |
| 79 | Q9Y6E2 | BZW2 | Basic leucine zipper and W2 domain-containing protein 2 | 419 |  | 3 | 0 | 0 | 0 |  | 100.0 | 0.0 | 0.0 | 0.0 |  | -1.6094 | -1.5510 | -0.2746 |  | 2.554E-01 | 2.641E-01 | 5.442E-01 |
| 80 | Q9UJU6 | DBNL | Drebrin-like protein | 430 |  | 3 | 0 | 0 | 0 |  | 100.0 | 0.0 | 0.0 | 0.0 |  | -1.6094 | -1.5510 | -0.2746 |  | 2.554E-01 | 2.641E-01 | 5.442E-01 |
| 81 | Q9UHD8 | SEPT9 | Septin-9 | 586 |  | 3 | 0 | 0 | 0 |  | 100.0 | 0.0 | 0.0 | 0.0 |  | -1.6094 | -1.5510 | -0.2746 |  | 2.554E-01 | 2.641E-01 | 5.442E-01 |
| 82 | Q9P0J0 | NDUFA13 | NADH dehydrogenase [ubiquinone] 1 alpha subcomplex subunit 13 | 227 |  | 3 | 0 | 0 | 0 |  | 100.0 | 0.0 | 0.0 | 0.0 |  | -1.6094 | -1.5510 | -0.2746 |  | 2.554E-01 | 2.641E-01 | 5.442E-01 |
| 83 | Q9H7B2 | RPF2 | Ribosome production factor 2 homolog | 306 |  | 3 | 0 | 0 | 0 |  | 100.0 | 0.0 | 0.0 | 0.0 |  | -1.6094 | -1.5510 | -0.2746 |  | 2.554E-01 | 2.641E-01 | 5.442E-01 |
| 84 | Q9H0R4 | HDHD2 | Haloacid dehalogenase-like hydrolase domain-containing protein 2 | 259 |  | 3 | 0 | 0 | 0 |  | 100.0 | 0.0 | 0.0 | 0.0 |  | -1.6094 | -1.5510 | -0.2746 |  | 2.554E-01 | 2.641E-01 | 5.442E-01 |
| 85 | Q92597 | NDRG1 | Protein NDRG1 | 394 |  | 3 | 0 | 0 | 0 |  | 100.0 | 0.0 | 0.0 | 0.0 |  | -1.6094 | -1.5510 | -0.2746 |  | 2.554E-01 | 2.641E-01 | 5.442E-01 |
| 86 | Q8WU39 | PACAP | Plasma cell-induced resident endoplasmic reticulum protein | 189 |  | 3 | 0 | 0 | 0 |  | 100.0 | 0.0 | 0.0 | 0.0 |  | -1.6094 | -1.5510 | -0.2746 |  | 2.554E-01 | 2.641E-01 | 5.442E-01 |
| 87 | Q86V81 | THOC4 | THO complex subunit 4 | 264 |  | 3 | 0 | 0 | 0 |  | 100.0 | 0.0 | 0.0 | 0.0 |  | -1.6094 | -1.5510 | -0.2746 |  | 2.554E-01 | 2.641E-01 | 5.442E-01 |
| 88 | Q6PCE3 | PGM2L1 | Glucose 1,6-bisphosphate synthase | 622 |  | 3 | 0 | 0 | 0 |  | 100.0 | 0.0 | 0.0 | 0.0 |  | -1.6094 | -1.5510 | -0.2746 |  | 2.554E-01 | 2.641E-01 | 5.442E-01 |
| 89 | Q32P28 | LEPRE1 | Prolyl 3-hydroxylase 1 | 697 |  | 3 | 0 | 0 | 0 |  | 100.0 | 0.0 | 0.0 | 0.0 |  | -1.6094 | -1.5510 | -0.2746 |  | 2.554E-01 | 2.641E-01 | 5.442E-01 |
| 90 | Q15437 | SEC23B | Protein transport protein Sec23B | 767 |  | 3 | 0 | 0 | 0 |  | 100.0 | 0.0 | 0.0 | 0.0 |  | -1.6094 | -1.5510 | -0.2746 |  | 2.554E-01 | 2.641E-01 | 5.442E-01 |
| 91 | Q13200 | PSMD2 | 26S proteasome non-ATPase regulatory subunit 2 | 908 |  | 3 | 0 | 0 | 0 |  | 100.0 | 0.0 | 0.0 | 0.0 |  | -1.6094 | -1.5510 | -0.2746 |  | 2.554E-01 | 2.641E-01 | 5.442E-01 |
| 92 | Q03518 | TAP1 | Antigen peptide transporter 1 | 808 |  | 3 | 0 | 0 | 0 |  | 100.0 | 0.0 | 0.0 | 0.0 |  | -1.6094 | -1.5510 | -0.2746 |  | 2.554E-01 | 2.641E-01 | 5.442E-01 |
| 93 | P56537 | EIF6 | Eukaryotic translation initiation factor 6 | 245 |  | 3 | 0 | 0 | 0 |  | 100.0 | 0.0 | 0.0 | 0.0 |  | -1.6094 | -1.5510 | -0.2746 |  | 2.554E-01 | 2.641E-01 | 5.442E-01 |
| 94 | P55327 | TPD52 | Tumor protein D52 | 224 |  | 3 | 0 | 0 | 0 |  | 100.0 | 0.0 | 0.0 | 0.0 |  | -1.6094 | -1.5510 | -0.2746 |  | 2.554E-01 | 2.641E-01 | 5.442E-01 |
| 95 | P46821 | MAP1B | Microtubule-associated protein 1B | 2468 |  | 3 | 0 | 0 | 0 |  | 100.0 | 0.0 | 0.0 | 0.0 |  | -1.6094 | -1.5510 | -0.2746 |  | 2.554E-01 | 2.641E-01 | 5.442E-01 |
| 96 | P35606 | COPB2 | Coatomer subunit beta_ | 906 |  | 3 | 0 | 0 | 0 |  | 100.0 | 0.0 | 0.0 | 0.0 |  | -1.6094 | -1.5510 | -0.2746 |  | 2.554E-01 | 2.641E-01 | 5.442E-01 |
| 97 | P20618 | PSMB1 | Proteasome subunit beta type-1 | 241 |  | 3 | 0 | 0 | 0 |  | 100.0 | 0.0 | 0.0 | 0.0 |  | -1.6094 | -1.5510 | -0.2746 |  | 2.554E-01 | 2.641E-01 | 5.442E-01 |
| 98 | P09497 | CLTB | Clathrin light chain B | 229 |  | 3 | 0 | 0 | 0 |  | 100.0 | 0.0 | 0.0 | 0.0 |  | -1.6094 | -1.5510 | -0.2746 |  | 2.554E-01 | 2.641E-01 | 5.442E-01 |
| 99 | P04196 | HRG | Histidine-rich glycoprotein | 525 |  | 3 | 0 | 0 | 0 |  | 100.0 | 0.0 | 0.0 | 0.0 |  | -1.6094 | -1.5510 | -0.2746 |  | 2.554E-01 | 2.641E-01 | 5.442E-01 |
| 100 | P00491 | PNP | Purine nucleoside phosphorylase | 289 |  | 3 | 0 | 0 | 0 |  | 100.0 | 0.0 | 0.0 | 0.0 |  | -1.6094 | -1.5510 | -0.2746 |  | 2.554E-01 | 2.641E-01 | 5.442E-01 |
| 101 | O95340 | PAPSS2 | Bifunctional 3_-phosphoadenosine 5_-phosphosulfate synthase 2 | 614 |  | 3 | 0 | 0 | 0 |  | 100.0 | 0.0 | 0.0 | 0.0 |  | -1.6094 | -1.5510 | -0.2746 |  | 2.554E-01 | 2.641E-01 | 5.442E-01 |
| 102 | O43852 | CALU | Calumenin | 315 |  | 3 | 0 | 0 | 0 |  | 100.0 | 0.0 | 0.0 | 0.0 |  | -1.6094 | -1.5510 | -0.2746 |  | 2.554E-01 | 2.641E-01 | 5.442E-01 |
| 103 | Q9Y224 | C14orf166 | UPF0568 protein C14orf166 | 244 |  | 2 | 0 | 0 | 0 |  | 100.0 | 0.0 | 0.0 | 0.0 |  | -1.2223 | -1.1640 | 0.1125 |  | 5.056E-01 | 5.166E-01 | 8.264E-01 |
| 104 | Q9UBS4 | DNAJB11 | DnaJ homolog subfamily B member 11 | 358 |  | 2 | 0 | 0 | 0 |  | 100.0 | 0.0 | 0.0 | 0.0 |  | -1.2223 | -1.1640 | 0.1125 |  | 5.056E-01 | 5.166E-01 | 8.264E-01 |
| 105 | Q9NX46 | ADPRHL2 | Poly(ADP-ribose) glycohydrolase ARH3 | 363 |  | 2 | 0 | 0 | 0 |  | 100.0 | 0.0 | 0.0 | 0.0 |  | -1.2223 | -1.1640 | 0.1125 |  | 5.056E-01 | 5.166E-01 | 8.264E-01 |
| 106 | Q9H0E2 | TOLLIP | Toll-interacting protein | 274 |  | 2 | 0 | 0 | 0 |  | 100.0 | 0.0 | 0.0 | 0.0 |  | -1.2223 | -1.1640 | 0.1125 |  | 5.056E-01 | 5.166E-01 | 8.264E-01 |
| 107 | Q9BY50 | SEC11C | Signal peptidase complex catalytic subunit SEC11C | 192 |  | 2 | 0 | 0 | 0 |  | 100.0 | 0.0 | 0.0 | 0.0 |  | -1.2223 | -1.1640 | 0.1125 |  | 5.056E-01 | 5.166E-01 | 8.264E-01 |
| 108 | Q99873 | PRMT1 | Protein arginine N-methyltransferase 1 | 357 |  | 2 | 0 | 0 | 0 |  | 100.0 | 0.0 | 0.0 | 0.0 |  | -1.2223 | -1.1640 | 0.1125 |  | 5.056E-01 | 5.166E-01 | 8.264E-01 |
| 109 | Q86UP2 | KTN1 | Kinectin | 1357 |  | 2 | 0 | 0 | 0 |  | 100.0 | 0.0 | 0.0 | 0.0 |  | -1.2223 | -1.1640 | 0.1125 |  | 5.056E-01 | 5.166E-01 | 8.264E-01 |
| 110 | Q16543 | CDC37 | Hsp90 co-chaperone Cdc37 | 378 |  | 2 | 0 | 0 | 0 |  | 100.0 | 0.0 | 0.0 | 0.0 |  | -1.2223 | -1.1640 | 0.1125 |  | 5.056E-01 | 5.166E-01 | 8.264E-01 |
| 111 | Q13488 | TCIRG1 | V-type proton ATPase 116 kDa subunit a isoform 3 | 830 |  | 2 | 0 | 0 | 0 |  | 100.0 | 0.0 | 0.0 | 0.0 |  | -1.2223 | -1.1640 | 0.1125 |  | 5.056E-01 | 5.166E-01 | 8.264E-01 |
| 112 | Q13057 | COASY | Bifunctional coenzyme A synthase | 564 |  | 2 | 0 | 0 | 0 |  | 100.0 | 0.0 | 0.0 | 0.0 |  | -1.2223 | -1.1640 | 0.1125 |  | 5.056E-01 | 5.166E-01 | 8.264E-01 |
| 113 | Q06210 | GFPT1 | Glucosamine--fructose-6-phosphate aminotransferase [isomerizing] 1 | 681 |  | 2 | 0 | 0 | 0 |  | 100.0 | 0.0 | 0.0 | 0.0 |  | -1.2223 | -1.1640 | 0.1125 |  | 5.056E-01 | 5.166E-01 | 8.264E-01 |
| 114 | P53384 | NUBP1 | Cytosolic Fe-S cluster assembly factor NUBP1 | 320 |  | 2 | 0 | 0 | 0 |  | 100.0 | 0.0 | 0.0 | 0.0 |  | -1.2223 | -1.1640 | 0.1125 |  | 5.056E-01 | 5.166E-01 | 8.264E-01 |
| 115 | P41567 | EIF1 | Eukaryotic translation initiation factor 1 | 113 |  | 2 | 0 | 0 | 0 |  | 100.0 | 0.0 | 0.0 | 0.0 |  | -1.2223 | -1.1640 | 0.1125 |  | 5.056E-01 | 5.166E-01 | 8.264E-01 |
| 116 | P26583 | HMGB2 | High mobility group protein B2 | 209 |  | 2 | 0 | 0 | 0 |  | 100.0 | 0.0 | 0.0 | 0.0 |  | -1.2223 | -1.1640 | 0.1125 |  | 5.056E-01 | 5.166E-01 | 8.264E-01 |
| 117 | P26196 | DDX6 | Probable ATP-dependent RNA helicase DDX6 | 483 |  | 2 | 0 | 0 | 0 |  | 100.0 | 0.0 | 0.0 | 0.0 |  | -1.2223 | -1.1640 | 0.1125 |  | 5.056E-01 | 5.166E-01 | 8.264E-01 |
| 118 | P17987 | TCP1 | T-complex protein 1 subunit alpha | 556 |  | 2 | 0 | 0 | 0 |  | 100.0 | 0.0 | 0.0 | 0.0 |  | -1.2223 | -1.1640 | 0.1125 |  | 5.056E-01 | 5.166E-01 | 8.264E-01 |
| 119 | P14923 | JUP | Junction plakoglobin | 745 |  | 2 | 0 | 0 | 0 |  | 100.0 | 0.0 | 0.0 | 0.0 |  | -1.2223 | -1.1640 | 0.1125 |  | 5.056E-01 | 5.166E-01 | 8.264E-01 |
| 120 | P11766 | ADH5 | Alcohol dehydrogenase class-3 | 374 |  | 2 | 0 | 0 | 0 |  | 100.0 | 0.0 | 0.0 | 0.0 |  | -1.2223 | -1.1640 | 0.1125 |  | 5.056E-01 | 5.166E-01 | 8.264E-01 |
| 121 | P02766 | TTR | Transthyretin | 147 |  | 2 | 0 | 0 | 0 |  | 100.0 | 0.0 | 0.0 | 0.0 |  | -1.2223 | -1.1640 | 0.1125 |  | 5.056E-01 | 5.166E-01 | 8.264E-01 |
| 122 | O95433 | AHSA1 | Activator of 90 kDa heat shock protein ATPase homolog 1 | 338 |  | 2 | 0 | 0 | 0 |  | 100.0 | 0.0 | 0.0 | 0.0 |  | -1.2223 | -1.1640 | 0.1125 |  | 5.056E-01 | 5.166E-01 | 8.264E-01 |
| 123 | O60749 | SNX2 | Sorting nexin-2 | 519 |  | 2 | 0 | 0 | 0 |  | 100.0 | 0.0 | 0.0 | 0.0 |  | -1.2223 | -1.1640 | 0.1125 |  | 5.056E-01 | 5.166E-01 | 8.264E-01 |
| 124 | O43598 | RCL | Deoxyribonucleoside 5_-monophosphate N-glycosidase | 174 |  | 2 | 0 | 0 | 0 |  | 100.0 | 0.0 | 0.0 | 0.0 |  | -1.2223 | -1.1640 | 0.1125 |  | 5.056E-01 | 5.166E-01 | 8.264E-01 |
| 125 | O43324 | EEF1E1 | Eukaryotic translation elongation factor 1 epsilon-1 | 174 |  | 2 | 0 | 0 | 0 |  | 100.0 | 0.0 | 0.0 | 0.0 |  | -1.2223 | -1.1640 | 0.1125 |  | 5.056E-01 | 5.166E-01 | 8.264E-01 |
| 126 | HIP000007440 | HIP000007440 | Immunoglobulin V-set domain containing protein;NA;cat | 107 |  | 2 | 0 | 0 | 0 |  | 100.0 | 0.0 | 0.0 | 0.0 |  | -1.2223 | -1.1640 | 0.1125 |  | 5.056E-01 | 5.166E-01 | 8.264E-01 |
| 127 | Q9Y5L4 | TIMM13 | Mitochondrial import inner membrane translocase subunit Tim13 | 95 |  | 1 | 0 | 0 | 0 |  | 100.0 | 0.0 | 0.0 | 0.0 |  | -0.6918 | -0.6334 | 0.6431 |  | 9.666E-01 | 9.567E-01 | 7.108E-01 |
| 128 | Q9Y333_1 | LSM2 | U6 snRNA-associated Sm-like protein LSm2 | 95 |  | 1 | 0 | 0 | 0 |  | 100.0 | 0.0 | 0.0 | 0.0 |  | -0.6918 | -0.6334 | 0.6431 |  | 9.666E-01 | 9.567E-01 | 7.108E-01 |
| 129 | Q9Y262 | EIF3L | Eukaryotic translation initiation factor 3 subunit L | 607 |  | 1 | 0 | 0 | 0 |  | 100.0 | 0.0 | 0.0 | 0.0 |  | -0.6918 | -0.6334 | 0.6431 |  | 9.666E-01 | 9.567E-01 | 7.108E-01 |
| 130 | Q9UBW8 | COPS7A | COP9 signalosome complex subunit 7a | 275 |  | 1 | 0 | 0 | 0 |  | 100.0 | 0.0 | 0.0 | 0.0 |  | -0.6918 | -0.6334 | 0.6431 |  | 9.666E-01 | 9.567E-01 | 7.108E-01 |
| 131 | Q96A33 | CCDC47 | Coiled-coil domain-containing protein 47 | 483 |  | 1 | 0 | 0 | 0 |  | 100.0 | 0.0 | 0.0 | 0.0 |  | -0.6918 | -0.6334 | 0.6431 |  | 9.666E-01 | 9.567E-01 | 7.108E-01 |
| 132 | Q969X5 | ERGIC1 | Endoplasmic reticulum-Golgi intermediate compartment protein 1 | 290 |  | 1 | 0 | 0 | 0 |  | 100.0 | 0.0 | 0.0 | 0.0 |  | -0.6918 | -0.6334 | 0.6431 |  | 9.666E-01 | 9.567E-01 | 7.108E-01 |
| 133 | Q92643 | PIGK | GPI-anchor transamidase | 395 |  | 1 | 0 | 0 | 0 |  | 100.0 | 0.0 | 0.0 | 0.0 |  | -0.6918 | -0.6334 | 0.6431 |  | 9.666E-01 | 9.567E-01 | 7.108E-01 |
| 134 | Q92542 | NCSTN | Nicastrin | 709 |  | 1 | 0 | 0 | 0 |  | 100.0 | 0.0 | 0.0 | 0.0 |  | -0.6918 | -0.6334 | 0.6431 |  | 9.666E-01 | 9.567E-01 | 7.108E-01 |
| 135 | Q8IW45 | CARKD | ATP-dependent (S)-NAD(P)H-hydrate dehydratase | 347 |  | 1 | 0 | 0 | 0 |  | 100.0 | 0.0 | 0.0 | 0.0 |  | -0.6918 | -0.6334 | 0.6431 |  | 9.666E-01 | 9.567E-01 | 7.108E-01 |
| 136 | Q6NVY1 | HIBCH | 3-hydroxyisobutyryl-CoA hydrolase, mitochondrial | 386 |  | 1 | 0 | 0 | 0 |  | 100.0 | 0.0 | 0.0 | 0.0 |  | -0.6918 | -0.6334 | 0.6431 |  | 9.666E-01 | 9.567E-01 | 7.108E-01 |
| 137 | Q3LXA3 | DAK | Bifunctional ATP-dependent dihydroxyacetone kinase/FAD-AMP lyase (cyclizing) | 575 |  | 1 | 0 | 0 | 0 |  | 100.0 | 0.0 | 0.0 | 0.0 |  | -0.6918 | -0.6334 | 0.6431 |  | 9.666E-01 | 9.567E-01 | 7.108E-01 |
| 138 | Q13418_1 | ILK | Integrin-linked protein kinase | 452 |  | 1 | 0 | 0 | 0 |  | 100.0 | 0.0 | 0.0 | 0.0 |  | -0.6918 | -0.6334 | 0.6431 |  | 9.666E-01 | 9.567E-01 | 7.108E-01 |
| 139 | Q12907 | LMAN2 | Vesicular integral-membrane protein VIP36 | 356 |  | 1 | 0 | 0 | 0 |  | 100.0 | 0.0 | 0.0 | 0.0 |  | -0.6918 | -0.6334 | 0.6431 |  | 9.666E-01 | 9.567E-01 | 7.108E-01 |
| 140 | P62942 | FKBP1A | Peptidyl-prolyl cis-trans isomerase FKBP1A | 108 |  | 1 | 0 | 0 | 0 |  | 100.0 | 0.0 | 0.0 | 0.0 |  | -0.6918 | -0.6334 | 0.6431 |  | 9.666E-01 | 9.567E-01 | 7.108E-01 |
| 141 | P55209 | NAP1L1 | Nucleosome assembly protein 1-like 1 | 391 |  | 1 | 0 | 0 | 0 |  | 100.0 | 0.0 | 0.0 | 0.0 |  | -0.6918 | -0.6334 | 0.6431 |  | 9.666E-01 | 9.567E-01 | 7.108E-01 |
| 142 | P55145 | MANF | Mesencephalic astrocyte-derived neurotrophic factor | 182 |  | 1 | 0 | 0 | 0 |  | 100.0 | 0.0 | 0.0 | 0.0 |  | -0.6918 | -0.6334 | 0.6431 |  | 9.666E-01 | 9.567E-01 | 7.108E-01 |
| 143 | P37235 | HPCAL1 | Hippocalcin-like protein 1 | 193 |  | 1 | 0 | 0 | 0 |  | 100.0 | 0.0 | 0.0 | 0.0 |  | -0.6918 | -0.6334 | 0.6431 |  | 9.666E-01 | 9.567E-01 | 7.108E-01 |
| 144 | P35914 | HMGCL | Hydroxymethylglutaryl-CoA lyase, mitochondrial | 325 |  | 1 | 0 | 0 | 0 |  | 100.0 | 0.0 | 0.0 | 0.0 |  | -0.6918 | -0.6334 | 0.6431 |  | 9.666E-01 | 9.567E-01 | 7.108E-01 |
| 145 | P35573 | AGL | Glycogen debranching enzyme | 1532 |  | 1 | 0 | 0 | 0 |  | 100.0 | 0.0 | 0.0 | 0.0 |  | -0.6918 | -0.6334 | 0.6431 |  | 9.666E-01 | 9.567E-01 | 7.108E-01 |
| 146 | P34896 | SHMT1 | Serine hydroxymethyltransferase, cytosolic | 483 |  | 1 | 0 | 0 | 0 |  | 100.0 | 0.0 | 0.0 | 0.0 |  | -0.6918 | -0.6334 | 0.6431 |  | 9.666E-01 | 9.567E-01 | 7.108E-01 |
| 147 | P29966 | MARCKS | Myristoylated alanine-rich C-kinase substrate | 332 |  | 1 | 0 | 0 | 0 |  | 100.0 | 0.0 | 0.0 | 0.0 |  | -0.6918 | -0.6334 | 0.6431 |  | 9.666E-01 | 9.567E-01 | 7.108E-01 |
| 148 | P29144 | TPP2 | Tripeptidyl-peptidase 2 | 1249 |  | 1 | 0 | 0 | 0 |  | 100.0 | 0.0 | 0.0 | 0.0 |  | -0.6918 | -0.6334 | 0.6431 |  | 9.666E-01 | 9.567E-01 | 7.108E-01 |
| 149 | P26639 | TARS | Threonine--tRNA ligase, cytoplasmic | 723 |  | 1 | 0 | 0 | 0 |  | 100.0 | 0.0 | 0.0 | 0.0 |  | -0.6918 | -0.6334 | 0.6431 |  | 9.666E-01 | 9.567E-01 | 7.108E-01 |
| 150 | P14324 | FDPS | Farnesyl pyrophosphate synthase | 419 |  | 1 | 0 | 0 | 0 |  | 100.0 | 0.0 | 0.0 | 0.0 |  | -0.6918 | -0.6334 | 0.6431 |  | 9.666E-01 | 9.567E-01 | 7.108E-01 |
| 151 | P10620 | MGST1 | Microsomal glutathione S-transferase 1 | 155 |  | 1 | 0 | 0 | 0 |  | 100.0 | 0.0 | 0.0 | 0.0 |  | -0.6918 | -0.6334 | 0.6431 |  | 9.666E-01 | 9.567E-01 | 7.108E-01 |
| 152 | O95861 | BPNT1 | 3_(2_),5_-bisphosphate nucleotidase 1 | 308 |  | 1 | 0 | 0 | 0 |  | 100.0 | 0.0 | 0.0 | 0.0 |  | -0.6918 | -0.6334 | 0.6431 |  | 9.666E-01 | 9.567E-01 | 7.108E-01 |
| 153 | O76021 | RSL1D1 | Ribosomal L1 domain-containing protein 1 | 490 |  | 1 | 0 | 0 | 0 |  | 100.0 | 0.0 | 0.0 | 0.0 |  | -0.6918 | -0.6334 | 0.6431 |  | 9.666E-01 | 9.567E-01 | 7.108E-01 |
| 154 | O75489 | NDUFS3 | NADH dehydrogenase [ubiquinone] iron-sulfur protein 3, mitochondrial | 264 |  | 1 | 0 | 0 | 0 |  | 100.0 | 0.0 | 0.0 | 0.0 |  | -0.6918 | -0.6334 | 0.6431 |  | 9.666E-01 | 9.567E-01 | 7.108E-01 |
| 155 | O43920 | NDUFS5 | NADH dehydrogenase [ubiquinone] iron-sulfur protein 5 | 106 |  | 1 | 0 | 0 | 0 |  | 100.0 | 0.0 | 0.0 | 0.0 |  | -0.6918 | -0.6334 | 0.6431 |  | 9.666E-01 | 9.567E-01 | 7.108E-01 |
| 156 | O00267 | SUPT5H | Transcription elongation factor SPT5 | 1087 |  | 1 | 0 | 0 | 0 |  | 100.0 | 0.0 | 0.0 | 0.0 |  | -0.6918 | -0.6334 | 0.6431 |  | 9.666E-01 | 9.567E-01 | 7.108E-01 |
| 157 | Q562R1 | ACTBL2 | Beta-actin-like protein 2 | 376 |  | 228 | 0 | 0 | 0 |  | 100.0 | 0.0 | 0.0 | 0.0 |  | -7.3764 | -7.3180 | -6.0415 |  | < 1.0E-40 | 0.000E+00 | 5.270E-39 |
| 158 | Q9BQE3 | TUBA1C | Tubulin alpha-1C chain | 449 |  | 213 | 0 | 0 | 0 |  | 100.0 | 0.0 | 0.0 | 0.0 |  | -7.2779 | -7.2194 | -5.9430 |  | <1.0E-40 | 0.000E+00 | 1.821E-36 |
| 159 | Q02218 | OGDH | 2-oxoglutarate dehydrogenase, mitochondrial | 1023 |  | 16 | 0 | 1 | 0 |  | 94.1 | 0.0 | 5.9 | 0.0 |  | -3.6313 | -2.7248 | -2.2964 |  | 4.844E-05 | 5.452E-04 | 3.215E-03 |
| 160 | P46977 | STT3A | Dolichyl-diphosphooligosaccharide--protein glycosyltransferase subunit STT3A | 705 |  | 12 | 1 | 0 | 0 |  | 92.3 | 7.7 | 0.0 | 0.0 |  | -2.4024 | -3.1920 | -1.9156 |  | 4.912E-03 | 7.658E-04 | 1.533E-02 |
| 161 | P17858 | PFKL | 6-phosphofructokinase, liver type | 780 |  | 22 | 0 | 2 | 0 |  | 91.7 | 0.0 | 8.3 | 0.0 |  | -4.0623 | -2.6252 | -2.7274 |  | 9.555E-07 | 9.432E-05 | 3.110E-04 |
| 162 | Q15363 | TMED2 | Transmembrane emp24 domain-containing protein 2 | 201 |  | 10 | 1 | 0 | 0 |  | 90.9 | 9.1 | 0.0 | 0.0 |  | -2.1662 | -2.9559 | -1.6794 |  | 1.569E-02 | 2.780E-03 | 3.355E-02 |
| 163 | O43488 | AKR7A2 | Aflatoxin B1 aldehyde reductase member 2 | 358 |  | 12 | 1 | 1 | 0 |  | 85.7 | 7.1 | 7.1 | 0.0 |  | -2.4024 | -2.3440 | -1.9156 |  | 4.912E-03 | 5.589E-03 | 1.533E-02 |
| 164 | Q8NBJ7 | SUMF2 | Sulfatase-modifying factor 2 | 301 |  | 11 | 0 | 2 | 0 |  | 84.6 | 0.0 | 15.4 | 0.0 |  | -3.1372 | -1.7001 | -1.8023 |  | 1.284E-03 | 3.461E-02 | 2.267E-02 |
| 165 | P31948 | STIP1 | Stress-induced-phosphoprotein 1 | 543 |  | 27 | 0 | 5 | 0 |  | 84.4 | 0.0 | 15.6 | 0.0 |  | -4.3436 | -1.9629 | -3.0087 |  | 3.635E-08 | 3.433E-04 | 4.454E-05 |
| 166 | Q15233 | NONO | Non-POU domain-containing octamer-binding protein | 471 |  | 32 | 0 | 6 | 0 |  | 84.2 | 0.0 | 15.8 | 0.0 |  | -4.5790 | -1.9841 | -3.2442 |  | 1.384E-09 | 9.101E-05 | 6.389E-06 |
| 167 | Q3ZCQ8 | TIMM50 | Mitochondrial import inner membrane translocase subunit TIM50 | 353 |  | 5 | 0 | 1 | 0 |  | 83.3 | 0.0 | 16.7 | 0.0 |  | -2.1659 | -1.2595 | -0.8311 |  | 6.700E-02 | 2.613E-01 | 2.422E-01 |
| 168 | P11177 | PDHB | Pyruvate dehydrogenase E1 component subunit beta, mitochondrial | 359 |  | 5 | 0 | 1 | 0 |  | 83.3 | 0.0 | 16.7 | 0.0 |  | -2.1659 | -1.2595 | -0.8311 |  | 6.700E-02 | 2.613E-01 | 2.422E-01 |
| 169 | P32322 | PYCR1 | Pyrroline-5-carboxylate reductase 1, mitochondrial | 319 |  | 10 | 2 | 0 | 0 |  | 83.3 | 16.7 | 0.0 | 0.0 |  | -1.6356 | -2.9559 | -1.6794 |  | 5.156E-02 | 2.780E-03 | 3.355E-02 |
| 170 | O60716 | CTNND1 | Catenin delta-1 | 968 |  | 10 | 2 | 0 | 0 |  | 83.3 | 16.7 | 0.0 | 0.0 |  | -1.6356 | -2.9559 | -1.6794 |  | 5.156E-02 | 2.780E-03 | 3.355E-02 |
| 171 | Q07065 | CKAP4 | Cytoskeleton-associated protein 4 | 602 |  | 24 | 0 | 5 | 0 |  | 82.8 | 0.0 | 17.2 | 0.0 |  | -4.1814 | -1.8008 | -2.8466 |  | 2.584E-07 | 1.429E-03 | 1.429E-04 |
| 172 | Q12765 | SCRN1 | Secernin-1 | 414 |  | 9 | 2 | 0 | 0 |  | 81.8 | 18.2 | 0.0 | 0.0 |  | -1.5012 | -2.8215 | -1.5450 |  | 8.450E-02 | 5.301E-03 | 4.970E-02 |
| 173 | Q9H9B4 | SFXN1 | Sideroflexin-1 | 322 |  | 4 | 0 | 1 | 0 |  | 80.0 | 0.0 | 20.0 | 0.0 |  | -1.9143 | -1.0079 | -0.5795 |  | 1.304E-01 | 4.308E-01 | 3.621E-01 |
| 174 | P31942 | HNRNPH3 | Heterogeneous nuclear ribonucleoprotein H3 | 331 |  | 8 | 2 | 0 | 0 |  | 80.0 | 20.0 | 0.0 | 0.0 |  | -1.3531 | -2.6733 | -1.3969 |  | 1.366E-01 | 1.012E-02 | 7.367E-02 |
| 175 | Q96G03 | PGM2 | Phosphoglucomutase-2 | 612 |  | 4 | 1 | 0 | 0 |  | 80.0 | 20.0 | 0.0 | 0.0 |  | -1.0663 | -1.8560 | -0.5795 |  | 4.150E-01 | 1.365E-01 | 3.621E-01 |
| 176 | P80404 | ABAT | 4-aminobutyrate aminotransferase, mitochondrial | 500 |  | 7 | 0 | 2 | 0 |  | 77.8 | 0.0 | 22.2 | 0.0 |  | -2.5666 | -1.1296 | -1.2318 |  | 1.783E-02 | 2.311E-01 | 1.093E-01 |
| 177 | P51688 | SGSH | N-sulphoglucosamine sulphohydrolase | 502 |  | 7 | 2 | 0 | 0 |  | 77.8 | 22.2 | 0.0 | 0.0 |  | -1.1880 | -2.5082 | -1.2318 |  | 2.172E-01 | 1.933E-02 | 1.093E-01 |
| 178 | P15924 | DSP | Desmoplakin | 2871 |  | 7 | 2 | 0 | 0 |  | 77.8 | 22.2 | 0.0 | 0.0 |  | -1.1880 | -2.5082 | -1.2318 |  | 2.172E-01 | 1.933E-02 | 1.093E-01 |
| 179 | Q9NTX5 | ECHDC1 | Ethylmalonyl-CoA decarboxylase | 307 |  | 28 | 7 | 1 | 0 |  | 77.8 | 19.4 | 2.8 | 0.0 |  | -1.6709 | -3.4874 | -3.0590 |  | 1.165E-03 | 4.047E-07 | 3.020E-05 |
| 180 | O14818 | PSMA7 | Proteasome subunit alpha type-7 | 248 |  | 10 | 3 | 0 | 0 |  | 76.9 | 23.1 | 0.0 | 0.0 |  | -1.2485 | -2.9559 | -1.6794 |  | 1.215E-01 | 2.780E-03 | 3.355E-02 |
| 181 | O75340 | PDCD6 | Programmed cell death protein 6 | 191 |  | 30 | 6 | 3 | 0 |  | 76.9 | 15.4 | 7.7 | 0.0 |  | -1.9529 | -2.6653 | -3.1545 |  | 1.776E-04 | 5.333E-06 | 1.389E-05 |
| 182 | Q7Z4W1 | DCXR | L-xylulose reductase | 244 |  | 23 | 7 | 0 | 0 |  | 76.7 | 23.3 | 0.0 | 0.0 |  | -1.4001 | -4.0647 | -2.7882 |  | 9.914E-03 | 6.507E-07 | 2.108E-04 |
| 183 | P42224 | STAT1 | Signal transducer and activator of transcription 1-alpha/beta | 750 |  | 16 | 5 | 0 | 0 |  | 76.2 | 23.8 | 0.0 | 0.0 |  | -1.3090 | -3.5729 | -2.2964 |  | 4.133E-02 | 5.840E-05 | 3.215E-03 |
| 184 | O43272 | PRODH | Proline dehydrogenase 1, mitochondrial | 492 |  | 9 | 0 | 3 | 0 |  | 75.0 | 0.0 | 25.0 | 0.0 |  | -2.8799 | -1.0558 | -1.5450 |  | 4.776E-03 | 2.002E-01 | 4.970E-02 |
| 185 | P43487 | RANBP1 | Ran-specific GTPase-activating protein | 201 |  | 6 | 2 | 0 | 0 |  | 75.0 | 25.0 | 0.0 | 0.0 |  | -1.0015 | -2.3217 | -1.0453 |  | 3.385E-01 | 3.698E-02 | 1.626E-01 |
| 186 | Q13619 | CUL4A | Cullin-4A | 759 |  | 3 | 1 | 0 | 0 |  | 75.0 | 25.0 | 0.0 | 0.0 |  | -0.7614 | -1.5510 | -0.2746 |  | 6.748E-01 | 2.641E-01 | 5.442E-01 |
| 187 | Q13409 | DYNC1I2 | Cytoplasmic dynein 1 intermediate chain 2 | 638 |  | 3 | 1 | 0 | 0 |  | 75.0 | 25.0 | 0.0 | 0.0 |  | -0.7614 | -1.5510 | -0.2746 |  | 6.748E-01 | 2.641E-01 | 5.442E-01 |
| 188 | Q00577 | PURA | Transcriptional activator protein Pur-alpha | 322 |  | 3 | 1 | 0 | 0 |  | 75.0 | 25.0 | 0.0 | 0.0 |  | -0.7614 | -1.5510 | -0.2746 |  | 6.748E-01 | 2.641E-01 | 5.442E-01 |
| 189 | P62241 | RPS8 | 40S ribosomal protein S8 | 208 |  | 3 | 1 | 0 | 0 |  | 75.0 | 25.0 | 0.0 | 0.0 |  | -0.7614 | -1.5510 | -0.2746 |  | 6.748E-01 | 2.641E-01 | 5.442E-01 |
| 190 | P05198 | EIF2S1 | Eukaryotic translation initiation factor 2 subunit 1 | 315 |  | 3 | 1 | 0 | 0 |  | 75.0 | 25.0 | 0.0 | 0.0 |  | -0.7614 | -1.5510 | -0.2746 |  | 6.748E-01 | 2.641E-01 | 5.442E-01 |
| 191 | O00764 | PDXK | Pyridoxal kinase | 312 |  | 11 | 2 | 2 | 0 |  | 73.3 | 13.3 | 13.3 | 0.0 |  | -1.7585 | -1.7001 | -1.8023 |  | 3.109E-02 | 3.461E-02 | 2.267E-02 |
| 192 | P46779 | RPL28 | 60S ribosomal protein L28 | 137 |  | 8 | 0 | 3 | 0 |  | 72.7 | 0.0 | 27.3 | 0.0 |  | -2.7317 | -0.9076 | -1.3969 |  | 9.224E-03 | 2.972E-01 | 7.367E-02 |
| 193 | P11940 | PABPC1 | Polyadenylate-binding protein 1 | 636 |  | 8 | 0 | 3 | 0 |  | 72.7 | 0.0 | 27.3 | 0.0 |  | -2.7317 | -0.9076 | -1.3969 |  | 9.224E-03 | 2.972E-01 | 7.367E-02 |
| 194 | Q09028 | RBBP4 | Histone-binding protein RBBP4 | 425 |  | 8 | 3 | 0 | 0 |  | 72.7 | 27.3 | 0.0 | 0.0 |  | -0.9660 | -2.6733 | -1.3969 |  | 2.787E-01 | 1.012E-02 | 7.367E-02 |
| 195 | O43747 | AP1G1 | AP-1 complex subunit gamma-1 | 822 |  | 5 | 0 | 2 | 0 |  | 71.4 | 0.0 | 28.6 | 0.0 |  | -2.1659 | -0.7289 | -0.8311 |  | 6.700E-02 | 5.360E-01 | 2.422E-01 |
| 196 | Q14498 | RBM39 | RNA-binding protein 39 | 530 |  | 5 | 2 | 0 | 0 |  | 71.4 | 28.6 | 0.0 | 0.0 |  | -0.7873 | -2.1076 | -0.8311 |  | 5.147E-01 | 7.092E-02 | 2.422E-01 |
| 197 | P54920 | NAPA | Alpha-soluble NSF attachment protein | 295 |  | 10 | 3 | 1 | 0 |  | 71.4 | 21.4 | 7.1 | 0.0 |  | -1.2485 | -2.1078 | -1.6794 |  | 1.215E-01 | 1.744E-02 | 3.355E-02 |
| 198 | P54886 | ALDH18A1 | Delta-1-pyrroline-5-carboxylate synthase | 795 |  | 22 | 9 | 0 | 0 |  | 71.0 | 29.0 | 0.0 | 0.0 |  | -1.0260 | -4.0039 | -2.7274 |  | 5.060E-02 | 1.237E-06 | 3.110E-04 |
| 199 | Q99829 | CPNE1 | Copine-1 | 537 |  | 26 | 4 | 7 | 0 |  | 70.3 | 10.8 | 18.9 | 0.0 |  | -2.2209 | -1.5102 | -2.9567 |  | 1.379E-04 | 3.506E-03 | 6.569E-05 |
| 200 | O43175 | PHGDH | D-3-phosphoglycerate dehydrogenase | 533 |  | 7 | 0 | 3 | 0 |  | 70.0 | 0.0 | 30.0 | 0.0 |  | -2.5666 | -0.7425 | -1.2318 |  | 1.783E-02 | 4.318E-01 | 1.093E-01 |
| 201 | P28288 | ABCD3 | ATP-binding cassette sub-family D member 3 | 659 |  | 9 | 0 | 4 | 0 |  | 69.2 | 0.0 | 30.8 | 0.0 |  | -2.8799 | -0.7508 | -1.5450 |  | 4.776E-03 | 3.551E-01 | 4.970E-02 |
| 202 | P09110 | ACAA1 | 3-ketoacyl-CoA thiolase, peroxisomal | 424 |  | 9 | 0 | 4 | 0 |  | 69.2 | 0.0 | 30.8 | 0.0 |  | -2.8799 | -0.7508 | -1.5450 |  | 4.776E-03 | 3.551E-01 | 4.970E-02 |
| 203 | Q9NSD9 | FARSB | Phenylalanine--tRNA ligase beta subunit | 589 |  | 9 | 3 | 1 | 0 |  | 69.2 | 23.1 | 7.7 | 0.0 |  | -1.1141 | -1.9734 | -1.5450 |  | 1.857E-01 | 3.053E-02 | 4.970E-02 |
| 204 | Q02252 | ALDH6A1 | Methylmalonate-semialdehyde dehydrogenase [acylating], mitochondrial | 535 |  | 29 | 4 | 9 | 0 |  | 69.0 | 9.5 | 21.4 | 0.0 |  | -2.3717 | -1.3478 | -3.1076 |  | 2.843E-05 | 4.685E-03 | 2.048E-05 |
| 205 | P31937 | HIBADH | 3-hydroxyisobutyrate dehydrogenase, mitochondrial | 336 |  | 13 | 5 | 1 | 0 |  | 68.4 | 26.3 | 5.3 | 0.0 |  | -1.0332 | -2.4490 | -2.0206 |  | 1.343E-01 | 3.141E-03 | 1.037E-02 |
| 206 | Q8IV08 | PLD3 | Phospholipase D3 | 490 |  | 43 | 20 | 0 | 0 |  | 68.3 | 31.7 | 0.0 | 0.0 |  | -0.9032 | -4.9336 | -3.6571 |  | 1.333E-02 | 1.735E-12 | 8.942E-08 |
| 207 | Q9BR76 | CORO1B | Coronin-1B | 489 |  | 15 | 0 | 7 | 0 |  | 68.2 | 0.0 | 31.8 | 0.0 |  | -3.5451 | -0.7637 | -2.2102 |  | 9.324E-05 | 2.099E-01 | 4.748E-03 |
| 208 | P09972 | ALDOC | Fructose-bisphosphate aldolase C | 364 |  | 15 | 7 | 0 | 0 |  | 68.2 | 31.8 | 0.0 | 0.0 |  | -0.8221 | -3.4867 | -2.2102 |  | 1.897E-01 | 1.111E-04 | 4.748E-03 |
| 209 | P46776 | RPL27A | 60S ribosomal protein L27a | 148 |  | 10 | 0 | 5 | 0 |  | 66.7 | 0.0 | 33.3 | 0.0 |  | -3.0142 | -0.6336 | -1.6794 |  | 2.475E-03 | 4.062E-01 | 3.355E-02 |
| 210 | P43304 | GPD2 | Glycerol-3-phosphate dehydrogenase, mitochondrial | 727 |  | 6 | 0 | 3 | 0 |  | 66.7 | 0.0 | 33.3 | 0.0 |  | -2.3801 | -0.5560 | -1.0453 |  | 3.453E-02 | 6.117E-01 | 1.626E-01 |
| 211 | O43865 | AHCYL1 | Putative adenosylhomocysteinase 2 | 530 |  | 8 | 4 | 0 | 0 |  | 66.7 | 33.3 | 0.0 | 0.0 |  | -0.6611 | -2.6733 | -1.3969 |  | 4.674E-01 | 1.012E-02 | 7.367E-02 |
| 212 | P28065 | PSMB9 | Proteasome subunit beta type-9 | 219 |  | 6 | 3 | 0 | 0 |  | 66.7 | 33.3 | 0.0 | 0.0 |  | -0.6144 | -2.3217 | -1.0453 |  | 5.860E-01 | 3.698E-02 | 1.626E-01 |
| 213 | Q9P1F3 | C6orf115 | Costars family protein ABRACL | 81 |  | 4 | 2 | 0 | 0 |  | 66.7 | 33.3 | 0.0 | 0.0 |  | -0.5357 | -1.8560 | -0.5795 |  | 7.588E-01 | 1.365E-01 | 3.621E-01 |
| 214 | Q96KA5 | CLPTM1L | Cleft lip and palate transmembrane protein 1-like protein | 538 |  | 4 | 2 | 0 | 0 |  | 66.7 | 33.3 | 0.0 | 0.0 |  | -0.5357 | -1.8560 | -0.5795 |  | 7.588E-01 | 1.365E-01 | 3.621E-01 |
| 215 | P43686 | PSMC4 | 26S protease regulatory subunit 6B | 418 |  | 4 | 2 | 0 | 0 |  | 66.7 | 33.3 | 0.0 | 0.0 |  | -0.5357 | -1.8560 | -0.5795 |  | 7.588E-01 | 1.365E-01 | 3.621E-01 |
| 216 | P35244 | RPA3 | Replication protein A 14 kDa subunit | 121 |  | 4 | 2 | 0 | 0 |  | 66.7 | 33.3 | 0.0 | 0.0 |  | -0.5357 | -1.8560 | -0.5795 |  | 7.588E-01 | 1.365E-01 | 3.621E-01 |
| 217 | Q8NEV1 | CSNK2A3 | casein kinase 2, alpha 1 polypeptide-like;HIX0035927 HIT000084452 HIP000098362;cat | 391 |  | 4 | 2 | 0 | 0 |  | 66.7 | 33.3 | 0.0 | 0.0 |  | -0.5357 | -1.8560 | -0.5795 |  | 7.588E-01 | 1.365E-01 | 3.621E-01 |
| 218 | Q9Y3E0 | GOLT1B | Vesicle transport protein GOT1B | 138 |  | 2 | 1 | 0 | 0 |  | 66.7 | 33.3 | 0.0 | 0.0 |  | -0.3743 | -1.1640 | 0.1125 |  | 9.423E-01 | 5.166E-01 | 8.264E-01 |
| 219 | Q9Y2Q3 | GSTK1 | Glutathione S-transferase kappa 1 | 226 |  | 14 | 4 | 3 | 0 |  | 66.7 | 19.0 | 14.3 | 0.0 |  | -1.3827 | -1.6292 | -2.1185 |  | 4.579E-02 | 2.200E-02 | 7.015E-03 |
| 220 | Q86VP6 | CAND1 | Cullin-associated NEDD8-dissociated protein 1 | 1230 |  | 23 | 10 | 2 | 0 |  | 65.7 | 28.6 | 5.7 | 0.0 |  | -0.9525 | -2.6861 | -2.7882 |  | 6.058E-02 | 5.369E-05 | 2.108E-04 |
| 221 | P78527 | PRKDC | DNA-dependent protein kinase catalytic subunit | 4128 |  | 15 | 2 | 6 | 0 |  | 65.2 | 8.7 | 26.1 | 0.0 |  | -2.1664 | -0.9502 | -2.2102 |  | 3.756E-03 | 1.277E-01 | 4.748E-03 |
| 222 | P35221 | CTNNA1 | Catenin alpha-1 | 906 |  | 28 | 5 | 10 | 0 |  | 65.1 | 11.6 | 23.3 | 0.0 |  | -2.0716 | -1.1648 | -3.0590 |  | 1.612E-04 | 1.312E-02 | 3.020E-05 |
| 223 | Q9UHA4 | MAPKSP1 | Ragulator complex protein LAMTOR3 | 124 |  | 9 | 5 | 0 | 0 |  | 64.3 | 35.7 | 0.0 | 0.0 |  | -0.5576 | -2.8215 | -1.5450 |  | 5.162E-01 | 5.301E-03 | 4.970E-02 |
| 224 | Q8NF37 | LPCAT1 | Lysophosphatidylcholine acyltransferase 1 | 534 |  | 70 | 34 | 5 | 0 |  | 64.2 | 31.2 | 4.6 | 0.0 |  | -0.8609 | -3.3002 | -4.3460 |  | 2.310E-03 | 2.628E-14 | 2.524E-12 |
| 225 | P23381 | WARS | Tryptophan--tRNA ligase, cytoplasmic | 471 |  | 16 | 0 | 0 | 9 |  | 64.0 | 0.0 | 0.0 | 36.0 |  | -3.6313 | -3.5729 | 0.7407 |  | 4.844E-05 | 5.840E-05 | 8.377E-01 |
| 226 | Q00325 | SLC25A3 | Phosphate carrier protein, mitochondrial | 362 |  | 14 | 3 | 5 | 0 |  | 63.6 | 13.6 | 22.7 | 0.0 |  | -1.6876 | -1.0727 | -2.1185 |  | 1.929E-02 | 1.026E-01 | 7.015E-03 |
| 227 | P35754 | GLRX | Glutaredoxin-1 | 106 |  | 7 | 3 | 1 | 0 |  | 63.6 | 27.3 | 9.1 | 0.0 |  | -0.8009 | -1.6602 | -1.2318 |  | 4.094E-01 | 9.135E-02 | 1.093E-01 |
| 228 | P07996 | THBS1 | Thrombospondin-1 | 1170 |  | 14 | 8 | 0 | 0 |  | 63.6 | 36.4 | 0.0 | 0.0 |  | -0.5653 | -3.3950 | -2.1185 |  | 3.812E-01 | 2.114E-04 | 7.015E-03 |
| 229 | O75874 | IDH1 | Isocitrate dehydrogenase [NADP] cytoplasmic | 414 |  | 7 | 4 | 0 | 0 |  | 63.6 | 36.4 | 0.0 | 0.0 |  | -0.4959 | -2.5082 | -1.2318 |  | 6.408E-01 | 1.933E-02 | 1.093E-01 |
| 230 | P12270 | TPR | Nucleoprotein TPR | 2349 |  | 14 | 7 | 1 | 0 |  | 63.6 | 31.8 | 4.5 | 0.0 |  | -0.7304 | -2.5469 | -2.1185 |  | 2.591E-01 | 1.758E-03 | 7.015E-03 |
| 231 | P55786 | NPEPPS | Puromycin-sensitive aminopeptidase | 919 |  | 12 | 7 | 0 | 0 |  | 63.2 | 36.8 | 0.0 | 0.0 |  | -0.5275 | -3.1920 | -1.9156 |  | 4.594E-01 | 7.658E-04 | 1.533E-02 |
| 232 | Q9TQE0 | HLA-DRB1 | HLA class II histocompatibility antigen, DRB1-9 beta chain | 266 |  | 20 | 0 | 0 | 12 |  | 62.5 | 0.0 | 0.0 | 37.5 |  | -3.9324 | -3.8740 | 0.8105 |  | 3.535E-06 | 4.468E-06 | 6.432E-01 |
| 233 | P49257 | LMAN1 | Protein ERGIC-53 | 510 |  | 10 | 0 | 6 | 0 |  | 62.5 | 0.0 | 37.5 | 0.0 |  | -3.0142 | -0.4194 | -1.6794 |  | 2.475E-03 | 5.919E-01 | 3.355E-02 |
| 234 | Q13557 | CAMK2D | Calcium/calmodulin-dependent protein kinase type II subunit delta | 478 |  | 5 | 0 | 3 | 0 |  | 62.5 | 0.0 | 37.5 | 0.0 |  | -2.1659 | -0.3418 | -0.8311 |  | 6.700E-02 | 8.408E-01 | 2.422E-01 |
| 235 | Q9NZM1 | MYOF | Myoferlin | 2061 |  | 5 | 1 | 2 | 0 |  | 62.5 | 12.5 | 25.0 | 0.0 |  | -1.3179 | -0.7289 | -0.8311 |  | 2.489E-01 | 5.360E-01 | 2.422E-01 |
| 236 | P38919 | EIF4A3 | Eukaryotic initiation factor 4A-III | 411 |  | 5 | 3 | 0 | 0 |  | 62.5 | 37.5 | 0.0 | 0.0 |  | -0.4002 | -2.1076 | -0.8311 |  | 8.135E-01 | 7.092E-02 | 2.422E-01 |
| 237 | P62753 | RPS6 | 40S ribosomal protein S6 | 249 |  | 13 | 0 | 8 | 0 |  | 61.9 | 0.0 | 38.1 | 0.0 |  | -3.3555 | -0.4090 | -2.0206 |  | 3.457E-04 | 5.300E-01 | 1.037E-02 |
| 238 | Q96I99 | SUCLG2 | Succinyl-CoA ligase [GDP-forming] subunit beta, mitochondrial | 440 |  | 8 | 3 | 2 | 0 |  | 61.5 | 23.1 | 15.4 | 0.0 |  | -0.9660 | -1.2947 | -1.3969 |  | 2.787E-01 | 1.470E-01 | 7.367E-02 |
| 239 | P43307 | SSR1 | Translocon-associated protein subunit alpha | 286 |  | 8 | 4 | 1 | 0 |  | 61.5 | 30.8 | 7.7 | 0.0 |  | -0.6611 | -1.8253 | -1.3969 |  | 4.674E-01 | 5.305E-02 | 7.367E-02 |
| 240 | P13667 | PDIA4 | Protein disulfide-isomerase A4 | 645 |  | 70 | 18 | 26 | 0 |  | 61.4 | 15.8 | 22.8 | 0.0 |  | -1.7348 | -1.1744 | -4.3460 |  | 1.639E-07 | 7.793E-05 | 2.524E-12 |
| 241 | Q9NX63 | CHCHD3 | Coiled-coil-helix-coiled-coil-helix domain-containing protein 3, mitochondrial | 227 |  | 11 | 0 | 7 | 0 |  | 61.1 | 0.0 | 38.9 | 0.0 |  | -3.1372 | -0.3558 | -1.8023 |  | 1.284E-03 | 6.317E-01 | 2.267E-02 |
| 242 | P54136 | RARS | Arginine--tRNA ligase, cytoplasmic | 660 |  | 11 | 7 | 0 | 0 |  | 61.1 | 38.9 | 0.0 | 0.0 |  | -0.4142 | -3.0788 | -1.8023 |  | 5.948E-01 | 1.459E-03 | 2.267E-02 |
| 243 | P07954 | FH | Fumarate hydratase, mitochondrial | 510 |  | 11 | 6 | 1 | 0 |  | 61.1 | 33.3 | 5.6 | 0.0 |  | -0.6007 | -2.2307 | -1.8023 |  | 4.218E-01 | 9.899E-03 | 2.267E-02 |
| 244 | O95994 | AGR2 | Anterior gradient protein 2 homolog | 175 |  | 58 | 23 | 14 | 0 |  | 61.1 | 24.2 | 14.7 | 0.0 |  | -1.1345 | -1.7459 | -4.0792 |  | 5.207E-04 | 1.574E-06 | 2.655E-10 |
| 245 | Q8NFV4 | ABHD11 | Abhydrolase domain-containing protein 11 | 315 |  | 3 | 0 | 2 | 0 |  | 60.0 | 0.0 | 40.0 | 0.0 |  | -1.6094 | -0.1724 | -0.2746 |  | 2.554E-01 | 9.034E-01 | 5.442E-01 |
| 246 | Q14165 | MLEC | Malectin | 292 |  | 3 | 0 | 2 | 0 |  | 60.0 | 0.0 | 40.0 | 0.0 |  | -1.6094 | -0.1724 | -0.2746 |  | 2.554E-01 | 9.034E-01 | 5.442E-01 |
| 247 | Q08257 | CRYZ | Quinone oxidoreductase | 329 |  | 15 | 3 | 7 | 0 |  | 60.0 | 12.0 | 28.0 | 0.0 |  | -1.7793 | -0.7637 | -2.2102 |  | 1.185E-02 | 2.099E-01 | 4.748E-03 |
| 248 | Q9UBR2 | CTSZ | Cathepsin Z | 303 |  | 12 | 3 | 5 | 0 |  | 60.0 | 15.0 | 25.0 | 0.0 |  | -1.4847 | -0.8698 | -1.9156 |  | 4.962E-02 | 2.112E-01 | 1.533E-02 |
| 249 | Q16401 | PSMD5 | 26S proteasome non-ATPase regulatory subunit 5 | 504 |  | 3 | 1 | 1 | 0 |  | 60.0 | 20.0 | 20.0 | 0.0 |  | -0.7614 | -0.7030 | -0.2746 |  | 6.748E-01 | 6.929E-01 | 5.442E-01 |
| 250 | P35222 | CTNNB1 | Catenin beta-1 | 781 |  | 6 | 4 | 0 | 0 |  | 60.0 | 40.0 | 0.0 | 0.0 |  | -0.3095 | -2.3217 | -1.0453 |  | 8.537E-01 | 3.698E-02 | 1.626E-01 |
| 251 | Q9BZZ5 | API5 | Apoptosis inhibitor 5 | 524 |  | 3 | 2 | 0 | 0 |  | 60.0 | 40.0 | 0.0 | 0.0 |  | -0.2308 | -1.5510 | -0.2746 |  | 9.255E-01 | 2.641E-01 | 5.442E-01 |
| 252 | Q9Y4L1 | HYOU1 | Hypoxia up-regulated protein 1 | 999 |  | 31 | 17 | 4 | 0 |  | 59.6 | 32.7 | 7.7 | 0.0 |  | -0.6658 | -2.4058 | -3.2000 |  | 1.093E-01 | 1.337E-05 | 9.421E-06 |
| 253 | P27487 | DPP4 | Dipeptidyl peptidase 4 | 766 |  | 16 | 8 | 3 | 0 |  | 59.3 | 29.6 | 11.1 | 0.0 |  | -0.7432 | -1.8071 | -2.2964 |  | 2.163E-01 | 8.418E-03 | 3.215E-03 |
| 254 | Q9ULC5 | ACSL5 | Long-chain-fatty-acid--CoA ligase 5 | 683 |  | 13 | 6 | 3 | 0 |  | 59.1 | 27.3 | 13.6 | 0.0 |  | -0.8190 | -1.5313 | -2.0206 |  | 2.270E-01 | 3.506E-02 | 1.037E-02 |
| 255 | Q04837 | SSBP1 | Single-stranded DNA-binding protein, mitochondrial | 148 |  | 10 | 3 | 4 | 0 |  | 58.8 | 17.6 | 23.5 | 0.0 |  | -1.2485 | -0.8852 | -1.6794 |  | 1.215E-01 | 2.498E-01 | 3.355E-02 |
| 256 | Q13492 | PICALM | Phosphatidylinositol-binding clathrin assembly protein | 652 |  | 7 | 5 | 0 | 0 |  | 58.3 | 41.7 | 0.0 | 0.0 |  | -0.2443 | -2.5082 | -1.2318 |  | 8.854E-01 | 1.933E-02 | 1.093E-01 |
| 257 | P62314 | SNRPD1 | Small nuclear ribonucleoprotein Sm D1 | 119 |  | 11 | 8 | 0 | 0 |  | 57.9 | 42.1 | 0.0 | 0.0 |  | -0.2491 | -3.0788 | -1.8023 |  | 7.816E-01 | 1.459E-03 | 2.267E-02 |
| 258 | P49748 | ACADVL | Very long-chain specific acyl-CoA dehydrogenase, mitochondrial | 655 |  | 30 | 10 | 12 | 0 |  | 57.7 | 19.2 | 23.1 | 0.0 |  | -1.3188 | -1.0242 | -3.1545 |  | 4.982E-03 | 2.054E-02 | 1.389E-05 |
| 259 | P62847 | RPS24 | 40S ribosomal protein S24 | 133 |  | 4 | 1 | 2 | 0 |  | 57.1 | 14.3 | 28.6 | 0.0 |  | -1.0663 | -0.4773 | -0.5795 |  | 4.150E-01 | 7.820E-01 | 3.621E-01 |
| 260 | Q13765 | NACA | Nascent polypeptide-associated complex subunit alpha | 215 |  | 4 | 2 | 1 | 0 |  | 57.1 | 28.6 | 14.3 | 0.0 |  | -0.5357 | -1.0079 | -0.5795 |  | 7.588E-01 | 4.308E-01 | 3.621E-01 |
| 261 | Q9NUQ9 | FAM49B | Protein FAM49B | 324 |  | 4 | 3 | 0 | 0 |  | 57.1 | 42.9 | 0.0 | 0.0 |  | -0.1486 | -1.8560 | -0.5795 |  | 9.119E-01 | 1.365E-01 | 3.621E-01 |
| 262 | O00232 | PSMD12 | 26S proteasome non-ATPase regulatory subunit 12 | 456 |  | 4 | 3 | 0 | 0 |  | 57.1 | 42.9 | 0.0 | 0.0 |  | -0.1486 | -1.8560 | -0.5795 |  | 9.119E-01 | 1.365E-01 | 3.621E-01 |
| 263 | P50213 | IDH3A | Isocitrate dehydrogenase [NAD] subunit alpha, mitochondrial | 366 |  | 12 | 5 | 4 | 0 |  | 57.1 | 23.8 | 19.0 | 0.0 |  | -0.9282 | -1.1214 | -1.9156 |  | 1.933E-01 | 1.170E-01 | 1.533E-02 |
| 264 | P49327 | FASN | Fatty acid synthase | 2511 |  | 25 | 19 | 0 | 0 |  | 56.8 | 43.2 | 0.0 | 0.0 |  | -0.2183 | -4.1792 | -2.9027 |  | 6.328E-01 | 1.802E-07 | 9.688E-05 |
| 265 | P61313 | RPL15 | 60S ribosomal protein L15 | 204 |  | 18 | 6 | 8 | 0 |  | 56.3 | 18.8 | 25.0 | 0.0 |  | -1.2532 | -0.8432 | -2.4548 |  | 3.682E-02 | 1.309E-01 | 1.475E-03 |
| 266 | Q8NC51 | SERBP1 | Plasminogen activator inhibitor 1 RNA-binding protein | 408 |  | 14 | 7 | 4 | 0 |  | 56.0 | 28.0 | 16.0 | 0.0 |  | -0.7304 | -1.3243 | -2.1185 |  | 2.591E-01 | 5.166E-02 | 7.015E-03 |
| 267 | O94925 | GLS | Glutaminase kidney isoform, mitochondrial | 669 |  | 10 | 0 | 8 | 0 |  | 55.6 | 0.0 | 44.4 | 0.0 |  | -3.0142 | -0.0678 | -1.6794 |  | 2.475E-03 | 9.955E-01 | 3.355E-02 |
| 268 | Q9C005 | DPY30 | Protein dpy-30 homolog | 99 |  | 5 | 4 | 0 | 0 |  | 55.6 | 44.4 | 0.0 | 0.0 |  | -0.0953 | -2.1076 | -0.8311 |  | 9.001E-01 | 7.092E-02 | 2.422E-01 |
| 269 | Q12906 | ILF3 | Interleukin enhancer-binding factor 3 | 706 |  | 16 | 13 | 0 | 0 |  | 55.2 | 44.8 | 0.0 | 0.0 |  | -0.1194 | -3.5729 | -2.2964 |  | 8.835E-01 | 5.840E-05 | 3.215E-03 |
| 270 | Q04917 | YWHAH | 14-3-3 protein eta | 246 |  | 58 | 0 | 48 | 0 |  | 54.7 | 0.0 | 45.3 | 0.0 |  | -5.4140 | -0.0522 | -4.0792 |  | 5.781E-17 | 7.513E-01 | 2.655E-10 |
| 271 | Q6UX73 | C16orf89 | UPF0764 protein C16orf89 | 440 |  | 6 | 1 | 4 | 0 |  | 54.5 | 9.1 | 36.4 | 0.0 |  | -1.5321 | -0.2511 | -1.0453 |  | 1.465E-01 | 8.846E-01 | 1.626E-01 |
| 272 | P31943 | HNRNPH1 | Heterogeneous nuclear ribonucleoprotein H | 449 |  | 13 | 0 | 11 | 0 |  | 54.2 | 0.0 | 45.8 | 0.0 |  | -3.3555 | -0.0035 | -2.0206 |  | 3.457E-04 | 9.509E-01 | 1.037E-02 |
| 273 | Q92616 | GCN1L1 | Translational activator GCN1 | 2671 |  | 7 | 6 | 0 | 0 |  | 53.8 | 46.2 | 0.0 | 0.0 |  | -0.0301 | -2.5082 | -1.2318 |  | 8.801E-01 | 1.933E-02 | 1.093E-01 |
| 274 | Q9BW04 | SARG | Specifically androgen-regulated gene protein | 601 |  | 22 | 16 | 3 | 0 |  | 53.7 | 39.0 | 7.3 | 0.0 |  | -0.2746 | -2.2381 | -2.7274 |  | 5.788E-01 | 3.994E-04 | 3.110E-04 |
| 275 | P21964 | COMT | Catechol O-methyltransferase | 271 |  | 23 | 11 | 9 | 0 |  | 53.5 | 25.6 | 20.9 | 0.0 |  | -0.8296 | -1.0284 | -2.7882 |  | 9.636E-02 | 4.231E-02 | 2.108E-04 |
| 276 | P22695 | UQCRC2 | Cytochrome b-c1 complex subunit 2, mitochondrial | 453 |  | 8 | 2 | 5 | 0 |  | 53.3 | 13.3 | 33.3 | 0.0 |  | -1.3531 | -0.3511 | -1.3969 |  | 1.366E-01 | 7.183E-01 | 7.367E-02 |
| 277 | P05114 | HMGN1 | Non-histone chromosomal protein HMG-14 | 100 |  | 8 | 7 | 0 | 0 |  | 53.3 | 46.7 | 0.0 | 0.0 |  | -0.0088 | -2.6733 | -1.3969 |  | 8.713E-01 | 1.012E-02 | 7.367E-02 |
| 278 | P26373 | RPL13 | 60S ribosomal protein L13 | 211 |  | 34 | 12 | 18 | 0 |  | 53.1 | 18.8 | 28.1 | 0.0 |  | -1.2566 | -0.6589 | -3.3285 |  | 4.003E-03 | 8.765E-02 | 2.939E-06 |
| 279 | P13473 | LAMP2 | Lysosome-associated membrane glycoprotein 2 | 410 |  | 17 | 15 | 0 | 0 |  | 53.1 | 46.9 | 0.0 | 0.0 |  | -0.0112 | -3.6543 | -2.3778 |  | 9.524E-01 | 3.071E-05 | 2.177E-03 |
| 280 | P04066 | FUCA1 | Tissue alpha-L-fucosidase | 466 |  | 19 | 6 | 11 | 0 |  | 52.8 | 16.7 | 30.6 | 0.0 |  | -1.3263 | -0.5108 | -2.5279 |  | 2.467E-02 | 3.228E-01 | 9.991E-04 |
| 281 | P30046 | DDT | D-dopachrome decarboxylase | 118 |  | 10 | 4 | 5 | 0 |  | 52.6 | 21.1 | 26.3 | 0.0 |  | -0.9436 | -0.6336 | -1.6794 |  | 2.314E-01 | 4.062E-01 | 3.355E-02 |
| 282 | P45974 | USP5 | Ubiquitin carboxyl-terminal hydrolase 5 | 858 |  | 10 | 9 | 0 | 0 |  | 52.6 | 47.4 | 0.0 | 0.0 |  | 0.0220 | -2.9559 | -1.6794 |  | 8.553E-01 | 2.780E-03 | 3.355E-02 |
| 283 | P30084 | ECHS1 | Enoyl-CoA hydratase, mitochondrial | 290 |  | 12 | 0 | 11 | 0 |  | 52.2 | 0.0 | 47.8 | 0.0 |  | -3.2504 | 0.1015 | -1.9156 |  | 6.660E-04 | 8.749E-01 | 1.533E-02 |
| 284 | P31947 | SFN | 14-3-3 protein sigma | 248 |  | 60 | 55 | 0 | 0 |  | 52.2 | 47.8 | 0.0 | 0.0 |  | 0.0335 | -5.4037 | -4.1272 |  | 9.399E-01 | 3.176E-17 | 1.222E-10 |
| 285 | Q99880 | HIST1H2BL | Histone H2B type 1-L | 126 |  | 137 | 0 | 126 | 0 |  | 52.1 | 0.0 | 47.9 | 0.0 |  | -6.6412 | 0.0956 | -5.3064 |  | 2.079E-39 | 8.888E-01 | 1.269E-23 |
| 286 | P30153 | PPP2R1A | Serine/threonine-protein phosphatase 2A 65 kDa regulatory subunit A alpha isoform | 589 |  | 13 | 10 | 2 | 0 |  | 52.0 | 40.0 | 8.0 | 0.0 |  | -0.1849 | -1.9184 | -2.0206 |  | 8.282E-01 | 1.250E-02 | 1.037E-02 |
| 287 | P25398 | RPS12 | 40S ribosomal protein S12 | 132 |  | 16 | 12 | 3 | 0 |  | 51.6 | 38.7 | 9.7 | 0.0 |  | -0.2245 | -1.8071 | -2.2964 |  | 7.289E-01 | 8.418E-03 | 3.215E-03 |
| 288 | Q13423 | NNT | NAD(P) transhydrogenase, mitochondrial | 1086 |  | 24 | 15 | 8 | 0 |  | 51.1 | 31.9 | 17.0 | 0.0 |  | -0.4800 | -1.2350 | -2.8466 |  | 3.049E-01 | 1.617E-02 | 1.429E-04 |
| 289 | Q5JNZ5 | RPS26P11 | Putative 40S ribosomal protein S26-like 1 | 115 |  | 9 | 0 | 9 | 0 |  | 50.0 | 0.0 | 50.0 | 0.0 |  | -2.8799 | 0.2148 | -1.5450 |  | 4.776E-03 | 9.955E-01 | 4.970E-02 |
| 290 | Q02978 | SLC25A11 | Mitochondrial 2-oxoglutarate/malate carrier protein | 314 |  | 7 | 0 | 7 | 0 |  | 50.0 | 0.0 | 50.0 | 0.0 |  | -2.5666 | 0.2147 | -1.2318 |  | 1.783E-02 | 9.486E-01 | 1.093E-01 |
| 291 | Q9P0L0 | VAPA | Vesicle-associated membrane protein-associated protein A | 249 |  | 5 | 0 | 5 | 0 |  | 50.0 | 0.0 | 50.0 | 0.0 |  | -2.1659 | 0.2147 | -0.8311 |  | 6.700E-02 | 8.846E-01 | 2.422E-01 |
| 292 | P61019 | RAB2A | Ras-related protein Rab-2A | 212 |  | 5 | 0 | 5 | 0 |  | 50.0 | 0.0 | 50.0 | 0.0 |  | -2.1659 | 0.2147 | -0.8311 |  | 6.700E-02 | 8.846E-01 | 2.422E-01 |
| 293 | P38606 | ATP6V1A | V-type proton ATPase catalytic subunit A | 617 |  | 5 | 0 | 5 | 0 |  | 50.0 | 0.0 | 50.0 | 0.0 |  | -2.1659 | 0.2147 | -0.8311 |  | 6.700E-02 | 8.846E-01 | 2.422E-01 |
| 294 | P15088 | CPA3 | Mast cell carboxypeptidase A | 417 |  | 2 | 0 | 2 | 0 |  | 50.0 | 0.0 | 50.0 | 0.0 |  | -1.2223 | 0.2147 | 0.1125 |  | 5.056E-01 | 6.929E-01 | 8.264E-01 |
| 295 | P47914 | RPL29 | 60S ribosomal protein L29 | 159 |  | 1 | 0 | 1 | 0 |  | 50.0 | 0.0 | 50.0 | 0.0 |  | -0.6918 | 0.2147 | 0.6431 |  | 9.666E-01 | 5.166E-01 | 7.108E-01 |
| 296 | O75131 | CPNE3 | Copine-3 | 537 |  | 8 | 1 | 7 | 0 |  | 50.0 | 6.3 | 43.8 | 0.0 |  | -1.8837 | 0.0496 | -1.3969 |  | 4.883E-02 | 8.335E-01 | 7.367E-02 |
| 297 | P11413 | G6PD | Glucose-6-phosphate 1-dehydrogenase | 515 |  | 17 | 6 | 11 | 0 |  | 50.0 | 17.6 | 32.4 | 0.0 |  | -1.1762 | -0.3607 | -2.3778 |  | 5.436E-02 | 5.083E-01 | 2.177E-03 |
| 298 | P14314 | PRKCSH | Glucosidase 2 subunit beta | 525 |  | 28 | 11 | 17 | 0 |  | 50.0 | 19.6 | 30.4 | 0.0 |  | -1.1003 | -0.4664 | -3.0590 |  | 1.906E-02 | 2.562E-01 | 3.020E-05 |
| 299 | P05023 | ATP1A1 | Sodium/potassium-transporting ATPase subunit alpha-1 | 1023 |  | 22 | 10 | 12 | 0 |  | 50.0 | 22.7 | 27.3 | 0.0 |  | -0.8917 | -0.5971 | -2.7274 |  | 8.341E-02 | 2.153E-01 | 3.110E-04 |
| 300 | O14980 | XPO1 | Exportin-1 | 1071 |  | 2 | 1 | 1 | 0 |  | 50.0 | 25.0 | 25.0 | 0.0 |  | -0.3743 | -0.3159 | 0.1125 |  | 9.423E-01 | 9.251E-01 | 8.264E-01 |
| 301 | P40306 | PSMB10 | Proteasome subunit beta type-10 | 273 |  | 7 | 4 | 3 | 0 |  | 50.0 | 28.6 | 21.4 | 0.0 |  | -0.4959 | -0.7425 | -1.2318 |  | 6.408E-01 | 4.318E-01 | 1.093E-01 |
| 302 | Q9Y6C9 | MTCH2 | Mitochondrial carrier homolog 2 | 303 |  | 1 | 1 | 0 | 0 |  | 50.0 | 50.0 | 0.0 | 0.0 |  | 0.1563 | -0.6334 | 0.6431 |  | 5.056E-01 | 9.567E-01 | 7.108E-01 |
| 303 | Q96HY6 | DDRGK1 | DDRGK domain-containing protein 1 | 314 |  | 1 | 1 | 0 | 0 |  | 50.0 | 50.0 | 0.0 | 0.0 |  | 0.1563 | -0.6334 | 0.6431 |  | 5.056E-01 | 9.567E-01 | 7.108E-01 |
| 304 | Q15293 | RCN1 | Reticulocalbin-1 | 331 |  | 1 | 1 | 0 | 0 |  | 50.0 | 50.0 | 0.0 | 0.0 |  | 0.1563 | -0.6334 | 0.6431 |  | 5.056E-01 | 9.567E-01 | 7.108E-01 |
| 305 | Q9P2J5 | LARS | Leucine--tRNA ligase, cytoplasmic | 1176 |  | 2 | 2 | 0 | 0 |  | 50.0 | 50.0 | 0.0 | 0.0 |  | 0.1563 | -1.1640 | 0.1125 |  | 6.748E-01 | 5.166E-01 | 8.264E-01 |
| 306 | Q9BW60 | ELOVL1 | Elongation of very long chain fatty acids protein 1 | 279 |  | 2 | 2 | 0 | 0 |  | 50.0 | 50.0 | 0.0 | 0.0 |  | 0.1563 | -1.1640 | 0.1125 |  | 6.748E-01 | 5.166E-01 | 8.264E-01 |
| 307 | P17812 | CTPS | CTP synthase 1 | 591 |  | 2 | 2 | 0 | 0 |  | 50.0 | 50.0 | 0.0 | 0.0 |  | 0.1563 | -1.1640 | 0.1125 |  | 6.748E-01 | 5.166E-01 | 8.264E-01 |
| 308 | O14579 | COPE | Coatomer subunit epsilon | 308 |  | 2 | 2 | 0 | 0 |  | 50.0 | 50.0 | 0.0 | 0.0 |  | 0.1563 | -1.1640 | 0.1125 |  | 6.748E-01 | 5.166E-01 | 8.264E-01 |
| 309 | Q9BTV4 | TMEM43 | Transmembrane protein 43 | 400 |  | 3 | 3 | 0 | 0 |  | 50.0 | 50.0 | 0.0 | 0.0 |  | 0.1563 | -1.5510 | -0.2746 |  | 7.588E-01 | 2.641E-01 | 5.442E-01 |
| 310 | Q15631 | TSN | Translin | 228 |  | 3 | 3 | 0 | 0 |  | 50.0 | 50.0 | 0.0 | 0.0 |  | 0.1563 | -1.5510 | -0.2746 |  | 7.588E-01 | 2.641E-01 | 5.442E-01 |
| 311 | Q15436 | SEC23A | Protein transport protein Sec23A | 765 |  | 3 | 3 | 0 | 0 |  | 50.0 | 50.0 | 0.0 | 0.0 |  | 0.1563 | -1.5510 | -0.2746 |  | 7.588E-01 | 2.641E-01 | 5.442E-01 |
| 312 | P08174 | CD55 | Complement decay-accelerating factor | 440 |  | 3 | 3 | 0 | 0 |  | 50.0 | 50.0 | 0.0 | 0.0 |  | 0.1563 | -1.5510 | -0.2746 |  | 7.588E-01 | 2.641E-01 | 5.442E-01 |
| 313 | O75533 | SF3B1 | Splicing factor 3B subunit 1 | 1304 |  | 3 | 3 | 0 | 0 |  | 50.0 | 50.0 | 0.0 | 0.0 |  | 0.1563 | -1.5510 | -0.2746 |  | 7.588E-01 | 2.641E-01 | 5.442E-01 |
| 314 | O60831 | PRAF2 | PRA1 family protein 2 | 178 |  | 3 | 3 | 0 | 0 |  | 50.0 | 50.0 | 0.0 | 0.0 |  | 0.1563 | -1.5510 | -0.2746 |  | 7.588E-01 | 2.641E-01 | 5.442E-01 |
| 315 | Q04941 | PLP2 | Proteolipid protein 2 | 152 |  | 4 | 4 | 0 | 0 |  | 50.0 | 50.0 | 0.0 | 0.0 |  | 0.1563 | -1.8560 | -0.5795 |  | 8.135E-01 | 1.365E-01 | 3.621E-01 |
| 316 | P62195 | PSMC5 | 26S protease regulatory subunit 8 | 406 |  | 4 | 4 | 0 | 0 |  | 50.0 | 50.0 | 0.0 | 0.0 |  | 0.1563 | -1.8560 | -0.5795 |  | 8.135E-01 | 1.365E-01 | 3.621E-01 |
| 317 | P60900 | PSMA6 | Proteasome subunit alpha type-6 | 246 |  | 4 | 4 | 0 | 0 |  | 50.0 | 50.0 | 0.0 | 0.0 |  | 0.1563 | -1.8560 | -0.5795 |  | 8.135E-01 | 1.365E-01 | 3.621E-01 |
| 318 | O60888 | CUTA | Protein CutA | 179 |  | 4 | 4 | 0 | 0 |  | 50.0 | 50.0 | 0.0 | 0.0 |  | 0.1563 | -1.8560 | -0.5795 |  | 8.135E-01 | 1.365E-01 | 3.621E-01 |
| 319 | Q13155 | AIMP2 | Aminoacyl tRNA synthase complex-interacting multifunctional protein 2 | 320 |  | 7 | 7 | 0 | 0 |  | 50.0 | 50.0 | 0.0 | 0.0 |  | 0.1563 | -2.5082 | -1.2318 |  | 9.116E-01 | 1.933E-02 | 1.093E-01 |
| 320 | O95336 | PGLS | 6-phosphogluconolactonase | 258 |  | 7 | 7 | 0 | 0 |  | 50.0 | 50.0 | 0.0 | 0.0 |  | 0.1563 | -2.5082 | -1.2318 |  | 9.116E-01 | 1.933E-02 | 1.093E-01 |
| 321 | P48643 | CCT5 | T-complex protein 1 subunit epsilon | 541 |  | 16 | 10 | 6 | 0 |  | 50.0 | 31.3 | 18.8 | 0.0 |  | -0.4607 | -1.0364 | -2.2964 |  | 4.405E-01 | 8.962E-02 | 3.215E-03 |
| 322 | P13861 | PRKAR2A | cAMP-dependent protein kinase type II-alpha regulatory subunit | 404 |  | 9 | 6 | 3 | 0 |  | 50.0 | 33.3 | 16.7 | 0.0 |  | -0.3434 | -1.0558 | -1.5450 |  | 7.222E-01 | 2.002E-01 | 4.970E-02 |
| 323 | Q16777 | HIST2H2AC | Histone H2A type 2-C | 129 |  | 117 | 119 | 0 | 0 |  | 49.6 | 50.4 | 0.0 | 0.0 |  | 0.1814 | -6.3562 | -5.0797 |  | 4.781E-01 | 4.033E-33 | 3.008E-20 |
| 324 | Q08380 | LGALS3BP | Galectin-3-binding protein | 585 |  | 40 | 23 | 18 | 0 |  | 49.4 | 28.4 | 22.2 | 0.0 |  | -0.6110 | -0.8860 | -3.5557 |  | 8.948E-02 | 1.719E-02 | 2.864E-07 |
| 325 | P63220 | RPS21 | 40S ribosomal protein S21 | 83 |  | 18 | 9 | 10 | 0 |  | 48.6 | 24.3 | 27.0 | 0.0 |  | -0.7534 | -0.5606 | -2.4548 |  | 1.815E-01 | 2.960E-01 | 1.475E-03 |
| 326 | P09668 | CTSH | Pro-cathepsin H | 335 |  | 14 | 4 | 11 | 0 |  | 48.3 | 13.8 | 37.9 | 0.0 |  | -1.3827 | -0.1014 | -2.1185 |  | 4.579E-02 | 8.972E-01 | 7.015E-03 |
| 327 | P20591 | MX1 | Interferon-induced GTP-binding protein Mx1 | 662 |  | 14 | 15 | 0 | 0 |  | 48.3 | 51.7 | 0.0 | 0.0 |  | 0.2481 | -3.3950 | -2.1185 |  | 8.218E-01 | 2.114E-04 | 7.015E-03 |
| 328 | P15880 | RPS2 | 40S ribosomal protein S2 | 293 |  | 13 | 5 | 9 | 0 |  | 48.1 | 18.5 | 33.3 | 0.0 |  | -1.0332 | -0.2608 | -2.0206 |  | 1.343E-01 | 6.989E-01 | 1.037E-02 |
| 329 | P31930 | UQCRC1 | Cytochrome b-c1 complex subunit 1, mitochondrial | 480 |  | 26 | 13 | 15 | 0 |  | 48.1 | 24.1 | 27.8 | 0.0 |  | -0.7797 | -0.5317 | -2.9567 |  | 9.313E-02 | 2.206E-01 | 6.569E-05 |
| 330 | P63313 | TMSB10 | Thymosin beta-10 | 44 |  | 20 | 12 | 10 | 0 |  | 47.6 | 28.6 | 23.8 | 0.0 |  | -0.5256 | -0.7034 | -2.5975 |  | 3.138E-01 | 1.732E-01 | 6.770E-04 |
| 331 | Q9H2U2 | PPA2 | Inorganic pyrophosphatase 2, mitochondrial | 334 |  | 10 | 6 | 5 | 0 |  | 47.6 | 28.6 | 23.8 | 0.0 |  | -0.4778 | -0.6336 | -1.6794 |  | 5.581E-01 | 4.062E-01 | 3.355E-02 |
| 332 | P49411 | TUFM | Elongation factor Tu, mitochondrial | 455 |  | 58 | 35 | 29 | 0 |  | 47.5 | 28.7 | 23.8 | 0.0 |  | -0.5537 | -0.7567 | -4.0792 |  | 5.837E-02 | 1.140E-02 | 2.655E-10 |
| 333 | P08134 | RHOC | Rho-related GTP-binding protein RhoC | 193 |  | 9 | 0 | 10 | 0 |  | 47.4 | 0.0 | 52.6 | 0.0 |  | -2.8799 | 0.3491 | -1.5450 |  | 4.776E-03 | 8.130E-01 | 4.970E-02 |
| 334 | P51649 | ALDH5A1 | Succinate-semialdehyde dehydrogenase, mitochondrial | 535 |  | 17 | 11 | 8 | 0 |  | 47.2 | 30.6 | 22.2 | 0.0 |  | -0.4191 | -0.7662 | -2.3778 |  | 4.672E-01 | 1.783E-01 | 2.177E-03 |
| 335 | Q14974 | KPNB1 | Importin subunit beta-1 | 876 |  | 25 | 14 | 14 | 0 |  | 47.2 | 26.4 | 26.4 | 0.0 |  | -0.6278 | -0.5694 | -2.9027 |  | 1.766E-01 | 2.026E-01 | 9.688E-05 |
| 336 | P18669 | PGAM1 | Phosphoglycerate mutase 1 | 254 |  | 33 | 19 | 18 | 0 |  | 47.1 | 27.1 | 25.7 | 0.0 |  | -0.6026 | -0.6173 | -3.2870 |  | 1.302E-01 | 1.120E-01 | 4.334E-06 |
| 337 | P83731 | RPL24 | 60S ribosomal protein L24 | 157 |  | 16 | 6 | 12 | 0 |  | 47.1 | 17.6 | 35.3 | 0.0 |  | -1.0948 | -0.1661 | -2.2964 |  | 7.931E-02 | 7.788E-01 | 3.215E-03 |
| 338 | P05091 | ALDH2 | Aldehyde dehydrogenase, mitochondrial | 517 |  | 88 | 44 | 36 | 19 |  | 47.1 | 23.5 | 19.3 | 10.2 |  | -0.8260 | -1.0487 | -0.6509 |  | 9.466E-04 | 4.912E-05 | 1.219E-03 |
| 339 | P14550 | AKR1A1 | Alcohol dehydrogenase [NADP(+)] | 325 |  | 62 | 26 | 44 | 0 |  | 47.0 | 19.7 | 33.3 | 0.0 |  | -1.0605 | -0.2691 | -4.1737 |  | 6.742E-04 | 2.718E-01 | 5.625E-11 |
| 340 | P06744 | GPI | Glucose-6-phosphate isomerase | 558 |  | 80 | 50 | 41 | 0 |  | 46.8 | 29.2 | 24.0 | 0.0 |  | -0.5100 | -0.7307 | -4.5361 |  | 3.730E-02 | 3.772E-03 | 5.211E-14 |
| 341 | P36578 | RPL4 | 60S ribosomal protein L4 | 427 |  | 27 | 11 | 20 | 0 |  | 46.6 | 19.0 | 34.5 | 0.0 |  | -1.0501 | -0.1963 | -3.0087 |  | 2.685E-02 | 6.133E-01 | 4.454E-05 |
| 342 | P04075 | ALDOA | Fructose-bisphosphate aldolase A | 364 |  | 72 | 49 | 35 | 0 |  | 46.2 | 31.4 | 22.4 | 0.0 |  | -0.3885 | -0.8021 | -4.3860 |  | 1.214E-01 | 3.048E-03 | 1.162E-12 |
| 343 | O94760 | DDAH1 | N(G),N(G)-dimethylarginine dimethylaminohydrolase 1 | 182 |  | 6 | 0 | 7 | 0 |  | 46.2 | 0.0 | 53.8 | 0.0 |  | -2.3801 | 0.4012 | -1.0453 |  | 3.453E-02 | 8.449E-01 | 1.626E-01 |
| 344 | Q9NTK5 | OLA1 | Obg-like ATPase 1 | 396 |  | 6 | 7 | 0 | 0 |  | 46.2 | 53.8 | 0.0 | 0.0 |  | 0.3428 | -2.3217 | -1.0453 |  | 8.801E-01 | 3.698E-02 | 1.626E-01 |
| 345 | P0C0S5 | H2AFZ | Histone H2A.Z | 128 |  | 35 | 41 | 0 | 0 |  | 46.1 | 53.9 | 0.0 | 0.0 |  | 0.3779 | -4.6454 | -3.3690 |  | 3.477E-01 | 2.941E-10 | 1.994E-06 |
| 346 | O43399 | TPD52L2 | Tumor protein D54 | 206 |  | 17 | 11 | 9 | 0 |  | 45.9 | 29.7 | 24.3 | 0.0 |  | -0.4191 | -0.6180 | -2.3778 |  | 4.672E-01 | 2.683E-01 | 2.177E-03 |
| 347 | Q16836 | HADH | Hydroxyacyl-coenzyme A dehydrogenase, mitochondrial | 390 |  | 28 | 18 | 15 | 0 |  | 45.9 | 29.5 | 24.6 | 0.0 |  | -0.4478 | -0.6340 | -3.0590 |  | 2.941E-01 | 1.363E-01 | 3.020E-05 |
| 348 | P12277 | CKB | Creatine kinase B-type | 381 |  | 32 | 38 | 0 | 0 |  | 45.7 | 54.3 | 0.0 | 0.0 |  | 0.3963 | -4.5206 | -3.2442 |  | 3.429E-01 | 2.016E-09 | 6.389E-06 |
| 349 | P52272 | HNRNPM | Heterogeneous nuclear ribonucleoprotein M | 730 |  | 120 | 82 | 50 | 11 |  | 45.6 | 31.2 | 19.0 | 4.2 |  | -0.3879 | -1.0314 | -1.8214 |  | 4.312E-02 | 2.848E-06 | 1.685E-10 |
| 350 | P49755 | TMED10 | Transmembrane emp24 domain-containing protein 10 | 219 |  | 20 | 0 | 24 | 0 |  | 45.5 | 0.0 | 54.5 | 0.0 |  | -3.9324 | 0.4640 | -2.5975 |  | 3.535E-06 | 4.164E-01 | 6.770E-04 |
| 351 | P40939 | HADHA | Trifunctional enzyme subunit alpha, mitochondrial | 763 |  | 30 | 18 | 18 | 0 |  | 45.5 | 27.3 | 27.3 | 0.0 |  | -0.5433 | -0.4849 | -3.1545 |  | 1.911E-01 | 2.219E-01 | 1.389E-05 |
| 352 | P30085 | CMPK1 | UMP-CMP kinase | 228 |  | 15 | 15 | 3 | 0 |  | 45.5 | 45.5 | 9.1 | 0.0 |  | 0.1564 | -1.7209 | -2.2102 |  | 9.630E-01 | 1.367E-02 | 4.748E-03 |
| 353 | Q9UL25 | RAB21 | Ras-related protein Rab-21 | 225 |  | 9 | 4 | 7 | 0 |  | 45.0 | 20.0 | 35.0 | 0.0 |  | -0.8092 | -0.0985 | -1.5450 |  | 3.324E-01 | 9.735E-01 | 4.970E-02 |
| 354 | P30048 | PRDX3 | Thioredoxin-dependent peroxide reductase, mitochondrial | 256 |  | 18 | 13 | 9 | 0 |  | 45.0 | 32.5 | 22.5 | 0.0 |  | -0.2778 | -0.6950 | -2.4548 |  | 6.261E-01 | 2.033E-01 | 1.475E-03 |
| 355 | P38646 | HSPA9 | Stress-70 protein, mitochondrial | 679 |  | 70 | 52 | 30 | 4 |  | 44.9 | 33.3 | 19.2 | 2.6 |  | -0.2645 | -0.9765 | -2.2749 |  | 2.792E-01 | 6.230E-04 | 4.047E-08 |
| 356 | P27797 | CALR | Calreticulin | 417 |  | 48 | 44 | 15 | 0 |  | 44.9 | 41.1 | 14.0 | 0.0 |  | 0.0341 | -1.3869 | -3.8119 |  | 9.296E-01 | 2.070E-04 | 1.285E-08 |
| 357 | P02763 | ORM1 | Alpha-1-acid glycoprotein 1 | 201 |  | 13 | 10 | 6 | 0 |  | 44.8 | 34.5 | 20.7 | 0.0 |  | -0.1849 | -0.7606 | -2.0206 |  | 8.282E-01 | 2.483E-01 | 1.037E-02 |
| 358 | P53004 | BLVRA | Biliverdin reductase A | 296 |  | 42 | 28 | 24 | 0 |  | 44.7 | 29.8 | 25.5 | 0.0 |  | -0.4086 | -0.5626 | -3.6241 |  | 2.261E-01 | 9.536E-02 | 1.318E-07 |
| 359 | P17655 | CAPN2 | Calpain-2 catalytic subunit | 622 |  | 21 | 19 | 7 | 0 |  | 44.7 | 40.4 | 14.9 | 0.0 |  | 0.0204 | -1.2174 | -2.6639 |  | 9.153E-01 | 2.612E-02 | 4.588E-04 |
| 360 | P07237 | P4HB | Protein disulfide-isomerase | 508 |  | 111 | 73 | 65 | 0 |  | 44.6 | 29.3 | 26.1 | 0.0 |  | -0.4418 | -0.5482 | -5.0042 |  | 2.954E-02 | 6.916E-03 | 3.093E-19 |
| 361 | P36957 | DLST | Dihydrolipoyllysine-residue succinyltransferase component of 2-oxoglutarate dehydrogenase complex, mitochondrial | 453 |  | 8 | 3 | 7 | 0 |  | 44.4 | 16.7 | 38.9 | 0.0 |  | -0.9660 | 0.0496 | -1.3969 |  | 2.787E-01 | 8.335E-01 | 7.367E-02 |
| 362 | P30626 | SRI | Sorcin | 198 |  | 20 | 12 | 13 | 0 |  | 44.4 | 26.7 | 28.9 | 0.0 |  | -0.5256 | -0.3621 | -2.5975 |  | 3.138E-01 | 4.611E-01 | 6.770E-04 |
| 363 | P24666 | ACP1 | Low molecular weight phosphotyrosine protein phosphatase | 158 |  | 8 | 7 | 3 | 0 |  | 44.4 | 38.9 | 16.7 | 0.0 |  | -0.0088 | -0.9076 | -1.3969 |  | 8.713E-01 | 2.972E-01 | 7.367E-02 |
| 364 | P07203 | GPX1 | Glutathione peroxidase 1 | 203 |  | 8 | 7 | 3 | 0 |  | 44.4 | 38.9 | 16.7 | 0.0 |  | -0.0088 | -0.9076 | -1.3969 |  | 8.713E-01 | 2.972E-01 | 7.367E-02 |
| 365 | P50238 | CRIP1 | Cysteine-rich protein 1 | 77 |  | 4 | 5 | 0 | 0 |  | 44.4 | 55.6 | 0.0 | 0.0 |  | 0.4079 | -1.8560 | -0.5795 |  | 9.001E-01 | 1.365E-01 | 3.621E-01 |
| 366 | P14625 | HSP90B1 | Endoplasmin | 803 |  | 134 | 78 | 74 | 17 |  | 44.2 | 25.7 | 24.4 | 5.6 |  | -0.6177 | -0.6341 | -1.4038 |  | 1.369E-03 | 8.076E-04 | 3.509E-09 |
| 367 | O75396 | SEC22B | Vesicle-trafficking protein SEC22b | 215 |  | 19 | 11 | 13 | 0 |  | 44.2 | 25.6 | 30.2 | 0.0 |  | -0.5693 | -0.2925 | -2.5279 |  | 2.908E-01 | 5.620E-01 | 9.991E-04 |
| 368 | Q13724 | MOGS | Mannosyl-oligosaccharide glucosidase | 837 |  | 15 | 10 | 9 | 0 |  | 44.1 | 29.4 | 26.5 | 0.0 |  | -0.3745 | -0.4504 | -2.2102 |  | 5.530E-01 | 4.473E-01 | 4.748E-03 |
| 369 | P42766 | RPL35 | 60S ribosomal protein L35 | 123 |  | 11 | 5 | 9 | 0 |  | 44.0 | 20.0 | 36.0 | 0.0 |  | -0.8149 | -0.0425 | -1.8023 |  | 2.735E-01 | 9.849E-01 | 2.267E-02 |
| 370 | Q07020 | RPL18 | 60S ribosomal protein L18 | 188 |  | 17 | 9 | 13 | 0 |  | 43.6 | 23.1 | 33.3 | 0.0 |  | -0.6764 | -0.1424 | -2.3778 |  | 2.420E-01 | 8.015E-01 | 2.177E-03 |
| 371 | P60981 | DSTN | Destrin | 165 |  | 23 | 14 | 13 | 3 |  | 43.4 | 26.4 | 24.5 | 5.7 |  | -0.5133 | -0.5528 | -1.0222 |  | 2.857E-01 | 2.360E-01 | 2.804E-02 |
| 372 | P61604 | HSPE1 | 10 kDa heat shock protein, mitochondrial | 102 |  | 29 | 14 | 24 | 0 |  | 43.3 | 20.9 | 35.8 | 0.0 |  | -0.8326 | -0.0461 | -3.1076 |  | 5.957E-02 | 8.767E-01 | 2.048E-05 |
| 373 | P38117 | ETFB | Electron transfer flavoprotein subunit beta | 255 |  | 19 | 17 | 8 | 0 |  | 43.2 | 38.6 | 18.2 | 0.0 |  | 0.0063 | -0.9163 | -2.5279 |  | 9.329E-01 | 9.491E-02 | 9.991E-04 |
| 374 | P07602 | PSAP | Proactivator polypeptide | 524 |  | 22 | 22 | 7 | 0 |  | 43.1 | 43.1 | 13.7 | 0.0 |  | 0.1564 | -1.2809 | -2.7274 |  | 8.992E-01 | 1.777E-02 | 3.110E-04 |
| 375 | Q9P2E9 | RRBP1 | Ribosome-binding protein 1 | 977 |  | 84 | 74 | 37 | 0 |  | 43.1 | 37.9 | 19.0 | 0.0 |  | -0.0238 | -0.9441 | -4.6056 |  | 8.485E-01 | 2.610E-04 | 1.103E-14 |
| 376 | Q15149 | PLEC | Plectin | 4574 |  | 266 | 154 | 148 | 50 |  | 43.0 | 24.9 | 23.9 | 8.1 |  | -0.6331 | -0.6315 | -0.8991 |  | 3.892E-06 | 2.421E-06 | 5.799E-11 |
| 377 | P61247 | RPS3A | 40S ribosomal protein S3a | 264 |  | 9 | 5 | 7 | 0 |  | 42.9 | 23.8 | 33.3 | 0.0 |  | -0.5576 | -0.0985 | -1.5450 |  | 5.162E-01 | 9.735E-01 | 4.970E-02 |
| 378 | Q86UE4 | MTDH | Protein LYRIC | 582 |  | 6 | 4 | 4 | 0 |  | 42.9 | 28.6 | 28.6 | 0.0 |  | -0.3095 | -0.2511 | -1.0453 |  | 8.537E-01 | 8.846E-01 | 1.626E-01 |
| 379 | Q9NZL9 | MAT2B | Methionine adenosyltransferase 2 subunit beta | 323 |  | 6 | 5 | 3 | 0 |  | 42.9 | 35.7 | 21.4 | 0.0 |  | -0.0579 | -0.5560 | -1.0453 |  | 8.897E-01 | 6.117E-01 | 1.626E-01 |
| 380 | O95219 | SNX4 | Sorting nexin-4 | 450 |  | 3 | 3 | 1 | 0 |  | 42.9 | 42.9 | 14.3 | 0.0 |  | 0.1563 | -0.7030 | -0.2746 |  | 7.588E-01 | 6.929E-01 | 5.442E-01 |
| 381 | Q92688 | ANP32B | Acidic leucine-rich nuclear phosphoprotein 32 family member B | 251 |  | 24 | 32 | 0 | 0 |  | 42.9 | 57.1 | 0.0 | 0.0 |  | 0.5541 | -4.1231 | -2.8466 |  | 2.113E-01 | 3.424E-07 | 1.429E-04 |
| 382 | Q02818 | NUCB1 | Nucleobindin-1 | 461 |  | 18 | 24 | 0 | 0 |  | 42.9 | 57.1 | 0.0 | 0.0 |  | 0.5482 | -3.7313 | -2.4548 |  | 2.966E-01 | 1.615E-05 | 1.475E-03 |
| 383 | O94832 | MYO1D | Unconventional myosin-Id | 1006 |  | 6 | 8 | 0 | 0 |  | 42.9 | 57.1 | 0.0 | 0.0 |  | 0.5079 | -2.3217 | -1.0453 |  | 6.716E-01 | 3.698E-02 | 1.626E-01 |
| 384 | Q07666 | KHDRBS1 | KH domain-containing, RNA-binding, signal transduction-associated protein 1 | 443 |  | 3 | 4 | 0 | 0 |  | 42.9 | 57.1 | 0.0 | 0.0 |  | 0.4612 | -1.5510 | -0.2746 |  | 9.119E-01 | 2.641E-01 | 5.442E-01 |
| 385 | P19971 | TYMP | Thymidine phosphorylase | 482 |  | 21 | 20 | 8 | 0 |  | 42.9 | 40.8 | 16.3 | 0.0 |  | 0.0900 | -1.0523 | -2.6639 |  | 9.643E-01 | 4.820E-02 | 4.588E-04 |
| 386 | P61981 | YWHAG | 14-3-3 protein gamma | 247 |  | 94 | 64 | 62 | 0 |  | 42.7 | 29.1 | 28.2 | 0.0 |  | -0.3908 | -0.3773 | -4.7663 |  | 7.302E-02 | 6.900E-02 | 2.275E-16 |
| 387 | Q04760 | GLO1 | Lactoylglutathione lyase | 184 |  | 29 | 29 | 10 | 0 |  | 42.6 | 42.6 | 14.7 | 0.0 |  | 0.1565 | -1.2134 | -3.1076 |  | 8.515E-01 | 9.129E-03 | 2.048E-05 |
| 388 | P52566 | ARHGDIB | Rho GDP-dissociation inhibitor 2 | 201 |  | 26 | 11 | 18 | 6 |  | 42.6 | 18.0 | 29.5 | 9.8 |  | -0.9980 | -0.2870 | -0.4196 |  | 3.748E-02 | 4.846E-01 | 1.372E-01 |
| 389 | Q15181 | PPA1 | Inorganic pyrophosphatase | 289 |  | 14 | 11 | 8 | 0 |  | 42.4 | 33.3 | 24.2 | 0.0 |  | -0.1598 | -0.5069 | -2.1185 |  | 8.483E-01 | 4.135E-01 | 7.015E-03 |
| 390 | Q14697 | GANAB | Neutral alpha-glucosidase AB | 944 |  | 89 | 61 | 60 | 0 |  | 42.4 | 29.0 | 28.6 | 0.0 |  | -0.3808 | -0.3457 | -4.6882 |  | 8.862E-02 | 1.003E-01 | 1.585E-15 |
| 391 | P51858 | HDGF | Hepatoma-derived growth factor | 240 |  | 22 | 21 | 9 | 0 |  | 42.3 | 40.4 | 17.3 | 0.0 |  | 0.0929 | -0.9676 | -2.7274 |  | 9.753E-01 | 5.920E-02 | 3.110E-04 |
| 392 | P54577 | YARS | Tyrosine--tRNA ligase, cytoplasmic | 528 |  | 11 | 9 | 6 | 0 |  | 42.3 | 34.6 | 23.1 | 0.0 |  | -0.1009 | -0.5423 | -1.8023 |  | 9.707E-01 | 4.518E-01 | 2.267E-02 |
| 393 | Q14764 | MVP | Major vault protein | 893 |  | 130 | 119 | 60 | 0 |  | 42.1 | 38.5 | 19.4 | 0.0 |  | 0.0302 | -0.8885 | -5.2310 |  | 9.793E-01 | 1.465E-05 | 1.927E-22 |
| 394 | P22314 | UBA1 | Ubiquitin-like modifier-activating enzyme 1 | 1058 |  | 98 | 75 | 56 | 4 |  | 42.1 | 32.2 | 24.0 | 1.7 |  | -0.2249 | -0.5811 | -2.7548 |  | 2.594E-01 | 7.822E-03 | 2.727E-12 |
| 395 | Q13011 | ECH1 | Delta(3,5)-Delta(2,4)-dienoyl-CoA isomerase, mitochondrial | 328 |  | 39 | 22 | 32 | 0 |  | 41.9 | 23.7 | 34.4 | 0.0 |  | -0.6363 | -0.0611 | -3.5202 |  | 8.234E-02 | 7.980E-01 | 4.222E-07 |
| 396 | O60437 | PPL | Periplakin | 1756 |  | 51 | 40 | 31 | 0 |  | 41.8 | 32.8 | 25.4 | 0.0 |  | -0.1851 | -0.4824 | -3.8974 |  | 5.147E-01 | 1.051E-01 | 4.013E-09 |
| 397 | P19338 | NCL | Nucleolin | 710 |  | 56 | 34 | 44 | 0 |  | 41.8 | 25.4 | 32.8 | 0.0 |  | -0.5445 | -0.1250 | -4.0295 |  | 6.711E-02 | 5.755E-01 | 5.768E-10 |
| 398 | P62701 | RPS4X | 40S ribosomal protein S4, X isoform | 263 |  | 38 | 26 | 27 | 0 |  | 41.8 | 28.6 | 29.7 | 0.0 |  | -0.3707 | -0.2602 | -3.4839 |  | 2.958E-01 | 4.196E-01 | 6.223E-07 |
| 399 | Q96FW1 | OTUB1 | Ubiquitin thioesterase OTUB1 | 271 |  | 5 | 0 | 7 | 0 |  | 41.7 | 0.0 | 58.3 | 0.0 |  | -2.1659 | 0.6154 | -0.8311 |  | 6.700E-02 | 6.335E-01 | 2.422E-01 |
| 400 | P55084 | HADHB | Trifunctional enzyme subunit beta, mitochondrial | 474 |  | 29 | 13 | 28 | 0 |  | 41.4 | 18.6 | 40.0 | 0.0 |  | -0.9306 | 0.1664 | -3.1076 |  | 3.812E-02 | 8.850E-01 | 2.048E-05 |
| 401 | Q9UBQ7 | GRHPR | Glyoxylate reductase/hydroxypyruvate reductase | 328 |  | 12 | 7 | 10 | 0 |  | 41.4 | 24.1 | 34.5 | 0.0 |  | -0.5275 | -0.0214 | -1.9156 |  | 4.594E-01 | 9.672E-01 | 1.533E-02 |
| 402 | P31040 | SDHA | Succinate dehydrogenase [ubiquinone] flavoprotein subunit, mitochondrial | 664 |  | 19 | 16 | 11 | 0 |  | 41.3 | 34.8 | 23.9 | 0.0 |  | -0.0751 | -0.5108 | -2.5279 |  | 9.276E-01 | 3.228E-01 | 9.991E-04 |
| 403 | P61158 | ACTR3 | Actin-related protein 3 | 418 |  | 26 | 20 | 17 | 0 |  | 41.3 | 31.7 | 27.0 | 0.0 |  | -0.2027 | -0.3641 | -2.9567 |  | 6.492E-01 | 3.846E-01 | 6.569E-05 |
| 404 | P35232 | PHB | Prohibitin | 272 |  | 63 | 47 | 41 | 2 |  | 41.2 | 30.7 | 26.8 | 1.3 |  | -0.2575 | -0.3910 | -2.8175 |  | 3.193E-01 | 1.293E-01 | 9.769E-09 |
| 405 | Q99798 | ACO2 | Aconitate hydratase, mitochondrial | 780 |  | 7 | 6 | 4 | 0 |  | 41.2 | 35.3 | 23.5 | 0.0 |  | -0.0301 | -0.4375 | -1.2318 |  | 8.801E-01 | 6.705E-01 | 1.093E-01 |
| 406 | Q15393 | SF3B3 | Splicing factor 3B subunit 3 | 1217 |  | 7 | 7 | 3 | 0 |  | 41.2 | 41.2 | 17.6 | 0.0 |  | 0.1563 | -0.7425 | -1.2318 |  | 9.116E-01 | 4.318E-01 | 1.093E-01 |
| 407 | Q8TD06 | AGR3 | Anterior gradient protein 3 homolog | 166 |  | 37 | 34 | 19 | 0 |  | 41.1 | 37.8 | 21.1 | 0.0 |  | 0.0385 | -0.7038 | -3.4466 |  | 9.084E-01 | 5.895E-02 | 9.174E-07 |
| 408 | P40926 | MDH2 | Malate dehydrogenase, mitochondrial | 338 |  | 63 | 41 | 40 | 10 |  | 40.9 | 26.6 | 26.0 | 6.5 |  | -0.4495 | -0.4256 | -1.0247 |  | 1.003E-01 | 1.031E-01 | 4.994E-04 |
| 409 | Q9BVC6 | TMEM109 | Transmembrane protein 109 | 243 |  | 9 | 6 | 7 | 0 |  | 40.9 | 27.3 | 31.8 | 0.0 |  | -0.3434 | -0.0985 | -1.5450 |  | 7.222E-01 | 9.735E-01 | 4.970E-02 |
| 410 | P40227 | CCT6A | T-complex protein 1 subunit zeta | 531 |  | 9 | 10 | 3 | 0 |  | 40.9 | 45.5 | 13.6 | 0.0 |  | 0.2907 | -1.0558 | -1.5450 |  | 8.553E-01 | 2.002E-01 | 4.970E-02 |
| 411 | P10809 | HSPD1 | 60 kDa heat shock protein, mitochondrial | 573 |  | 147 | 127 | 80 | 6 |  | 40.8 | 35.3 | 22.2 | 1.7 |  | -0.0531 | -0.6562 | -2.8707 |  | 6.471E-01 | 3.058E-04 | 5.874E-18 |
| 412 | P07741 | APRT | Adenine phosphoribosyltransferase | 180 |  | 20 | 16 | 13 | 0 |  | 40.8 | 32.7 | 26.5 | 0.0 |  | -0.1447 | -0.3621 | -2.5975 |  | 8.028E-01 | 4.611E-01 | 6.770E-04 |
| 413 | P27824 | CANX | Calnexin | 592 |  | 111 | 85 | 76 | 0 |  | 40.8 | 31.3 | 27.9 | 0.0 |  | -0.2248 | -0.3258 | -5.0042 |  | 2.278E-01 | 7.885E-02 | 3.093E-19 |
| 414 | Q13510 | ASAH1 | Acid ceramidase | 395 |  | 11 | 9 | 7 | 0 |  | 40.7 | 33.3 | 25.9 | 0.0 |  | -0.1009 | -0.3558 | -1.8023 |  | 9.707E-01 | 6.317E-01 | 2.267E-02 |
| 415 | P78371 | CCT2 | T-complex protein 1 subunit beta | 535 |  | 37 | 34 | 20 | 0 |  | 40.7 | 37.4 | 22.0 | 0.0 |  | 0.0385 | -0.6342 | -3.4466 |  | 9.084E-01 | 8.395E-02 | 9.174E-07 |
| 416 | Q99497 | PARK7 | Protein DJ-1 | 189 |  | 56 | 36 | 33 | 13 |  | 40.6 | 26.1 | 23.9 | 9.4 |  | -0.4647 | -0.5276 | -0.5164 |  | 1.118E-01 | 6.618E-02 | 1.980E-02 |
| 417 | Q13162 | PRDX4 | Peroxiredoxin-4 | 271 |  | 15 | 15 | 7 | 0 |  | 40.5 | 40.5 | 18.9 | 0.0 |  | 0.1564 | -0.7637 | -2.2102 |  | 9.630E-01 | 2.099E-01 | 4.748E-03 |
| 418 | Q9H299 | SH3BGRL3 | SH3 domain-binding glutamic acid-rich-like protein 3 | 93 |  | 15 | 14 | 8 | 0 |  | 40.5 | 37.8 | 21.6 | 0.0 |  | 0.0647 | -0.5986 | -2.2102 |  | 8.835E-01 | 3.172E-01 | 4.748E-03 |
| 419 | Q9BW30 | TPPP3 | Tubulin polymerization-promoting protein family member 3 | 176 |  | 17 | 25 | 0 | 0 |  | 40.5 | 59.5 | 0.0 | 0.0 |  | 0.6814 | -3.6543 | -2.3778 |  | 1.758E-01 | 3.071E-05 | 2.177E-03 |
| 420 | P62249 | RPS16 | 40S ribosomal protein S16 | 146 |  | 19 | 11 | 17 | 0 |  | 40.4 | 23.4 | 36.2 | 0.0 |  | -0.5693 | 0.0647 | -2.5279 |  | 2.908E-01 | 8.738E-01 | 9.991E-04 |
| 421 | P04632 | CAPNS1 | Calpain small subunit 1 | 268 |  | 60 | 53 | 36 | 0 |  | 40.3 | 35.6 | 24.2 | 0.0 |  | -0.0189 | -0.5039 | -4.1272 |  | 9.042E-01 | 6.691E-02 | 1.222E-10 |
| 422 | P29692 | EEF1D | Elongation factor 1-delta | 281 |  | 41 | 33 | 28 | 0 |  | 40.2 | 32.4 | 27.5 | 0.0 |  | -0.1468 | -0.3164 | -3.5903 |  | 6.489E-01 | 3.174E-01 | 1.943E-07 |
| 423 | P62826 | RAN | GTP-binding nuclear protein Ran | 216 |  | 28 | 24 | 18 | 0 |  | 40.0 | 34.3 | 25.7 | 0.0 |  | -0.0559 | -0.3893 | -3.0590 |  | 9.086E-01 | 3.344E-01 | 3.020E-05 |
| 424 | P52209 | PGD | 6-phosphogluconate dehydrogenase, decarboxylating | 483 |  | 40 | 33 | 23 | 4 |  | 40.0 | 33.0 | 23.0 | 4.0 |  | -0.1122 | -0.5525 | -1.4846 |  | 7.294E-01 | 1.096E-01 | 6.054E-04 |
| 425 | P06454 | PTMA | Prothymosin alpha | 111 |  | 20 | 19 | 11 | 0 |  | 40.0 | 38.0 | 22.0 | 0.0 |  | 0.0868 | -0.5804 | -2.5975 |  | 9.527E-01 | 2.521E-01 | 6.770E-04 |
| 426 | P51659 | HSD17B4 | Peroxisomal multifunctional enzyme type 2 | 736 |  | 24 | 16 | 9 | 11 |  | 40.0 | 26.7 | 15.0 | 18.3 |  | -0.3938 | -1.0868 | 0.4481 |  | 3.974E-01 | 2.994E-02 | 9.216E-01 |
| 427 | Q15056 | EIF4H | Eukaryotic translation initiation factor 4H | 228 |  | 4 | 0 | 6 | 0 |  | 40.0 | 0.0 | 60.0 | 0.0 |  | -1.9143 | 0.6805 | -0.5795 |  | 1.304E-01 | 6.255E-01 | 3.621E-01 |
| 428 | P40429 | RPL13A | 60S ribosomal protein L13a | 203 |  | 6 | 3 | 6 | 0 |  | 40.0 | 20.0 | 40.0 | 0.0 |  | -0.6144 | 0.2147 | -1.0453 |  | 5.860E-01 | 9.195E-01 | 1.626E-01 |
| 429 | Q16563 | SYPL1 | Synaptophysin-like protein 1 | 259 |  | 10 | 7 | 8 | 0 |  | 40.0 | 28.0 | 32.0 | 0.0 |  | -0.2913 | -0.0678 | -1.6794 |  | 7.539E-01 | 9.955E-01 | 3.355E-02 |
| 430 | P36776 | LONP1 | Lon protease homolog, mitochondrial | 959 |  | 4 | 3 | 3 | 0 |  | 40.0 | 30.0 | 30.0 | 0.0 |  | -0.1486 | -0.0902 | -0.5795 |  | 9.119E-01 | 8.858E-01 | 3.621E-01 |
| 431 | P09874 | PARP1 | Poly [ADP-ribose] polymerase 1 | 1014 |  | 4 | 3 | 3 | 0 |  | 40.0 | 30.0 | 30.0 | 0.0 |  | -0.1486 | -0.0902 | -0.5795 |  | 9.119E-01 | 8.858E-01 | 3.621E-01 |
| 432 | Q9UGT4 | SUSD2 | Sushi domain-containing protein 2 | 822 |  | 8 | 9 | 3 | 0 |  | 40.0 | 45.0 | 15.0 | 0.0 |  | 0.3045 | -0.9076 | -1.3969 |  | 8.631E-01 | 2.972E-01 | 7.367E-02 |
| 433 | Q9UM54 | MYO6 | Unconventional myosin-VI | 1285 |  | 6 | 8 | 1 | 0 |  | 40.0 | 53.3 | 6.7 | 0.0 |  | 0.5079 | -1.4737 | -1.0453 |  | 6.716E-01 | 1.556E-01 | 1.626E-01 |
| 434 | P07814 | EPRS | Bifunctional glutamate/proline--tRNA ligase | 1512 |  | 6 | 9 | 0 | 0 |  | 40.0 | 60.0 | 0.0 | 0.0 |  | 0.6561 | -2.3217 | -1.0453 |  | 4.971E-01 | 3.698E-02 | 1.626E-01 |
| 435 | Q92817 | EVPL | Envoplakin | 2033 |  | 4 | 6 | 0 | 0 |  | 40.0 | 60.0 | 0.0 | 0.0 |  | 0.6221 | -1.8560 | -0.5795 |  | 6.536E-01 | 1.365E-01 | 3.621E-01 |
| 436 | O00754 | MAN2B1 | Lysosomal alpha-mannosidase | 1010 |  | 4 | 6 | 0 | 0 |  | 40.0 | 60.0 | 0.0 | 0.0 |  | 0.6221 | -1.8560 | -0.5795 |  | 6.536E-01 | 1.365E-01 | 3.621E-01 |
| 437 | Q9UNM6 | PSMD13 | 26S proteasome non-ATPase regulatory subunit 13 | 376 |  | 2 | 3 | 0 | 0 |  | 40.0 | 60.0 | 0.0 | 0.0 |  | 0.5434 | -1.1640 | 0.1125 |  | 9.255E-01 | 5.166E-01 | 8.264E-01 |
| 438 | P39060 | COL18A1 | Collagen alpha-1(XVIII) chain | 1336 |  | 2 | 3 | 0 | 0 |  | 40.0 | 60.0 | 0.0 | 0.0 |  | 0.5434 | -1.1640 | 0.1125 |  | 9.255E-01 | 5.166E-01 | 8.264E-01 |
| 439 | O94886 | TMEM63A | Transmembrane protein 63A | 807 |  | 2 | 3 | 0 | 0 |  | 40.0 | 60.0 | 0.0 | 0.0 |  | 0.5434 | -1.1640 | 0.1125 |  | 9.255E-01 | 5.166E-01 | 8.264E-01 |
| 440 | O75643 | SNRNP200 | U5 small nuclear ribonucleoprotein 200 kDa helicase | 2136 |  | 2 | 3 | 0 | 0 |  | 40.0 | 60.0 | 0.0 | 0.0 |  | 0.5434 | -1.1640 | 0.1125 |  | 9.255E-01 | 5.166E-01 | 8.264E-01 |
| 441 | O15260 | SURF4 | Surfeit locus protein 4 | 269 |  | 2 | 3 | 0 | 0 |  | 40.0 | 60.0 | 0.0 | 0.0 |  | 0.5434 | -1.1640 | 0.1125 |  | 9.255E-01 | 5.166E-01 | 8.264E-01 |
| 442 | Q15366 | PCBP2 | Poly(rC)-binding protein 2 | 365 |  | 17 | 16 | 10 | 0 |  | 39.5 | 37.2 | 23.3 | 0.0 |  | 0.0750 | -0.4836 | -2.3778 |  | 9.138E-01 | 3.792E-01 | 2.177E-03 |
| 443 | Q9BVK6 | TMED9 | Transmembrane emp24 domain-containing protein 9 | 235 |  | 17 | 11 | 15 | 0 |  | 39.5 | 25.6 | 34.9 | 0.0 |  | -0.4191 | 0.0472 | -2.3778 |  | 4.672E-01 | 8.965E-01 | 2.177E-03 |
| 444 | P19367 | HK1 | Hexokinase-1 | 917 |  | 15 | 9 | 14 | 0 |  | 39.5 | 23.7 | 36.8 | 0.0 |  | -0.5088 | 0.1231 | -2.2102 |  | 4.119E-01 | 9.366E-01 | 4.748E-03 |
| 445 | P12235 | SLC25A4 | ADP/ATP translocase 1 | 298 |  | 35 | 28 | 26 | 0 |  | 39.3 | 31.5 | 29.2 | 0.0 |  | -0.1535 | -0.1973 | -3.3690 |  | 6.707E-01 | 5.463E-01 | 1.994E-06 |
| 446 | Q08211 | DHX9 | ATP-dependent RNA helicase A | 1270 |  | 57 | 57 | 31 | 0 |  | 39.3 | 39.3 | 21.4 | 0.0 |  | 0.1567 | -0.6396 | -4.0546 |  | 7.239E-01 | 2.921E-02 | 3.913E-10 |
| 447 | P14618 | PKM2 | Pyruvate kinase isozymes M1/M2 | 531 |  | 244 | 170 | 160 | 47 |  | 39.3 | 27.4 | 25.8 | 7.6 |  | -0.3652 | -0.3938 | -0.8613 |  | 5.768E-03 | 2.131E-03 | 8.360E-10 |
| 448 | P63244 | GNB2L1 | Guanine nucleotide-binding protein subunit beta-2-like 1 | 317 |  | 22 | 19 | 15 | 0 |  | 39.3 | 33.9 | 26.8 | 0.0 |  | -0.0431 | -0.3024 | -2.7274 |  | 9.643E-01 | 5.094E-01 | 3.110E-04 |
| 449 | P60842 | EIF4A1 | Eukaryotic initiation factor 4A-I | 406 |  | 31 | 20 | 26 | 2 |  | 39.2 | 25.3 | 32.9 | 2.5 |  | -0.4461 | -0.0284 | -1.8212 |  | 2.679E-01 | 9.039E-01 | 6.657E-04 |
| 450 | P27695 | APEX1 | DNA-(apurinic or apyrimidinic site) lyase | 318 |  | 29 | 23 | 22 | 0 |  | 39.2 | 31.1 | 29.7 | 0.0 |  | -0.1629 | -0.1652 | -3.1076 |  | 6.944E-01 | 6.494E-01 | 2.048E-05 |
| 451 | P23396 | RPS3 | 40S ribosomal protein S3 | 243 |  | 36 | 26 | 30 | 0 |  | 39.1 | 28.3 | 32.6 | 0.0 |  | -0.2951 | -0.0388 | -3.4083 |  | 4.139E-01 | 8.607E-01 | 1.352E-06 |
| 452 | P84098 | RPL19 | 60S ribosomal protein L19 | 196 |  | 9 | 9 | 5 | 0 |  | 39.1 | 39.1 | 21.7 | 0.0 |  | 0.1564 | -0.4992 | -1.5450 |  | 9.534E-01 | 5.465E-01 | 4.970E-02 |
| 453 | HIP000046066 | IGHM | Immunoglobulin C1-set domain containing protein GN | 472 |  | 41 | 0 | 0 | 64 |  | 39.0 | 0.0 | 0.0 | 61.0 |  | -4.9251 | -4.8668 | 2.1266 |  | 3.862E-12 | 6.260E-12 | 2.309E-09 |
| 454 | P06733 | ENO1 | Alpha-enolase | 434 |  | 265 | 203 | 158 | 53 |  | 39.0 | 29.9 | 23.3 | 7.8 |  | -0.2285 | -0.5318 | -0.8110 |  | 5.361E-02 | 4.302E-05 | 5.710E-10 |
| 455 | P07195 | LDHB | L-lactate dehydrogenase B chain | 334 |  | 76 | 39 | 65 | 15 |  | 39.0 | 20.0 | 33.3 | 7.7 |  | -0.7862 | -0.0070 | -0.7600 |  | 3.233E-03 | 8.423E-01 | 1.123E-03 |
| 456 | O00299_1 | CLIC1 | Chloride intracellular channel protein 1 | 241 |  | 30 | 28 | 19 | 0 |  | 39.0 | 36.4 | 24.7 | 0.0 |  | 0.0609 | -0.4117 | -3.1545 |  | 9.397E-01 | 2.910E-01 | 1.389E-05 |
| 457 | P38159 | RBMX | RNA-binding motif protein, X chromosome | 391 |  | 28 | 24 | 20 | 0 |  | 38.9 | 33.3 | 27.8 | 0.0 |  | -0.0559 | -0.2466 | -3.0590 |  | 9.086E-01 | 5.238E-01 | 3.020E-05 |
| 458 | P62917 | RPL8 | 60S ribosomal protein L8 | 257 |  | 21 | 12 | 12 | 9 |  | 38.9 | 22.2 | 22.2 | 16.7 |  | -0.5920 | -0.5336 | 0.3732 |  | 2.456E-01 | 2.756E-01 | 9.613E-01 |
| 459 | Q02878 | RPL6 | 60S ribosomal protein L6 | 288 |  | 26 | 19 | 21 | 1 |  | 38.8 | 28.4 | 31.3 | 1.5 |  | -0.2723 | -0.0779 | -2.1085 |  | 5.379E-01 | 8.319E-01 | 6.741E-04 |
| 460 | P49591 | SARS | Serine--tRNA ligase, cytoplasmic | 514 |  | 10 | 5 | 11 | 0 |  | 38.5 | 19.2 | 42.3 | 0.0 |  | -0.6920 | 0.3377 | -1.6794 |  | 3.797E-01 | 8.036E-01 | 3.355E-02 |
| 461 | P18124 | RPL7 | 60S ribosomal protein L7 | 248 |  | 10 | 6 | 10 | 0 |  | 38.5 | 23.1 | 38.5 | 0.0 |  | -0.4778 | 0.2148 | -1.6794 |  | 5.581E-01 | 9.849E-01 | 3.355E-02 |
| 462 | P62328 | TMSB4X | Thymosin beta-4 | 44 |  | 43 | 30 | 22 | 17 |  | 38.4 | 26.8 | 19.6 | 15.2 |  | -0.3461 | -0.7148 | 0.2136 |  | 2.927E-01 | 3.854E-02 | 6.257E-01 |
| 463 | P30101 | PDIA3 | Protein disulfide-isomerase A3 | 505 |  | 145 | 105 | 105 | 23 |  | 38.4 | 27.8 | 27.8 | 6.1 |  | -0.3064 | -0.2477 | -1.1061 |  | 6.926E-02 | 1.052E-01 | 6.043E-08 |
| 464 | Q5SSJ5 | HP1BP3 | Heterochromatin protein 1-binding protein 3 | 553 |  | 23 | 21 | 16 | 0 |  | 38.3 | 35.0 | 26.7 | 0.0 |  | 0.0321 | -0.2770 | -2.7882 |  | 8.992E-01 | 5.320E-01 | 2.108E-04 |
| 465 | O60506 | SYNCRIP | Heterogeneous nuclear ribonucleoprotein Q | 623 |  | 41 | 35 | 31 | 0 |  | 38.3 | 32.7 | 29.0 | 0.0 |  | -0.0648 | -0.1753 | -3.5903 |  | 8.340E-01 | 5.465E-01 | 1.943E-07 |
| 466 | P31946 | YWHAB | 14-3-3 protein beta/alpha | 246 |  | 95 | 86 | 67 | 0 |  | 38.3 | 34.7 | 27.0 | 0.0 |  | 0.0147 | -0.2823 | -4.7814 |  | 9.742E-01 | 1.498E-01 | 1.543E-16 |
| 467 | Q14011 | CIRBP | Cold-inducible RNA-binding protein | 172 |  | 13 | 9 | 12 | 0 |  | 38.2 | 26.5 | 35.3 | 0.0 |  | -0.3192 | 0.1098 | -2.0206 |  | 6.564E-01 | 8.972E-01 | 1.037E-02 |
| 468 | P62081 | RPS7 | 40S ribosomal protein S7 | 194 |  | 13 | 14 | 7 | 0 |  | 38.2 | 41.2 | 20.6 | 0.0 |  | 0.2543 | -0.5741 | -2.0206 |  | 8.279E-01 | 3.772E-01 | 1.037E-02 |
| 469 | P18754_1 | RCC1 | Regulator of chromosome condensation | 421 |  | 26 | 32 | 10 | 0 |  | 38.2 | 47.1 | 14.7 | 0.0 |  | 0.4440 | -1.0625 | -2.9567 |  | 3.291E-01 | 2.645E-02 | 6.569E-05 |
| 470 | P60174 | TPI1 | Triosephosphate isomerase | 284 |  | 90 | 58 | 65 | 23 |  | 38.1 | 24.6 | 27.5 | 9.7 |  | -0.4683 | -0.2481 | -0.4222 |  | 3.995E-02 | 2.077E-01 | 7.465E-03 |
| 471 | P51149 | RAB7A | Ras-related protein Rab-7a | 207 |  | 16 | 15 | 11 | 0 |  | 38.1 | 35.7 | 26.2 | 0.0 |  | 0.0702 | -0.2793 | -2.2964 |  | 8.992E-01 | 6.240E-01 | 3.215E-03 |
| 472 | P09622 | DLD | Dihydrolipoyl dehydrogenase, mitochondrial | 509 |  | 8 | 0 | 13 | 0 |  | 38.1 | 0.0 | 61.9 | 0.0 |  | -2.7317 | 0.8385 | -1.3969 |  | 9.224E-03 | 2.611E-01 | 7.367E-02 |
| 473 | Q07955 | SFRS1 | Serine/arginine-rich splicing factor 1 | 248 |  | 16 | 13 | 13 | 0 |  | 38.1 | 31.0 | 31.0 | 0.0 |  | -0.1194 | -0.0610 | -2.2964 |  | 8.835E-01 | 9.366E-01 | 3.215E-03 |
| 474 | O00571 | DDX3X | ATP-dependent RNA helicase DDX3X | 662 |  | 11 | 18 | 0 | 0 |  | 37.9 | 62.1 | 0.0 | 0.0 |  | 0.8089 | -3.0788 | -1.8023 |  | 1.792E-01 | 1.459E-03 | 2.267E-02 |
| 475 | Q9UHQ9 | CYB5R1 | NADH-cytochrome b5 reductase 1 | 305 |  | 14 | 13 | 10 | 0 |  | 37.8 | 35.1 | 27.0 | 0.0 |  | 0.0585 | -0.2244 | -2.1185 |  | 8.666E-01 | 7.279E-01 | 7.015E-03 |
| 476 | P39656 | DDOST | Dolichyl-diphosphooligosaccharide--protein glycosyltransferase 48 kDa subunit | 456 |  | 20 | 19 | 14 | 0 |  | 37.7 | 35.8 | 26.4 | 0.0 |  | 0.0868 | -0.2642 | -2.5975 |  | 9.527E-01 | 5.867E-01 | 6.770E-04 |
| 477 | P04080 | CSTB | Cystatin-B | 98 |  | 20 | 17 | 14 | 2 |  | 37.7 | 32.1 | 26.4 | 3.8 |  | -0.0633 | -0.2642 | -1.2187 |  | 9.405E-01 | 5.867E-01 | 2.265E-02 |
| 478 | P16152 | CBR1 | Carbonyl reductase [NADPH] 1 | 277 |  | 53 | 30 | 44 | 14 |  | 37.6 | 21.3 | 31.2 | 9.9 |  | -0.6407 | -0.0472 | -0.3405 |  | 4.010E-02 | 7.801E-01 | 5.772E-02 |
| 479 | Q9UL46 | PSME2 | Proteasome activator complex subunit 2 | 239 |  | 36 | 29 | 25 | 6 |  | 37.5 | 30.2 | 26.0 | 6.3 |  | -0.1442 | -0.2907 | -0.8712 |  | 6.831E-01 | 3.891E-01 | 1.385E-02 |
| 480 | P22307 | SCP2 | Non-specific lipid-transfer protein | 547 |  | 6 | 4 | 6 | 0 |  | 37.5 | 25.0 | 37.5 | 0.0 |  | -0.3095 | 0.2147 | -1.0453 |  | 8.537E-01 | 9.195E-01 | 1.626E-01 |
| 481 | P62424 | RPL7A | 60S ribosomal protein L7a | 266 |  | 6 | 5 | 5 | 0 |  | 37.5 | 31.3 | 31.3 | 0.0 |  | -0.0579 | 0.0005 | -1.0453 |  | 8.897E-01 | 8.572E-01 | 1.626E-01 |
| 482 | P68402 | PAFAH1B2 | Platelet-activating factor acetylhydrolase IB subunit beta | 229 |  | 12 | 11 | 9 | 0 |  | 37.5 | 34.4 | 28.1 | 0.0 |  | 0.0431 | -0.1558 | -1.9156 |  | 8.410E-01 | 8.505E-01 | 1.533E-02 |
| 483 | P20339 | RAB5A | Ras-related protein Rab-5A | 215 |  | 3 | 3 | 2 | 0 |  | 37.5 | 37.5 | 25.0 | 0.0 |  | 0.1563 | -0.1724 | -0.2746 |  | 7.588E-01 | 9.034E-01 | 5.442E-01 |
| 484 | P55854 | SUMO3 | Small ubiquitin-related modifier 3 | 103 |  | 9 | 9 | 6 | 0 |  | 37.5 | 37.5 | 25.0 | 0.0 |  | 0.1564 | -0.2850 | -1.5450 |  | 9.534E-01 | 7.584E-01 | 4.970E-02 |
| 485 | Q14204 | DYNC1H1 | Cytoplasmic dynein 1 heavy chain 1 | 4646 |  | 39 | 41 | 24 | 0 |  | 37.5 | 39.4 | 23.1 | 0.0 |  | 0.2267 | -0.4587 | -3.5202 |  | 6.268E-01 | 1.791E-01 | 4.222E-07 |
| 486 | Q92804 | TAF15 | TATA-binding protein-associated factor 2N | 592 |  | 18 | 19 | 11 | 0 |  | 37.5 | 39.6 | 22.9 | 0.0 |  | 0.2295 | -0.4377 | -2.4548 |  | 7.991E-01 | 4.079E-01 | 1.475E-03 |
| 487 | Q9BRA2 | TXNDC17 | Thioredoxin domain-containing protein 17 | 123 |  | 6 | 7 | 3 | 0 |  | 37.5 | 43.8 | 18.8 | 0.0 |  | 0.3428 | -0.5560 | -1.0453 |  | 8.801E-01 | 6.117E-01 | 1.626E-01 |
| 488 | P40616 | ARL1 | ADP-ribosylation factor-like protein 1 | 181 |  | 3 | 5 | 0 | 0 |  | 37.5 | 62.5 | 0.0 | 0.0 |  | 0.7128 | -1.5510 | -0.2746 |  | 6.368E-01 | 2.641E-01 | 5.442E-01 |
| 489 | Q13630 | TSTA3 | GDP-L-fucose synthase | 321 |  | 9 | 8 | 7 | 0 |  | 37.5 | 33.3 | 29.2 | 0.0 |  | 0.0082 | -0.0985 | -1.5450 |  | 8.631E-01 | 9.735E-01 | 4.970E-02 |
| 490 | P53618 | COPB1 | Coatomer subunit beta | 953 |  | 9 | 8 | 7 | 0 |  | 37.5 | 33.3 | 29.2 | 0.0 |  | 0.0082 | -0.0985 | -1.5450 |  | 8.631E-01 | 9.735E-01 | 4.970E-02 |
| 491 | P49368 | CCT3 | T-complex protein 1 subunit gamma | 545 |  | 25 | 22 | 20 | 0 |  | 37.3 | 32.8 | 29.9 | 0.0 |  | -0.0188 | -0.0903 | -2.9027 |  | 9.959E-01 | 8.156E-01 | 9.688E-05 |
| 492 | P27348 | YWHAQ | 14-3-3 protein theta | 245 |  | 74 | 67 | 58 | 0 |  | 37.2 | 33.7 | 29.1 | 0.0 |  | 0.0155 | -0.1306 | -4.4250 |  | 9.929E-01 | 4.930E-01 | 5.346E-13 |
| 493 | Q15084 | PDIA6 | Protein disulfide-isomerase A6 | 440 |  | 80 | 64 | 69 | 3 |  | 37.0 | 29.6 | 31.9 | 1.4 |  | -0.1606 | 0.0048 | -2.7700 |  | 4.526E-01 | 8.745E-01 | 1.949E-10 |
| 494 | P62857 | RPS28 | 40S ribosomal protein S28 | 69 |  | 20 | 16 | 18 | 0 |  | 37.0 | 29.6 | 33.3 | 0.0 |  | -0.1447 | 0.0721 | -2.5975 |  | 8.028E-01 | 8.785E-01 | 6.770E-04 |
| 495 | Q6NUK1 | SLC25A24 | Calcium-binding mitochondrial carrier protein SCaMC-1 | 477 |  | 10 | 8 | 9 | 0 |  | 37.0 | 29.6 | 33.3 | 0.0 |  | -0.1262 | 0.0804 | -1.6794 |  | 9.534E-01 | 8.234E-01 | 3.355E-02 |
| 496 | P13073 | COX4I1 | Cytochrome c oxidase subunit 4 isoform 1, mitochondrial | 169 |  | 10 | 9 | 8 | 0 |  | 37.0 | 33.3 | 29.6 | 0.0 |  | 0.0220 | -0.0678 | -1.6794 |  | 8.553E-01 | 9.955E-01 | 3.355E-02 |
| 497 | P30740 | SERPINB1 | Leukocyte elastase inhibitor | 379 |  | 27 | 32 | 14 | 0 |  | 37.0 | 43.8 | 19.2 | 0.0 |  | 0.3919 | -0.6754 | -3.0087 |  | 3.994E-01 | 1.226E-01 | 4.454E-05 |
| 498 | O75915 | ARL6IP5 | PRA1 family protein 3 | 188 |  | 17 | 14 | 15 | 0 |  | 37.0 | 30.4 | 32.6 | 0.0 |  | -0.1029 | 0.0472 | -2.3778 |  | 8.992E-01 | 8.965E-01 | 2.177E-03 |
| 499 | P50454 | SERPINH1 | Serpin H1 | 418 |  | 79 | 81 | 54 | 0 |  | 36.9 | 37.9 | 25.2 | 0.0 |  | 0.1925 | -0.3248 | -4.5181 |  | 5.425E-01 | 1.428E-01 | 7.681E-14 |
| 500 | P30049 | ATP5D | ATP synthase subunit delta, mitochondrial | 168 |  | 14 | 13 | 11 | 0 |  | 36.8 | 34.2 | 28.9 | 0.0 |  | 0.0585 | -0.1014 | -2.1185 |  | 8.666E-01 | 8.972E-01 | 7.015E-03 |
| 501 | P07858 | CTSB | Cathepsin B | 339 |  | 14 | 8 | 16 | 0 |  | 36.8 | 21.1 | 42.1 | 0.0 |  | -0.5653 | 0.3927 | -2.1185 |  | 3.812E-01 | 6.313E-01 | 7.015E-03 |
| 502 | P67812 | SEC11A | Signal peptidase complex catalytic subunit SEC11A | 179 |  | 7 | 4 | 8 | 0 |  | 36.8 | 21.1 | 42.1 | 0.0 |  | -0.4959 | 0.3799 | -1.2318 |  | 6.408E-01 | 8.335E-01 | 1.093E-01 |
| 503 | Q9NSE4 | IARS2 | Isoleucine--tRNA ligase, mitochondrial | 1012 |  | 7 | 7 | 5 | 0 |  | 36.8 | 36.8 | 26.3 | 0.0 |  | 0.1563 | -0.1859 | -1.2318 |  | 9.116E-01 | 9.195E-01 | 1.093E-01 |
| 504 | P05387 | RPLP2 | 60S acidic ribosomal protein P2 | 115 |  | 14 | 13 | 10 | 1 |  | 36.8 | 34.2 | 26.3 | 2.6 |  | 0.0585 | -0.2244 | -1.2704 |  | 8.666E-01 | 7.279E-01 | 4.278E-02 |
| 505 | P05141 | SLC25A5 | ADP/ATP translocase 2 | 298 |  | 44 | 35 | 41 | 0 |  | 36.7 | 29.2 | 34.2 | 0.0 |  | -0.1640 | 0.1159 | -3.6894 |  | 5.961E-01 | 9.473E-01 | 6.066E-08 |
| 506 | P23246 | SFPQ | Splicing factor, proline- and glutamine-rich | 707 |  | 67 | 71 | 46 | 0 |  | 36.4 | 38.6 | 25.0 | 0.0 |  | 0.2392 | -0.3167 | -4.2837 |  | 4.546E-01 | 1.891E-01 | 8.084E-12 |
| 507 | P11021 | HSPA5 | 78 kDa glucose-regulated protein | 654 |  | 180 | 154 | 143 | 18 |  | 36.4 | 31.1 | 28.9 | 3.6 |  | -0.0676 | -0.1156 | -1.7518 |  | 5.439E-01 | 3.006E-01 | 2.290E-14 |
| 508 | P49721 | PSMB2 | Proteasome subunit beta type-2 | 201 |  | 12 | 9 | 8 | 4 |  | 36.4 | 27.3 | 24.2 | 12.1 |  | -0.2142 | -0.3040 | 0.1555 |  | 8.062E-01 | 6.671E-01 | 7.317E-01 |
| 509 | Q96KP4 | CNDP2 | Cytosolic non-specific dipeptidase | 475 |  | 56 | 37 | 56 | 5 |  | 36.4 | 24.0 | 36.4 | 3.2 |  | -0.4264 | 0.2152 | -1.7067 |  | 1.410E-01 | 6.311E-01 | 1.687E-05 |
| 510 | P01920_1 | HLA-DQB1 | HLA class II histocompatibility antigen, DQ beta 1 chain | 261 |  | 8 | 0 | 14 | 0 |  | 36.4 | 0.0 | 63.6 | 0.0 |  | -2.7317 | 0.9365 | -1.3969 |  | 9.224E-03 | 1.853E-01 | 7.367E-02 |
| 511 | P62318 | SNRPD3 | Small nuclear ribonucleoprotein Sm D3 | 126 |  | 12 | 9 | 12 | 0 |  | 36.4 | 27.3 | 36.4 | 0.0 |  | -0.2142 | 0.2148 | -1.9156 |  | 8.062E-01 | 9.509E-01 | 1.533E-02 |
| 512 | P02792 | FTL | Ferritin light chain | 175 |  | 33 | 16 | 16 | 26 |  | 36.3 | 17.6 | 17.6 | 28.6 |  | -0.8341 | -0.7757 | 1.1637 |  | 4.358E-02 | 5.335E-02 | 7.343E-02 |
| 513 | O96009 | NAPSA | Napsin-A | 420 |  | 112 | 125 | 72 | 0 |  | 36.2 | 40.5 | 23.3 | 0.0 |  | 0.3147 | -0.4157 | -5.0171 |  | 1.536E-01 | 3.095E-02 | 2.098E-19 |
| 514 | P07384 | CAPN1 | Calpain-1 catalytic subunit | 714 |  | 21 | 11 | 26 | 0 |  | 36.2 | 19.0 | 44.8 | 0.0 |  | -0.7053 | 0.5077 | -2.6639 |  | 1.715E-01 | 3.390E-01 | 4.588E-04 |
| 515 | P43243 | MATR3 | Matrin-3 | 847 |  | 17 | 22 | 8 | 0 |  | 36.2 | 46.8 | 17.0 | 0.0 |  | 0.5061 | -0.7662 | -2.3778 |  | 3.668E-01 | 1.783E-01 | 2.177E-03 |
| 516 | P26641 | EEF1G | Elongation factor 1-gamma | 437 |  | 39 | 28 | 32 | 9 |  | 36.1 | 25.9 | 29.6 | 8.3 |  | -0.3047 | -0.0611 | -0.4831 |  | 3.772E-01 | 7.980E-01 | 5.667E-02 |
| 517 | P00558 | PGK1 | Phosphoglycerate kinase 1 | 417 |  | 118 | 76 | 81 | 52 |  | 36.1 | 23.2 | 24.8 | 15.9 |  | -0.4721 | -0.3227 | 0.3297 |  | 1.733E-02 | 7.185E-02 | 7.367E-01 |
| 518 | P09467 | FBP1 | Fructose-1,6-bisphosphatase 1 | 338 |  | 31 | 33 | 22 | 0 |  | 36.0 | 38.4 | 25.6 | 0.0 |  | 0.2434 | -0.2577 | -3.2000 |  | 6.456E-01 | 4.793E-01 | 9.421E-06 |
| 519 | P62263 | RPS14 | 40S ribosomal protein S14 | 151 |  | 18 | 15 | 17 | 0 |  | 36.0 | 30.0 | 34.0 | 0.0 |  | -0.0882 | 0.1378 | -2.4548 |  | 9.138E-01 | 9.862E-01 | 1.475E-03 |
| 520 | P16401 | HIST1H1B | Histone H1.5 | 226 |  | 37 | 29 | 37 | 0 |  | 35.9 | 28.2 | 35.9 | 0.0 |  | -0.1825 | 0.2150 | -3.4466 |  | 6.006E-01 | 7.258E-01 | 9.174E-07 |
| 521 | P04440_1 | HLA-DPB1 | HLA class II histocompatibility antigen, DP beta 1 chain | 258 |  | 5 | 0 | 9 | 0 |  | 35.7 | 0.0 | 64.3 | 0.0 |  | -2.1659 | 0.9287 | -0.8311 |  | 6.700E-02 | 3.137E-01 | 2.422E-01 |
| 522 | P05388 | RPLP0 | 60S acidic ribosomal protein P0 | 317 |  | 20 | 15 | 21 | 0 |  | 35.7 | 26.8 | 37.5 | 0.0 |  | -0.2309 | 0.2813 | -2.5975 |  | 6.672E-01 | 7.282E-01 | 6.770E-04 |
| 523 | Q9Y315 | DERA | Putative deoxyribose-phosphate aldolase | 318 |  | 5 | 6 | 3 | 0 |  | 35.7 | 42.9 | 21.4 | 0.0 |  | 0.3705 | -0.3418 | -0.8311 |  | 8.897E-01 | 8.408E-01 | 2.422E-01 |
| 524 | P46459 | NSF | Vesicle-fusing ATPase | 744 |  | 5 | 9 | 0 | 0 |  | 35.7 | 64.3 | 0.0 | 0.0 |  | 0.8703 | -2.1076 | -0.8311 |  | 3.365E-01 | 7.092E-02 | 2.422E-01 |
| 525 | P08238 | HSP90AB1 | Heat shock protein HSP 90-beta | 724 |  | 172 | 182 | 128 | 0 |  | 35.7 | 37.8 | 26.6 | 0.0 |  | 0.2391 | -0.2094 | -5.6341 |  | 2.041E-01 | 1.201E-01 | 1.558E-29 |
| 526 | Q8NBS9 | TXNDC5 | Thioredoxin domain-containing protein 5 | 432 |  | 21 | 16 | 22 | 0 |  | 35.6 | 27.1 | 37.3 | 0.0 |  | -0.2111 | 0.2784 | -2.6639 |  | 6.860E-01 | 7.219E-01 | 4.588E-04 |
| 527 | P08107_1 | HSPA1A | Heat shock 70 kDa protein 1A/1B | 641 |  | 146 | 119 | 104 | 42 |  | 35.5 | 29.0 | 25.3 | 10.2 |  | -0.1368 | -0.2713 | -0.2781 |  | 3.571E-01 | 8.044E-02 | 3.998E-03 |
| 528 | P35268 | RPL22 | 60S ribosomal protein L22 | 128 |  | 11 | 9 | 11 | 0 |  | 35.5 | 29.0 | 35.5 | 0.0 |  | -0.1009 | 0.2148 | -1.8023 |  | 9.707E-01 | 9.672E-01 | 2.267E-02 |
| 529 | Q10567 | AP1B1 | AP-1 complex subunit beta-1 | 949 |  | 11 | 11 | 9 | 0 |  | 35.5 | 35.5 | 29.0 | 0.0 |  | 0.1564 | -0.0425 | -1.8023 |  | 9.863E-01 | 9.849E-01 | 2.267E-02 |
| 530 | P07900 | HSP90AA1 | Heat shock protein HSP 90-alpha | 732 |  | 156 | 138 | 106 | 40 |  | 35.5 | 31.4 | 24.1 | 9.1 |  | -0.0192 | -0.3394 | -0.4421 |  | 7.827E-01 | 3.022E-02 | 3.559E-04 |
| 531 | P62136 | PPP1CA | Serine/threonine-protein phosphatase PP1-alpha catalytic subunit | 330 |  | 23 | 27 | 15 | 0 |  | 35.4 | 41.5 | 23.1 | 0.0 |  | 0.3770 | -0.3632 | -2.7882 |  | 4.712E-01 | 4.203E-01 | 2.108E-04 |
| 532 | P48444 | ARCN1 | Coatomer subunit delta | 511 |  | 6 | 2 | 9 | 0 |  | 35.3 | 11.8 | 52.9 | 0.0 |  | -1.0015 | 0.7145 | -1.0453 |  | 3.385E-01 | 4.671E-01 | 1.626E-01 |
| 533 | P00505 | GOT2 | Aspartate aminotransferase, mitochondrial | 430 |  | 25 | 23 | 21 | 2 |  | 35.2 | 32.4 | 29.6 | 2.8 |  | 0.0420 | -0.0239 | -1.5238 |  | 8.858E-01 | 9.404E-01 | 4.716E-03 |
| 534 | Q00839 | HNRNPU | Heterogeneous nuclear ribonucleoprotein U | 806 |  | 50 | 53 | 39 | 0 |  | 35.2 | 37.3 | 27.5 | 0.0 |  | 0.2389 | -0.1342 | -3.8694 |  | 5.338E-01 | 5.819E-01 | 5.915E-09 |
| 535 | P62820 | RAB1A | Ras-related protein Rab-1A | 205 |  | 26 | 22 | 26 | 0 |  | 35.1 | 29.7 | 35.1 | 0.0 |  | -0.0728 | 0.2149 | -2.9567 |  | 8.858E-01 | 8.005E-01 | 6.569E-05 |
| 536 | P07099 | EPHX1 | Epoxide hydrolase 1 | 455 |  | 39 | 43 | 29 | 0 |  | 35.1 | 38.7 | 26.1 | 0.0 |  | 0.2935 | -0.1977 | -3.5202 |  | 4.773E-01 | 5.176E-01 | 4.222E-07 |
| 537 | P00338 | LDHA | L-lactate dehydrogenase A chain | 332 |  | 61 | 44 | 45 | 24 |  | 35.1 | 25.3 | 25.9 | 13.8 |  | -0.3046 | -0.2145 | 0.1897 |  | 2.550E-01 | 3.665E-01 | 5.157E-01 |
| 538 | P04843 | RPN1 | Dolichyl-diphosphooligosaccharide--protein glycosyltransferase subunit 1 | 646 |  | 75 | 72 | 67 | 0 |  | 35.0 | 33.6 | 31.3 | 0.0 |  | 0.0987 | 0.0549 | -4.4441 |  | 8.593E-01 | 9.531E-01 | 3.627E-13 |
| 539 | P39019 | RPS19 | 40S ribosomal protein S19 | 145 |  | 28 | 22 | 30 | 0 |  | 35.0 | 27.5 | 37.5 | 0.0 |  | -0.1751 | 0.3105 | -3.0590 |  | 6.799E-01 | 5.858E-01 | 3.020E-05 |
| 540 | P84103 | SFRS3 | Serine/arginine-rich splicing factor 3 | 164 |  | 14 | 14 | 12 | 0 |  | 35.0 | 35.0 | 30.0 | 0.0 |  | 0.1564 | 0.0118 | -2.1185 |  | 9.743E-01 | 9.359E-01 | 7.015E-03 |
| 541 | O75531 | BANF1 | Barrier-to-autointegration factor | 89 |  | 14 | 15 | 11 | 0 |  | 35.0 | 37.5 | 27.5 | 0.0 |  | 0.2481 | -0.1014 | -2.1185 |  | 8.218E-01 | 8.972E-01 | 7.015E-03 |
| 542 | P48047 | ATP5O | ATP synthase subunit O, mitochondrial | 213 |  | 15 | 10 | 18 | 0 |  | 34.9 | 23.3 | 41.9 | 0.0 |  | -0.3745 | 0.4594 | -2.2102 |  | 5.530E-01 | 5.092E-01 | 4.748E-03 |
| 543 | Q16891 | IMMT | Mitochondrial inner membrane protein | 758 |  | 8 | 6 | 9 | 0 |  | 34.8 | 26.1 | 39.1 | 0.0 |  | -0.1953 | 0.3629 | -1.3969 |  | 9.116E-01 | 8.230E-01 | 7.367E-02 |
| 544 | P25789 | PSMA4 | Proteasome subunit alpha type-4 | 261 |  | 8 | 7 | 8 | 0 |  | 34.8 | 30.4 | 34.8 | 0.0 |  | -0.0088 | 0.2147 | -1.3969 |  | 8.713E-01 | 9.735E-01 | 7.367E-02 |
| 545 | P04406 | GAPDH | Glyceraldehyde-3-phosphate dehydrogenase | 335 |  | 170 | 118 | 128 | 74 |  | 34.7 | 24.1 | 26.1 | 15.1 |  | -0.3682 | -0.1926 | 0.3071 |  | 2.075E-02 | 1.474E-01 | 6.020E-01 |
| 546 | P62899 | RPL31 | 60S ribosomal protein L31 | 128 |  | 17 | 15 | 17 | 0 |  | 34.7 | 30.6 | 34.7 | 0.0 |  | -0.0112 | 0.2148 | -2.3778 |  | 9.524E-01 | 8.848E-01 | 2.177E-03 |
| 547 | P26368 | U2AF2 | Splicing factor U2AF 65 kDa subunit | 471 |  | 9 | 8 | 9 | 0 |  | 34.6 | 30.8 | 34.6 | 0.0 |  | 0.0082 | 0.2148 | -1.5450 |  | 8.631E-01 | 9.955E-01 | 4.970E-02 |
| 548 | P51571 | SSR4 | Translocon-associated protein subunit delta | 184 |  | 9 | 9 | 8 | 0 |  | 34.6 | 34.6 | 30.8 | 0.0 |  | 0.1564 | 0.0666 | -1.5450 |  | 9.534E-01 | 8.230E-01 | 4.970E-02 |
| 549 | G3V325 | ATP5J2-PTCD1 | ATP5J2-PTCD1 fusion protein;NA;cat | 749 |  | 9 | 9 | 8 | 0 |  | 34.6 | 34.6 | 30.8 | 0.0 |  | 0.1564 | 0.0666 | -1.5450 |  | 9.534E-01 | 8.230E-01 | 4.970E-02 |
| 550 | O94979 | SEC31A | Protein transport protein Sec31A | 1220 |  | 9 | 11 | 6 | 0 |  | 34.6 | 42.3 | 23.1 | 0.0 |  | 0.4136 | -0.2850 | -1.5450 |  | 6.813E-01 | 7.584E-01 | 4.970E-02 |
| 551 | P62277 | RPS13 | 40S ribosomal protein S13 | 151 |  | 11 | 11 | 10 | 0 |  | 34.4 | 34.4 | 31.3 | 0.0 |  | 0.1564 | 0.0918 | -1.8023 |  | 9.863E-01 | 8.505E-01 | 2.267E-02 |
| 552 | P47755 | CAPZA2 | F-actin-capping protein subunit alpha-2 | 286 |  | 13 | 7 | 13 | 5 |  | 34.2 | 18.4 | 34.2 | 13.2 |  | -0.6325 | 0.2148 | 0.3022 |  | 3.481E-01 | 9.359E-01 | 8.894E-01 |
| 553 | P21796 | VDAC1 | Voltage-dependent anion-selective channel protein 1 | 283 |  | 14 | 14 | 13 | 0 |  | 34.1 | 34.1 | 31.7 | 0.0 |  | 0.1564 | 0.1169 | -2.1185 |  | 9.743E-01 | 9.176E-01 | 7.015E-03 |
| 554 | P14678 | SNRPB | Small nuclear ribonucleoprotein-associated proteins B and B_ | 240 |  | 15 | 11 | 12 | 6 |  | 34.1 | 25.0 | 27.3 | 13.6 |  | -0.2515 | -0.0798 | 0.3269 |  | 7.068E-01 | 9.176E-01 | 9.116E-01 |
| 555 | P07437_1 | TUBB | Tubulin beta chain | 444 |  | 271 | 212 | 211 | 101 |  | 34.1 | 26.7 | 26.5 | 12.7 |  | -0.1982 | -0.1461 | 0.0791 |  | 8.305E-02 | 1.312E-01 | 4.342E-02 |
| 556 | P23284 | PPIB | Peptidyl-prolyl cis-trans isomerase B | 216 |  | 51 | 40 | 55 | 4 |  | 34.0 | 26.7 | 36.7 | 2.7 |  | -0.1851 | 0.3219 | -1.8263 |  | 5.147E-01 | 3.948E-01 | 2.017E-05 |
| 557 | Q9Y678 | COPG | Coatomer subunit gamma-1 | 874 |  | 20 | 20 | 19 | 0 |  | 33.9 | 33.9 | 32.2 | 0.0 |  | 0.1564 | 0.1453 | -2.5975 |  | 9.153E-01 | 9.854E-01 | 6.770E-04 |
| 558 | P05787 | KRT8 | Keratin, type II cytoskeletal 8 | 483 |  | 463 | 397 | 399 | 108 |  | 33.9 | 29.0 | 29.2 | 7.9 |  | -0.0662 | 0.0007 | -0.6059 |  | 3.169E-01 | 5.754E-01 | 1.588E-12 |
| 559 | P05783 | KRT18 | Keratin, type I cytoskeletal 18 | 430 |  | 288 | 271 | 259 | 36 |  | 33.7 | 31.7 | 30.3 | 4.2 |  | 0.0698 | 0.0630 | -1.4772 |  | 8.193E-01 | 9.882E-01 | 1.099E-18 |
| 560 | P46940 | IQGAP1 | Ras GTPase-activating-like protein IQGAP1 | 1657 |  | 95 | 75 | 97 | 15 |  | 33.7 | 26.6 | 34.4 | 5.3 |  | -0.1805 | 0.2454 | -1.0784 |  | 3.597E-01 | 4.090E-01 | 1.342E-05 |
| 561 | O43390 | HNRNPR | Heterogeneous nuclear ribonucleoprotein R | 636 |  | 36 | 37 | 31 | 3 |  | 33.6 | 34.6 | 29.0 | 2.8 |  | 0.1948 | 0.0067 | -1.6422 |  | 7.207E-01 | 9.641E-01 | 5.668E-04 |
| 562 | P26038 | MSN | Moesin | 577 |  | 146 | 107 | 118 | 63 |  | 33.6 | 24.7 | 27.2 | 14.5 |  | -0.2892 | -0.0902 | 0.2965 |  | 8.283E-02 | 4.325E-01 | 5.965E-01 |
| 563 | P11142 | HSPA8 | Heat shock cognate 71 kDa protein | 646 |  | 110 | 88 | 82 | 47 |  | 33.6 | 26.9 | 25.1 | 14.4 |  | -0.1623 | -0.2045 | 0.2873 |  | 3.639E-01 | 2.285E-01 | 6.218E-01 |
| 564 | P04792 | HSPB1 | Heat shock protein beta-1 | 205 |  | 120 | 97 | 112 | 28 |  | 33.6 | 27.2 | 31.4 | 7.8 |  | -0.1479 | 0.1168 | -0.5628 |  | 3.781E-01 | 8.136E-01 | 4.565E-04 |
| 565 | O14773 | TPP1 | Tripeptidyl-peptidase 1 | 563 |  | 8 | 8 | 6 | 2 |  | 33.3 | 33.3 | 25.0 | 8.3 |  | 0.1563 | -0.1369 | -0.0180 |  | 9.339E-01 | 9.486E-01 | 6.238E-01 |
| 566 | P37837 | TALDO1 | Transaldolase | 337 |  | 63 | 52 | 61 | 13 |  | 33.3 | 27.5 | 32.3 | 6.9 |  | -0.1149 | 0.1695 | -0.6832 |  | 6.272E-01 | 7.376E-01 | 4.774E-03 |
| 567 | P98095 | FBLN2 | Fibulin-2 | 1231 |  | 1 | 0 | 2 | 0 |  | 33.3 | 0.0 | 66.7 | 0.0 |  | -0.6918 | 0.7453 | 0.6431 |  | 9.666E-01 | 9.251E-01 | 7.108E-01 |
| 568 | P61803 | DAD1 | Dolichyl-diphosphooligosaccharide--protein glycosyltransferase subunit DAD1 | 113 |  | 1 | 0 | 2 | 0 |  | 33.3 | 0.0 | 66.7 | 0.0 |  | -0.6918 | 0.7453 | 0.6431 |  | 9.666E-01 | 9.251E-01 | 7.108E-01 |
| 569 | Q9C0C2 | TNKS1BP1 | 182 kDa tankyrase-1-binding protein | 1729 |  | 3 | 2 | 4 | 0 |  | 33.3 | 22.2 | 44.4 | 0.0 |  | -0.2308 | 0.5196 | -0.2746 |  | 9.255E-01 | 8.858E-01 | 5.442E-01 |
| 570 | Q5JXB2 | UBE2NL | putative ubiquitin-conjugating enzyme E2 N-like;HIX0056502 HIT000649038 HIP000025901;cat | 153 |  | 4 | 3 | 5 | 0 |  | 33.3 | 25.0 | 41.7 | 0.0 |  | -0.1486 | 0.4663 | -0.5795 |  | 9.119E-01 | 8.707E-01 | 3.621E-01 |
| 571 | P46781 | RPS9 | 40S ribosomal protein S9 | 194 |  | 35 | 31 | 39 | 0 |  | 33.3 | 29.5 | 37.1 | 0.0 |  | -0.0124 | 0.3663 | -3.3690 |  | 9.763E-01 | 4.144E-01 | 1.994E-06 |
| 572 | P05386 | RPLP1 | 60S acidic ribosomal protein P1 | 114 |  | 9 | 8 | 10 | 0 |  | 33.3 | 29.6 | 37.0 | 0.0 |  | 0.0082 | 0.3491 | -1.5450 |  | 8.631E-01 | 8.130E-01 | 4.970E-02 |
| 573 | Q9NR28 | DIABLO | Diablo homolog, mitochondrial | 239 |  | 6 | 6 | 6 | 0 |  | 33.3 | 33.3 | 33.3 | 0.0 |  | 0.1563 | 0.2147 | -1.0453 |  | 8.854E-01 | 9.195E-01 | 1.626E-01 |
| 574 | Q13404_1 | UBE2V1 | Ubiquitin-conjugating enzyme E2 variant 1 | 170 |  | 8 | 8 | 8 | 0 |  | 33.3 | 33.3 | 33.3 | 0.0 |  | 0.1563 | 0.2147 | -1.3969 |  | 9.339E-01 | 9.735E-01 | 7.367E-02 |
| 575 | Q8N0U8 | VKORC1L1 | Vitamin K epoxide reductase complex subunit 1-like protein 1 | 176 |  | 9 | 9 | 9 | 0 |  | 33.3 | 33.3 | 33.3 | 0.0 |  | 0.1564 | 0.2148 | -1.5450 |  | 9.534E-01 | 9.955E-01 | 4.970E-02 |
| 576 | Q01081 | U2AF1 | Splicing factor U2AF 35 kDa subunit | 240 |  | 9 | 9 | 9 | 0 |  | 33.3 | 33.3 | 33.3 | 0.0 |  | 0.1564 | 0.2148 | -1.5450 |  | 9.534E-01 | 9.955E-01 | 4.970E-02 |
| 577 | P47756 | CAPZB | F-actin-capping protein subunit beta | 277 |  | 9 | 9 | 9 | 0 |  | 33.3 | 33.3 | 33.3 | 0.0 |  | 0.1564 | 0.2148 | -1.5450 |  | 9.534E-01 | 9.955E-01 | 4.970E-02 |
| 578 | P35270 | SPR | Sepiapterin reductase | 261 |  | 12 | 12 | 12 | 0 |  | 33.3 | 33.3 | 33.3 | 0.0 |  | 0.1564 | 0.2148 | -1.9156 |  | 9.995E-01 | 9.509E-01 | 1.533E-02 |
| 579 | Q86U42 | PABPN1 | Polyadenylate-binding protein 2 | 306 |  | 10 | 13 | 7 | 0 |  | 33.3 | 43.3 | 23.3 | 0.0 |  | 0.4976 | -0.2329 | -1.6794 |  | 5.366E-01 | 7.930E-01 | 3.355E-02 |
| 580 | P62304 | SNRPE | Small nuclear ribonucleoprotein E | 92 |  | 10 | 13 | 7 | 0 |  | 33.3 | 43.3 | 23.3 | 0.0 |  | 0.4976 | -0.2329 | -1.6794 |  | 5.366E-01 | 7.930E-01 | 3.355E-02 |
| 581 | Q9Y6K5 | OAS3 | 2_-5_-oligoadenylate synthase 3 | 1087 |  | 3 | 6 | 0 | 0 |  | 33.3 | 66.7 | 0.0 | 0.0 |  | 0.9270 | -1.5510 | -0.2746 |  | 4.269E-01 | 2.641E-01 | 5.442E-01 |
| 582 | Q9ULZ3 | PYCARD | Apoptosis-associated speck-like protein containing a CARD | 195 |  | 2 | 4 | 0 | 0 |  | 33.3 | 66.7 | 0.0 | 0.0 |  | 0.8483 | -1.1640 | 0.1125 |  | 6.090E-01 | 5.166E-01 | 8.264E-01 |
| 583 | Q92896 | GLG1 | Golgi apparatus protein 1 | 1203 |  | 1 | 2 | 0 | 0 |  | 33.3 | 66.7 | 0.0 | 0.0 |  | 0.6869 | -0.6334 | 0.6431 |  | 9.423E-01 | 9.567E-01 | 7.108E-01 |
| 584 | Q8WUM4 | PDCD6IP | Programmed cell death 6-interacting protein | 868 |  | 1 | 2 | 0 | 0 |  | 33.3 | 66.7 | 0.0 | 0.0 |  | 0.6869 | -0.6334 | 0.6431 |  | 9.423E-01 | 9.567E-01 | 7.108E-01 |
| 585 | O43169 | CYB5B | Cytochrome b5 type B | 146 |  | 1 | 2 | 0 | 0 |  | 33.3 | 66.7 | 0.0 | 0.0 |  | 0.6869 | -0.6334 | 0.6431 |  | 9.423E-01 | 9.567E-01 | 7.108E-01 |
| 586 | P68371 | TUBB2C | Tubulin beta-4B chain | 445 |  | 251 | 217 | 208 | 81 |  | 33.2 | 28.7 | 27.5 | 10.7 |  | -0.0531 | -0.0555 | -0.1271 |  | 5.320E-01 | 4.211E-01 | 2.494E-03 |
| 587 | P07910 | HNRNPC | Heterogeneous nuclear ribonucleoproteins C1/C2 | 306 |  | 54 | 51 | 58 | 0 |  | 33.1 | 31.3 | 35.6 | 0.0 |  | 0.0759 | 0.3163 | -3.9781 |  | 9.696E-01 | 3.905E-01 | 1.253E-09 |
| 588 | P12956 | XRCC6 | X-ray repair cross-complementing protein 6 | 609 |  | 48 | 44 | 51 | 2 |  | 33.1 | 30.3 | 35.2 | 1.4 |  | 0.0341 | 0.3007 | -2.4330 |  | 9.296E-01 | 4.583E-01 | 1.990E-06 |
| 589 | P13639 | EEF2 | Elongation factor 2 | 858 |  | 40 | 44 | 37 | 0 |  | 33.1 | 36.4 | 30.6 | 0.0 |  | 0.2904 | 0.1059 | -3.5557 |  | 4.769E-01 | 9.841E-01 | 2.864E-07 |
| 590 | P26599 | PTBP1 | Polypyrimidine tract-binding protein 1 | 557 |  | 27 | 33 | 22 | 0 |  | 32.9 | 40.2 | 26.8 | 0.0 |  | 0.4347 | -0.0664 | -3.0087 |  | 3.319E-01 | 8.474E-01 | 4.454E-05 |
| 591 | P53999 | SUB1 | Activated RNA polymerase II transcriptional coactivator p15 | 127 |  | 19 | 21 | 18 | 0 |  | 32.8 | 36.2 | 31.0 | 0.0 |  | 0.2924 | 0.1417 | -2.5279 |  | 6.724E-01 | 9.992E-01 | 9.991E-04 |
| 592 | Q92841 | DDX17 | Probable ATP-dependent RNA helicase DDX17 | 729 |  | 18 | 17 | 20 | 0 |  | 32.7 | 30.9 | 36.4 | 0.0 |  | 0.0794 | 0.3576 | -2.4548 |  | 9.276E-01 | 6.193E-01 | 1.475E-03 |
| 593 | P08727 | KRT19 | Keratin, type I cytoskeletal 19 | 400 |  | 368 | 368 | 315 | 78 |  | 32.6 | 32.6 | 27.9 | 6.9 |  | 0.1589 | -0.0090 | -0.7381 |  | 2.687E-01 | 5.632E-01 | 3.650E-12 |
| 594 | P00441 | SOD1 | Superoxide dismutase [Cu-Zn] | 154 |  | 14 | 13 | 10 | 6 |  | 32.6 | 30.2 | 23.3 | 14.0 |  | 0.0585 | -0.2244 | 0.4186 |  | 8.666E-01 | 7.279E-01 | 9.679E-01 |
| 595 | P24539 | ATP5F1 | ATP synthase subunit b, mitochondrial | 256 |  | 13 | 13 | 14 | 0 |  | 32.5 | 32.5 | 35.0 | 0.0 |  | 0.1564 | 0.3127 | -2.0206 |  | 9.864E-01 | 7.779E-01 | 1.037E-02 |
| 596 | Q01469 | FABP5 | Fatty acid-binding protein, epidermal | 135 |  | 12 | 6 | 0 | 19 |  | 32.4 | 16.2 | 0.0 | 51.4 |  | -0.7140 | -3.1920 | 2.1056 |  | 3.123E-01 | 7.658E-04 | 1.617E-03 |
| 597 | P48735 | IDH2 | Isocitrate dehydrogenase [NADP], mitochondrial | 452 |  | 52 | 46 | 63 | 0 |  | 32.3 | 28.6 | 39.1 | 0.0 |  | -0.0162 | 0.4869 | -3.9248 |  | 9.271E-01 | 1.294E-01 | 2.722E-09 |
| 598 | P52597 | HNRNPF | Heterogeneous nuclear ribonucleoprotein F | 415 |  | 10 | 9 | 12 | 0 |  | 32.3 | 29.0 | 38.7 | 0.0 |  | 0.0220 | 0.4510 | -1.6794 |  | 8.553E-01 | 6.399E-01 | 3.355E-02 |
| 599 | O75390 | CS | Citrate synthase, mitochondrial | 466 |  | 19 | 23 | 17 | 0 |  | 32.2 | 39.0 | 28.8 | 0.0 |  | 0.4168 | 0.0647 | -2.5279 |  | 4.627E-01 | 8.738E-01 | 9.991E-04 |
| 600 | P68104 | EEF1A1 | Elongation factor 1-alpha 1 | 462 |  | 109 | 95 | 88 | 47 |  | 32.2 | 28.0 | 26.0 | 13.9 |  | -0.0398 | -0.0906 | 0.3004 |  | 7.534E-01 | 5.048E-01 | 6.605E-01 |
| 601 | P40925 | MDH1 | Malate dehydrogenase, cytoplasmic | 334 |  | 9 | 9 | 10 | 0 |  | 32.1 | 32.1 | 35.7 | 0.0 |  | 0.1564 | 0.3491 | -1.5450 |  | 9.534E-01 | 8.130E-01 | 4.970E-02 |
| 602 | P37108 | SRP14 | Signal recognition particle 14 kDa protein | 136 |  | 9 | 10 | 9 | 0 |  | 32.1 | 35.7 | 32.1 | 0.0 |  | 0.2907 | 0.2148 | -1.5450 |  | 8.553E-01 | 9.955E-01 | 4.970E-02 |
| 603 | P13804 | ETFA | Electron transfer flavoprotein subunit alpha, mitochondrial | 333 |  | 9 | 15 | 4 | 0 |  | 32.1 | 53.6 | 14.3 | 0.0 |  | 0.8216 | -0.7508 | -1.5450 |  | 2.191E-01 | 3.551E-01 | 4.970E-02 |
| 604 | P06576 | ATP5B | ATP synthase subunit beta, mitochondrial | 529 |  | 161 | 160 | 146 | 34 |  | 32.1 | 31.9 | 29.1 | 6.8 |  | 0.1484 | 0.0752 | -0.7154 |  | 5.223E-01 | 9.705E-01 | 5.349E-06 |
| 605 | P62937 | PPIA | Peptidyl-prolyl cis-trans isomerase A | 165 |  | 94 | 84 | 80 | 35 |  | 32.1 | 28.7 | 27.3 | 11.9 |  | -0.0038 | -0.0148 | 0.0977 |  | 9.067E-01 | 7.861E-01 | 2.599E-01 |
| 606 | P30043 | BLVRB | Flavin reductase (NADPH) | 206 |  | 25 | 18 | 12 | 23 |  | 32.1 | 23.1 | 15.4 | 29.5 |  | -0.2915 | -0.7723 | 1.3792 |  | 5.201E-01 | 9.594E-02 | 3.305E-02 |
| 607 | Q14847 | LASP1 | LIM and SH3 domain protein 1 | 261 |  | 24 | 24 | 27 | 0 |  | 32.0 | 32.0 | 36.0 | 0.0 |  | 0.1565 | 0.3771 | -2.8466 |  | 8.844E-01 | 5.042E-01 | 1.429E-04 |
| 608 | Q14108 | SCARB2 | Lysosome membrane protein 2 | 478 |  | 8 | 8 | 9 | 0 |  | 32.0 | 32.0 | 36.0 | 0.0 |  | 0.1563 | 0.3629 | -1.3969 |  | 9.339E-01 | 8.230E-01 | 7.367E-02 |
| 609 | P62258 | YWHAE | 14-3-3 protein epsilon | 255 |  | 105 | 78 | 84 | 62 |  | 31.9 | 23.7 | 25.5 | 18.8 |  | -0.2678 | -0.1035 | 0.7470 |  | 1.728E-01 | 4.760E-01 | 1.930E-01 |
| 610 | P62269 | RPS18 | 40S ribosomal protein S18 | 152 |  | 36 | 36 | 33 | 8 |  | 31.9 | 31.9 | 29.2 | 7.1 |  | 0.1565 | 0.0937 | -0.5194 |  | 8.125E-01 | 9.751E-01 | 5.756E-02 |
| 611 | P63104 | YWHAZ | 14-3-3 protein zeta/delta | 245 |  | 138 | 124 | 108 | 66 |  | 31.7 | 28.4 | 24.8 | 15.1 |  | 0.0034 | -0.1361 | 0.4440 |  | 8.990E-01 | 3.153E-01 | 9.930E-01 |
| 612 | P29401 | TKT | Transketolase | 623 |  | 162 | 149 | 171 | 30 |  | 31.6 | 29.1 | 33.4 | 5.9 |  | 0.0368 | 0.2943 | -0.8987 |  | 9.546E-01 | 1.517E-01 | 2.896E-07 |
| 613 | Q99623 | PHB2 | Prohibitin-2 | 299 |  | 56 | 55 | 56 | 10 |  | 31.6 | 31.1 | 31.6 | 5.6 |  | 0.1312 | 0.2152 | -0.8579 |  | 8.018E-01 | 6.311E-01 | 2.713E-03 |
| 614 | P13797 | PLS3 | Plastin-3 | 630 |  | 31 | 48 | 19 | 0 |  | 31.6 | 49.0 | 19.4 | 0.0 |  | 0.7685 | -0.4572 | -3.2000 |  | 2.934E-02 | 2.376E-01 | 9.421E-06 |
| 615 | Q96IU4 | ABHD14B | Abhydrolase domain-containing protein 14B | 210 |  | 12 | 10 | 16 | 0 |  | 31.6 | 26.3 | 42.1 | 0.0 |  | -0.0798 | 0.5957 | -1.9156 |  | 9.863E-01 | 3.927E-01 | 1.533E-02 |
| 616 | P52565 | ARHGDIA | Rho GDP-dissociation inhibitor 1 | 204 |  | 16 | 19 | 16 | 0 |  | 31.4 | 37.3 | 31.4 | 0.0 |  | 0.3879 | 0.2148 | -2.2964 |  | 5.580E-01 | 8.965E-01 | 3.215E-03 |
| 617 | Q16181 | SEPT7 | Septin-7 | 437 |  | 5 | 2 | 9 | 0 |  | 31.3 | 12.5 | 56.3 | 0.0 |  | -0.7873 | 0.9287 | -0.8311 |  | 5.147E-01 | 3.137E-01 | 2.422E-01 |
| 618 | P62851 | RPS25 | 40S ribosomal protein S25 | 125 |  | 5 | 3 | 8 | 0 |  | 31.3 | 18.8 | 50.0 | 0.0 |  | -0.4002 | 0.7805 | -0.8311 |  | 8.135E-01 | 4.525E-01 | 2.422E-01 |
| 619 | P25787 | PSMA2 | Proteasome subunit alpha type-2 | 234 |  | 5 | 7 | 4 | 0 |  | 31.3 | 43.8 | 25.0 | 0.0 |  | 0.5570 | -0.0369 | -0.8311 |  | 6.645E-01 | 8.707E-01 | 2.422E-01 |
| 620 | Q99584 | S100A13 | Protein S100-A13 | 98 |  | 10 | 15 | 7 | 0 |  | 31.3 | 46.9 | 21.9 | 0.0 |  | 0.6872 | -0.2329 | -1.6794 |  | 3.121E-01 | 7.930E-01 | 3.355E-02 |
| 621 | P20592 | MX2 | Interferon-induced GTP-binding protein Mx2 | 715 |  | 5 | 11 | 0 | 0 |  | 31.3 | 68.8 | 0.0 | 0.0 |  | 1.1276 | -2.1076 | -0.8311 |  | 1.536E-01 | 7.092E-02 | 2.422E-01 |
| 622 | P23528 | CFL1 | Cofilin-1 | 166 |  | 38 | 27 | 39 | 18 |  | 31.1 | 22.1 | 32.0 | 14.8 |  | -0.3186 | 0.2514 | 0.4641 |  | 3.644E-01 | 6.337E-01 | 8.939E-01 |
| 623 | P84243 | H3F3A | Histone H3.3 | 136 |  | 33 | 34 | 39 | 0 |  | 31.1 | 32.1 | 36.8 | 0.0 |  | 0.1981 | 0.4483 | -3.2870 |  | 7.319E-01 | 2.934E-01 | 4.334E-06 |
| 624 | Q00610 | CLTC | Clathrin heavy chain 1 | 1675 |  | 133 | 160 | 116 | 21 |  | 30.9 | 37.2 | 27.0 | 4.9 |  | 0.4234 | 0.0194 | -1.1064 |  | 2.494E-02 | 8.736E-01 | 2.051E-07 |
| 625 | Q14195 | DPYSL3 | Dihydropyrimidinase-related protein 3 | 570 |  | 21 | 19 | 28 | 0 |  | 30.9 | 27.9 | 41.2 | 0.0 |  | 0.0204 | 0.6100 | -2.6639 |  | 9.153E-01 | 2.156E-01 | 4.588E-04 |
| 626 | P15311 | EZR | Ezrin | 586 |  | 88 | 81 | 79 | 38 |  | 30.8 | 28.3 | 27.6 | 13.3 |  | 0.0386 | 0.0615 | 0.3071 |  | 9.425E-01 | 9.415E-01 | 7.124E-01 |
| 627 | P16070 | CD44 | CD44 antigen | 742 |  | 8 | 9 | 9 | 0 |  | 30.8 | 34.6 | 34.6 | 0.0 |  | 0.3045 | 0.3629 | -1.3969 |  | 8.631E-01 | 8.230E-01 | 7.367E-02 |
| 628 | P18085 | ARF4 | ADP-ribosylation factor 4 | 180 |  | 31 | 36 | 34 | 0 |  | 30.7 | 35.6 | 33.7 | 0.0 |  | 0.3648 | 0.3435 | -3.2000 |  | 4.056E-01 | 4.927E-01 | 9.421E-06 |
| 629 | P22626 | HNRNPA2B1 | Heterogeneous nuclear ribonucleoproteins A2/B1 | 353 |  | 128 | 110 | 124 | 56 |  | 30.6 | 26.3 | 29.7 | 13.4 |  | -0.0604 | 0.1703 | 0.3181 |  | 6.464E-01 | 5.839E-01 | 6.881E-01 |
| 630 | Q15365 | PCBP1 | Poly(rC)-binding protein 1 | 356 |  | 18 | 22 | 19 | 0 |  | 30.5 | 37.3 | 32.2 | 0.0 |  | 0.4291 | 0.2880 | -2.4548 |  | 4.597E-01 | 7.413E-01 | 1.475E-03 |
| 631 | P50225 | SULT1A1 | Sulfotransferase 1A1 | 295 |  | 25 | 25 | 19 | 13 |  | 30.5 | 30.5 | 23.2 | 15.9 |  | 0.1565 | -0.1599 | 0.6105 |  | 8.773E-01 | 6.925E-01 | 9.144E-01 |
| 632 | O00148 | DDX39 | ATP-dependent RNA helicase DDX39A | 427 |  | 27 | 26 | 28 | 8 |  | 30.3 | 29.2 | 31.5 | 9.0 |  | 0.1044 | 0.2652 | -0.1199 |  | 9.764E-01 | 6.872E-01 | 3.110E-01 |
| 633 | P31949 | S100A11 | Protein S100-A11 | 105 |  | 37 | 48 | 37 | 0 |  | 30.3 | 39.3 | 30.3 | 0.0 |  | 0.5219 | 0.2150 | -3.4466 |  | 1.409E-01 | 7.258E-01 | 9.174E-07 |
| 634 | P51572 | BCAP31 | B-cell receptor-associated protein 31 | 313 |  | 20 | 11 | 35 | 0 |  | 30.3 | 16.7 | 53.0 | 0.0 |  | -0.6389 | 0.9864 | -2.5975 |  | 2.247E-01 | 2.144E-02 | 6.770E-04 |
| 635 | O15144 | ARPC2 | Actin-related protein 2/3 complex subunit 2 | 300 |  | 10 | 10 | 13 | 0 |  | 30.3 | 30.3 | 39.4 | 0.0 |  | 0.1564 | 0.5560 | -1.6794 |  | 9.707E-01 | 4.980E-01 | 3.355E-02 |
| 636 | O14979 | HNRPDL | Heterogeneous nuclear ribonucleoprotein D-like | 420 |  | 10 | 12 | 11 | 0 |  | 30.3 | 36.4 | 33.3 | 0.0 |  | 0.3926 | 0.3377 | -1.6794 |  | 6.822E-01 | 8.036E-01 | 3.355E-02 |
| 637 | P30044 | PRDX5 | Peroxiredoxin-5, mitochondrial | 214 |  | 63 | 59 | 62 | 24 |  | 30.3 | 28.4 | 29.8 | 11.5 |  | 0.0637 | 0.1926 | 0.1440 |  | 9.928E-01 | 6.685E-01 | 4.278E-01 |
| 638 | P61978 | HNRNPK | Heterogeneous nuclear ribonucleoprotein K | 463 |  | 112 | 123 | 101 | 35 |  | 30.2 | 33.2 | 27.2 | 9.4 |  | 0.2915 | 0.0676 | -0.1531 |  | 1.947E-01 | 9.752E-01 | 3.428E-02 |
| 639 | P37802 | TAGLN2 | Transgelin-2 | 199 |  | 150 | 156 | 142 | 49 |  | 30.2 | 31.4 | 28.6 | 9.9 |  | 0.2139 | 0.1372 | -0.0993 |  | 3.074E-01 | 6.881E-01 | 2.553E-02 |
| 640 | P01009 | SERPINA1 | Alpha-1-antitrypsin | 418 |  | 28 | 17 | 19 | 29 |  | 30.1 | 18.3 | 20.4 | 31.2 |  | -0.5248 | -0.3162 | 1.5429 |  | 2.231E-01 | 4.241E-01 | 5.082E-03 |
| 641 | P14174 | MIF | Macrophage migration inhibitory factor | 115 |  | 21 | 17 | 18 | 14 |  | 30.0 | 24.3 | 25.7 | 20.0 |  | -0.1297 | 0.0057 | 0.9472 |  | 8.185E-01 | 9.854E-01 | 4.177E-01 |
| 642 | P08865 | RPSA | 40S ribosomal protein SA | 295 |  | 30 | 28 | 32 | 10 |  | 30.0 | 28.0 | 32.0 | 10.0 |  | 0.0609 | 0.3046 | 0.0171 |  | 9.397E-01 | 5.792E-01 | 4.211E-01 |
| 643 | O14880 | MGST3 | Microsomal glutathione S-transferase 3 | 152 |  | 6 | 9 | 5 | 0 |  | 30.0 | 45.0 | 25.0 | 0.0 |  | 0.6561 | 0.0005 | -1.0453 |  | 4.971E-01 | 8.572E-01 | 1.626E-01 |
| 644 | Q8NFU3 | TSTD1 | Thiosulfate sulfurtransferase/rhodanese-like domain-containing protein 1 | 115 |  | 3 | 5 | 2 | 0 |  | 30.0 | 50.0 | 20.0 | 0.0 |  | 0.7128 | -0.1724 | -0.2746 |  | 6.368E-01 | 9.034E-01 | 5.442E-01 |
| 645 | Q96NY7 | CLIC6 | Chloride intracellular channel protein 6 | 704 |  | 9 | 21 | 0 | 0 |  | 30.0 | 70.0 | 0.0 | 0.0 |  | 1.2753 | -2.8215 | -1.5450 |  | 2.386E-02 | 5.301E-03 | 4.970E-02 |
| 646 | Q00341 | HDLBP | Vigilin | 1268 |  | 3 | 7 | 0 | 0 |  | 30.0 | 70.0 | 0.0 | 0.0 |  | 1.1135 | -1.5510 | -0.2746 |  | 2.768E-01 | 2.641E-01 | 5.442E-01 |
| 647 | P53621 | COPA | Coatomer subunit alpha | 1233 |  | 20 | 24 | 23 | 0 |  | 29.9 | 35.8 | 34.3 | 0.0 |  | 0.4055 | 0.4056 | -2.5975 |  | 4.653E-01 | 5.085E-01 | 6.770E-04 |
| 648 | P60709 | ACTB | Actin, cytoplasmic 1 | 375 |  | 675 | 637 | 638 | 315 |  | 29.8 | 28.1 | 28.2 | 13.9 |  | 0.0750 | 0.1376 | 0.4078 |  | 6.572E-01 | 3.526E-01 | 8.856E-01 |
| 649 | P09651 | HNRNPA1 | Heterogeneous nuclear ribonucleoprotein A1 | 320 |  | 59 | 57 | 67 | 15 |  | 29.8 | 28.8 | 33.8 | 7.6 |  | 0.1079 | 0.3957 | -0.4004 |  | 8.634E-01 | 2.170E-01 | 3.285E-02 |
| 650 | P06748 | NPM1 | Nucleophosmin | 294 |  | 63 | 75 | 66 | 8 |  | 29.7 | 35.4 | 31.1 | 3.8 |  | 0.4046 | 0.2813 | -1.3075 |  | 1.527E-01 | 4.275E-01 | 7.488E-05 |
| 651 | P60953 | CDC42 | Cell division control protein 42 homolog | 191 |  | 24 | 24 | 26 | 7 |  | 29.6 | 29.6 | 32.1 | 8.6 |  | 0.1565 | 0.3250 | -0.1229 |  | 8.844E-01 | 5.993E-01 | 3.345E-01 |
| 652 | Q96TA1 | FAM129B | Niban-like protein 1 | 733 |  | 8 | 10 | 8 | 1 |  | 29.6 | 37.0 | 29.6 | 3.7 |  | 0.4389 | 0.2147 | -0.5487 |  | 6.794E-01 | 9.735E-01 | 2.935E-01 |
| 653 | O75367 | H2AFY | Core histone macro-H2A.1 | 371 |  | 40 | 50 | 45 | 0 |  | 29.6 | 37.0 | 33.3 | 0.0 |  | 0.4704 | 0.3805 | -3.5557 |  | 1.779E-01 | 3.498E-01 | 2.864E-07 |
| 654 | O14950 | MYL12B | Myosin regulatory light chain 12B | 172 |  | 53 | 54 | 50 | 23 |  | 29.4 | 30.0 | 27.8 | 12.8 |  | 0.1831 | 0.1329 | 0.3302 |  | 6.650E-01 | 8.757E-01 | 8.280E-01 |
| 655 | P28482 | MAPK1 | Mitogen-activated protein kinase 1 | 360 |  | 5 | 8 | 4 | 0 |  | 29.4 | 47.1 | 23.5 | 0.0 |  | 0.7221 | -0.0369 | -0.8311 |  | 4.799E-01 | 8.707E-01 | 2.422E-01 |
| 656 | P20700 | LMNB1 | Lamin-B1 | 586 |  | 54 | 58 | 59 | 13 |  | 29.3 | 31.5 | 32.1 | 7.1 |  | 0.2578 | 0.3405 | -0.4649 |  | 4.673E-01 | 3.400E-01 | 2.902E-02 |
| 657 | P50395 | GDI2 | Rab GDP dissociation inhibitor beta | 445 |  | 39 | 50 | 37 | 7 |  | 29.3 | 37.6 | 27.8 | 5.3 |  | 0.5059 | 0.1414 | -0.7966 |  | 1.452E-01 | 8.975E-01 | 1.484E-02 |
| 658 | P04844 | RPN2 | Dolichyl-diphosphooligosaccharide--protein glycosyltransferase subunit 2 | 631 |  | 17 | 20 | 18 | 3 |  | 29.3 | 34.5 | 31.0 | 5.2 |  | 0.3762 | 0.2919 | -0.6117 |  | 5.596E-01 | 7.481E-01 | 1.402E-01 |
| 659 | P08729 | KRT7 | Keratin, type II cytoskeletal 7 | 469 |  | 262 | 293 | 287 | 56 |  | 29.2 | 32.6 | 32.0 | 6.2 |  | 0.3208 | 0.3499 | -0.7163 |  | 2.309E-02 | 2.094E-02 | 7.148E-09 |
| 660 | Q92522 | H1FX | Histone H1x | 213 |  | 7 | 7 | 8 | 2 |  | 29.2 | 29.2 | 33.3 | 8.3 |  | 0.1563 | 0.3799 | 0.1471 |  | 9.116E-01 | 8.335E-01 | 7.794E-01 |
| 661 | Q07021 | C1QBP | Complement component 1 Q subcomponent-binding protein, mitochondrial | 282 |  | 9 | 11 | 11 | 0 |  | 29.0 | 35.5 | 35.5 | 0.0 |  | 0.4136 | 0.4721 | -1.5450 |  | 6.813E-01 | 6.409E-01 | 4.970E-02 |
| 662 | P51991 | HNRNPA3 | Heterogeneous nuclear ribonucleoprotein A3 | 378 |  | 40 | 42 | 43 | 13 |  | 29.0 | 30.4 | 31.2 | 9.4 |  | 0.2250 | 0.3166 | -0.0425 |  | 6.244E-01 | 4.749E-01 | 2.924E-01 |
| 663 | P00403 | MT-CO2 | Cytochrome c oxidase subunit 2 | 227 |  | 31 | 33 | 31 | 12 |  | 29.0 | 30.8 | 29.0 | 11.2 |  | 0.2434 | 0.2150 | 0.2080 |  | 6.456E-01 | 7.639E-01 | 6.696E-01 |
| 664 | P20036 | HLA-DPA1 | HLA class II histocompatibility antigen, DP alpha 1 chain | 260 |  | 11 | 10 | 17 | 0 |  | 28.9 | 26.3 | 44.7 | 0.0 |  | 0.0334 | 0.7903 | -1.8023 |  | 8.480E-01 | 2.171E-01 | 2.267E-02 |
| 665 | P07305 | H1F0 | Histone H1.0 | 194 |  | 25 | 22 | 28 | 12 |  | 28.7 | 25.3 | 32.2 | 13.8 |  | -0.0188 | 0.3712 | 0.5053 |  | 9.959E-01 | 5.028E-01 | 8.962E-01 |
| 666 | P84090 | ERH | Enhancer of rudimentary homolog | 104 |  | 12 | 13 | 15 | 2 |  | 28.6 | 31.0 | 35.7 | 4.8 |  | 0.2614 | 0.5094 | -0.5367 |  | 8.343E-01 | 5.047E-01 | 2.295E-01 |
| 667 | P62913 | RPL11 | 60S ribosomal protein L11 | 178 |  | 4 | 2 | 8 | 0 |  | 28.6 | 14.3 | 57.1 | 0.0 |  | -0.5357 | 1.0321 | -0.5795 |  | 7.588E-01 | 2.901E-01 | 3.621E-01 |
| 668 | P61916 | NPC2 | Epididymal secretory protein E1 | 151 |  | 2 | 1 | 4 | 0 |  | 28.6 | 14.3 | 57.1 | 0.0 |  | -0.3743 | 0.9067 | 0.1125 |  | 9.423E-01 | 5.879E-01 | 8.264E-01 |
| 669 | Q9UBI6 | GNG12 | Guanine nucleotide-binding protein G(I)/G(S)/G(O) subunit gamma-12 | 72 |  | 2 | 2 | 3 | 0 |  | 28.6 | 28.6 | 42.9 | 0.0 |  | 0.1563 | 0.6018 | 0.1125 |  | 6.748E-01 | 9.034E-01 | 8.264E-01 |
| 670 | P01903 | HLA-DRA | HLA class II histocompatibility antigen, DR alpha chain | 254 |  | 40 | 41 | 59 | 0 |  | 28.6 | 29.3 | 42.1 | 0.0 |  | 0.1912 | 0.7630 | -3.5557 |  | 7.064E-01 | 1.851E-02 | 2.864E-07 |
| 671 | Q9BSJ8 | ESYT1 | Extended synaptotagmin-1 | 1104 |  | 12 | 14 | 16 | 0 |  | 28.6 | 33.3 | 38.1 | 0.0 |  | 0.3593 | 0.5957 | -1.9156 |  | 6.822E-01 | 3.927E-01 | 1.533E-02 |
| 672 | P36542 | ATP5C1 | ATP synthase subunit gamma, mitochondrial | 298 |  | 8 | 10 | 10 | 0 |  | 28.6 | 35.7 | 35.7 | 0.0 |  | 0.4389 | 0.4973 | -1.3969 |  | 6.794E-01 | 6.412E-01 | 7.367E-02 |
| 673 | P51648 | ALDH3A2 | Fatty aldehyde dehydrogenase | 508 |  | 6 | 9 | 6 | 0 |  | 28.6 | 42.9 | 28.6 | 0.0 |  | 0.6561 | 0.2147 | -1.0453 |  | 4.971E-01 | 9.195E-01 | 1.626E-01 |
| 674 | Q14157 | UBAP2L | Ubiquitin-associated protein 2-like | 1087 |  | 4 | 6 | 4 | 0 |  | 28.6 | 42.9 | 28.6 | 0.0 |  | 0.6221 | 0.2147 | -0.5795 |  | 6.536E-01 | 8.408E-01 | 3.621E-01 |
| 675 | Q9H4A4 | RNPEP | Aminopeptidase B | 650 |  | 8 | 20 | 0 | 0 |  | 28.6 | 71.4 | 0.0 | 0.0 |  | 1.3571 | -2.6733 | -1.3969 |  | 2.000E-02 | 1.012E-02 | 7.367E-02 |
| 676 | Q96AQ6 | PBXIP1 | Pre-B-cell leukemia transcription factor-interacting protein 1 | 731 |  | 6 | 15 | 0 | 0 |  | 28.6 | 71.4 | 0.0 | 0.0 |  | 1.3213 | -2.3217 | -1.0453 |  | 5.024E-02 | 3.698E-02 | 1.626E-01 |
| 677 | P20810 | CAST | Calpastatin | 708 |  | 2 | 5 | 0 | 0 |  | 28.6 | 71.4 | 0.0 | 0.0 |  | 1.0999 | -1.1640 | 0.1125 |  | 3.836E-01 | 5.166E-01 | 8.264E-01 |
| 678 | P25705 | ATP5A1 | ATP synthase subunit alpha, mitochondrial | 553 |  | 128 | 129 | 137 | 58 |  | 28.3 | 28.5 | 30.3 | 12.8 |  | 0.1684 | 0.3137 | 0.3680 |  | 5.015E-01 | 1.680E-01 | 8.539E-01 |
| 679 | Q92945 | KHSRP | Far upstream element-binding protein 2 | 711 |  | 15 | 23 | 15 | 0 |  | 28.3 | 43.4 | 28.3 | 0.0 |  | 0.7345 | 0.2148 | -2.2102 |  | 1.625E-01 | 9.088E-01 | 4.748E-03 |
| 680 | Q9NQC3 | RTN4 | Reticulon-4 | 1192 |  | 9 | 9 | 7 | 7 |  | 28.1 | 28.1 | 21.9 | 21.9 |  | 0.1564 | -0.0985 | 1.1786 |  | 9.534E-01 | 9.735E-01 | 4.727E-01 |
| 681 | P13010 | XRCC5 | X-ray repair cross-complementing protein 5 | 732 |  | 14 | 19 | 17 | 0 |  | 28.0 | 38.0 | 34.0 | 0.0 |  | 0.5658 | 0.4741 | -2.1185 |  | 3.485E-01 | 5.082E-01 | 7.015E-03 |
| 682 | Q06323 | PSME1 | Proteasome activator complex subunit 1 | 249 |  | 19 | 19 | 20 | 10 |  | 27.9 | 27.9 | 29.4 | 14.7 |  | 0.1564 | 0.2845 | 0.6437 |  | 9.239E-01 | 7.346E-01 | 9.376E-01 |
| 683 | Q15582 | TGFBI | Transforming growth factor-beta-induced protein ig-h3 | 683 |  | 36 | 36 | 47 | 10 |  | 27.9 | 27.9 | 36.4 | 7.8 |  | 0.1565 | 0.5891 | -0.2367 |  | 8.125E-01 | 1.109E-01 | 1.654E-01 |
| 684 | P50995 | ANXA11 | Annexin A11 | 505 |  | 16 | 19 | 21 | 2 |  | 27.6 | 32.8 | 36.2 | 3.4 |  | 0.3879 | 0.5824 | -0.9176 |  | 5.580E-01 | 3.229E-01 | 7.491E-02 |
| 685 | P60660 | MYL6 | Myosin light polypeptide 6 | 151 |  | 62 | 61 | 59 | 44 |  | 27.4 | 27.0 | 26.1 | 19.5 |  | 0.1337 | 0.1450 | 1.0117 |  | 7.766E-01 | 8.155E-01 | 5.258E-02 |
| 686 | P40199 | CEACAM6 | Carcinoembryonic antigen-related cell adhesion molecule 6 | 344 |  | 26 | 56 | 13 | 0 |  | 27.4 | 58.9 | 13.7 | 0.0 |  | 1.2295 | -0.7213 | -2.9567 |  | 2.997E-04 | 1.091E-01 | 6.569E-05 |
| 687 | P05155 | SERPING1 | Plasma protease C1 inhibitor | 500 |  | 3 | 5 | 0 | 3 |  | 27.3 | 45.5 | 0.0 | 27.3 |  | 0.7128 | -1.5510 | 1.4915 |  | 6.368E-01 | 2.641E-01 | 6.225E-01 |
| 688 | P61204 | ARF3 | ADP-ribosylation factor 3 | 181 |  | 33 | 32 | 38 | 18 |  | 27.3 | 26.4 | 31.4 | 14.9 |  | 0.1137 | 0.4119 | 0.6610 |  | 9.290E-01 | 3.510E-01 | 7.338E-01 |
| 689 | P40121 | CAPG | Macrophage-capping protein | 348 |  | 33 | 36 | 47 | 6 |  | 27.0 | 29.5 | 38.5 | 4.9 |  | 0.2779 | 0.7104 | -0.7499 |  | 5.561E-01 | 5.203E-02 | 2.871E-02 |
| 690 | P60866 | RPS20 | 40S ribosomal protein S20 | 142 |  | 10 | 11 | 14 | 2 |  | 27.0 | 29.7 | 37.8 | 5.4 |  | 0.2793 | 0.6539 | -0.3005 |  | 8.480E-01 | 3.794E-01 | 3.855E-01 |
| 691 | P01876 | IGHA1 | Ig alpha-1 chain C region | 353 |  | 42 | 41 | 33 | 40 |  | 26.9 | 26.3 | 21.2 | 25.6 |  | 0.1228 | -0.1222 | 1.4271 |  | 8.717E-01 | 6.485E-01 | 2.480E-03 |
| 692 | P28838 | LAP3 | Cytosol aminopeptidase | 519 |  | 7 | 11 | 3 | 5 |  | 26.9 | 42.3 | 11.5 | 19.2 |  | 0.7269 | -0.7425 | 1.0910 |  | 3.756E-01 | 4.318E-01 | 6.914E-01 |
| 693 | P04083 | ANXA1 | Annexin A1 | 346 |  | 91 | 81 | 87 | 80 |  | 26.8 | 23.9 | 25.7 | 23.6 |  | -0.0093 | 0.1513 | 1.3161 |  | 8.899E-01 | 7.266E-01 | 8.571E-05 |
| 694 | P62805 | HIST1H4A | histone H4;NA;cat=NA;ch=6 | 103 |  | 110 | 108 | 108 | 84 |  | 26.8 | 26.3 | 26.3 | 20.5 |  | 0.1307 | 0.1894 | 1.1148 |  | 6.780E-01 | 5.490E-01 | 1.369E-03 |
| 695 | P12814 | ACTN1 | Alpha-actinin-1 | 914 |  | 94 | 122 | 135 | 0 |  | 26.8 | 34.8 | 38.5 | 0.0 |  | 0.5306 | 0.7349 | -4.7663 |  | 1.393E-02 | 5.053E-04 | 2.275E-16 |
| 696 | P30041 | PRDX6 | Peroxiredoxin-6 | 224 |  | 62 | 63 | 65 | 43 |  | 26.6 | 27.0 | 27.9 | 18.5 |  | 0.1794 | 0.2823 | 0.9793 |  | 6.397E-01 | 4.295E-01 | 7.026E-02 |
| 697 | P06703 | S100A6 | Protein S100-A6 | 90 |  | 60 | 83 | 54 | 30 |  | 26.4 | 36.6 | 23.8 | 13.2 |  | 0.6182 | 0.0661 | 0.5218 |  | 1.897E-02 | 9.387E-01 | 8.872E-01 |
| 698 | Q14103 | HNRNPD | Heterogeneous nuclear ribonucleoprotein D0 | 355 |  | 28 | 31 | 33 | 14 |  | 26.4 | 29.2 | 31.1 | 13.2 |  | 0.2975 | 0.4430 | 0.5522 |  | 5.606E-01 | 3.489E-01 | 9.929E-01 |
| 699 | P61224 | RAP1B | Ras-related protein Rap-1b | 184 |  | 15 | 23 | 19 | 0 |  | 26.3 | 40.4 | 33.3 | 0.0 |  | 0.7345 | 0.5326 | -2.2102 |  | 1.625E-01 | 4.057E-01 | 4.748E-03 |
| 700 | P07737 | PFN1 | Profilin-1 | 140 |  | 57 | 58 | 70 | 33 |  | 26.1 | 26.6 | 32.1 | 15.1 |  | 0.1813 | 0.5068 | 0.7271 |  | 6.534E-01 | 9.313E-02 | 4.167E-01 |
| 701 | O43143 | DHX15 | Putative pre-mRNA-splicing factor ATP-dependent RNA helicase DHX15 | 813 |  | 14 | 25 | 15 | 0 |  | 25.9 | 46.3 | 27.8 | 0.0 |  | 0.9406 | 0.3065 | -2.1185 |  | 6.135E-02 | 7.701E-01 | 7.015E-03 |
| 702 | P02787 | TF | Serotransferrin | 698 |  | 36 | 19 | 31 | 53 |  | 25.9 | 13.7 | 22.3 | 38.1 |  | -0.7239 | 0.0067 | 2.0404 |  | 6.196E-02 | 9.641E-01 | 1.833E-07 |
| 703 | Q15717 | ELAVL1 | ELAV-like protein 1 | 326 |  | 9 | 16 | 10 | 0 |  | 25.7 | 45.7 | 28.6 | 0.0 |  | 0.9078 | 0.3491 | -1.5450 |  | 1.572E-01 | 8.130E-01 | 4.970E-02 |
| 704 | P09525 | ANXA4 | Annexin A4 | 321 |  | 80 | 100 | 112 | 20 |  | 25.6 | 32.1 | 35.9 | 6.4 |  | 0.4757 | 0.6968 | -0.4452 |  | 4.749E-02 | 2.710E-03 | 9.715E-03 |
| 705 | P23142 | FBLN1 | Fibulin-1 | 703 |  | 11 | 9 | 23 | 0 |  | 25.6 | 20.9 | 53.5 | 0.0 |  | -0.1009 | 1.2008 | -1.8023 |  | 9.707E-01 | 2.653E-02 | 2.267E-02 |
| 706 | Q03252 | LMNB2 | Lamin-B2 | 600 |  | 43 | 45 | 51 | 30 |  | 25.4 | 26.6 | 30.2 | 17.8 |  | 0.2205 | 0.4554 | 0.9918 |  | 6.175E-01 | 2.115E-01 | 1.344E-01 |
| 707 | O43707 | ACTN4 | Alpha-actinin-4 | 911 |  | 148 | 182 | 195 | 65 |  | 25.1 | 30.8 | 33.1 | 11.0 |  | 0.4557 | 0.6144 | 0.3215 |  | 9.703E-03 | 4.584E-04 | 6.760E-01 |
| 708 | P59665 | DEFA1 | neutrophil defensin 1 precursor;NA;cat | 94 |  | 18 | 18 | 18 | 18 |  | 25.0 | 25.0 | 25.0 | 25.0 |  | 0.1564 | 0.2148 | 1.4931 |  | 9.329E-01 | 8.738E-01 | 3.967E-02 |
| 709 | O75368 | SH3BGRL | SH3 domain-binding glutamic acid-rich-like protein | 114 |  | 12 | 16 | 15 | 5 |  | 25.0 | 33.3 | 31.3 | 10.4 |  | 0.5372 | 0.5094 | 0.4072 |  | 4.302E-01 | 5.047E-01 | 9.807E-01 |
| 710 | Q9NVD7 | PARVA | Alpha-parvin | 372 |  | 5 | 8 | 6 | 1 |  | 25.0 | 40.0 | 30.0 | 5.0 |  | 0.7221 | 0.4289 | 0.0171 |  | 4.799E-01 | 8.572E-01 | 7.040E-01 |
| 711 | Q14019 | COTL1 | Coactosin-like protein | 142 |  | 3 | 6 | 3 | 0 |  | 25.0 | 50.0 | 25.0 | 0.0 |  | 0.9270 | 0.2147 | -0.2746 |  | 4.269E-01 | 7.820E-01 | 5.442E-01 |
| 712 | Q96EP5 | DAZAP1 | DAZ-associated protein 1 | 407 |  | 2 | 4 | 2 | 0 |  | 25.0 | 50.0 | 25.0 | 0.0 |  | 0.8483 | 0.2147 | 0.1125 |  | 6.090E-01 | 6.929E-01 | 8.264E-01 |
| 713 | O95436 | SLC34A2 | Sodium-dependent phosphate transport protein 2B | 689 |  | 3 | 9 | 0 | 0 |  | 25.0 | 75.0 | 0.0 | 0.0 |  | 1.4268 | -1.5510 | -0.2746 |  | 1.077E-01 | 2.641E-01 | 5.442E-01 |
| 714 | Q14728 | MFSD10 | Major facilitator superfamily domain-containing protein 10 | 455 |  | 2 | 6 | 0 | 0 |  | 25.0 | 75.0 | 0.0 | 0.0 |  | 1.3141 | -1.1640 | 0.1125 |  | 2.337E-01 | 5.166E-01 | 8.264E-01 |
| 715 | O75506 | HSBP1 | Heat shock factor-binding protein 1 | 76 |  | 1 | 3 | 0 | 0 |  | 25.0 | 75.0 | 0.0 | 0.0 |  | 1.0740 | -0.6334 | 0.6431 |  | 5.579E-01 | 9.567E-01 | 7.108E-01 |
| 716 | P24821 | TNC | Tenascin | 2201 |  | 70 | 90 | 121 | 0 |  | 24.9 | 32.0 | 43.1 | 0.0 |  | 0.5151 | 0.9978 | -4.3460 |  | 4.177E-02 | 1.112E-05 | 2.524E-12 |
| 717 | HIP000300882 | HIP000300882 | Tropomyosin family protein;NA;cat | 239 |  | 54 | 63 | 68 | 33 |  | 24.8 | 28.9 | 31.2 | 15.1 |  | 0.3750 | 0.5420 | 0.8036 |  | 2.326E-01 | 7.518E-02 | 2.974E-01 |
| 718 | P30086 | PEBP1 | Phosphatidylethanolamine-binding protein 1 | 187 |  | 30 | 39 | 40 | 13 |  | 24.6 | 32.0 | 32.8 | 10.7 |  | 0.5222 | 0.6162 | 0.3586 |  | 1.894E-01 | 1.255E-01 | 9.233E-01 |
| 719 | P35579 | MYH9 | Myosin-9 | 1960 |  | 459 | 577 | 586 | 250 |  | 24.5 | 30.8 | 31.3 | 13.4 |  | 0.4969 | 0.5799 | 0.6329 |  | 4.704E-07 | 1.079E-08 | 7.544E-02 |
| 720 | P21291 | CSRP1 | Cysteine and glycine-rich protein 1 | 193 |  | 11 | 14 | 11 | 9 |  | 24.4 | 31.1 | 24.4 | 20.0 |  | 0.4726 | 0.2148 | 1.2348 |  | 5.422E-01 | 9.672E-01 | 3.293E-01 |
| 721 | O75369 | FLNB | Filamin-B | 2602 |  | 31 | 49 | 48 | 0 |  | 24.2 | 38.3 | 37.5 | 0.0 |  | 0.7975 | 0.8270 | -3.2000 |  | 2.245E-02 | 2.206E-02 | 9.421E-06 |
| 722 | Q16762 | TST | Thiosulfate sulfurtransferase | 297 |  | 15 | 22 | 25 | 0 |  | 24.2 | 35.5 | 40.3 | 0.0 |  | 0.6737 | 0.9074 | -2.2102 |  | 2.139E-01 | 7.641E-02 | 4.748E-03 |
| 723 | P13489 | RNH1 | Ribonuclease inhibitor | 461 |  | 14 | 15 | 20 | 9 |  | 24.1 | 25.9 | 34.5 | 15.5 |  | 0.2481 | 0.6939 | 0.9186 |  | 8.218E-01 | 2.398E-01 | 6.205E-01 |
| 724 | P09382 | LGALS1 | Galectin-1 | 135 |  | 7 | 13 | 9 | 0 |  | 24.1 | 44.8 | 31.0 | 0.0 |  | 0.9452 | 0.5280 | -1.2318 |  | 1.900E-01 | 6.404E-01 | 1.093E-01 |
| 725 | Q7Z406 | MYH14 | Myosin-14 | 2036 |  | 83 | 128 | 96 | 37 |  | 24.1 | 37.2 | 27.9 | 10.8 |  | 0.7773 | 0.4234 | 0.3532 |  | 2.584E-04 | 1.039E-01 | 8.451E-01 |
| 726 | P02545 | LMNA | Prelamin-A/C | 664 |  | 248 | 338 | 297 | 157 |  | 23.8 | 32.5 | 28.6 | 15.1 |  | 0.6089 | 0.4795 | 0.8476 |  | 2.322E-06 | 8.295E-04 | 5.100E-03 |
| 727 | P62158 | CALM1 | Calmodulin | 149 |  | 35 | 37 | 40 | 35 |  | 23.8 | 25.2 | 27.2 | 23.8 |  | 0.2341 | 0.4018 | 1.4950 |  | 6.362E-01 | 3.510E-01 | 2.841E-03 |
| 728 | P02751 | FN1 | Fibronectin | 1073 |  | 71 | 105 | 92 | 31 |  | 23.7 | 35.1 | 30.8 | 10.4 |  | 0.7155 | 0.5849 | 0.3284 |  | 2.259E-03 | 2.354E-02 | 7.947E-01 |
| 729 | P14866 | HNRNPL | Heterogeneous nuclear ribonucleoprotein L | 589 |  | 9 | 12 | 9 | 8 |  | 23.7 | 31.6 | 23.7 | 21.1 |  | 0.5269 | 0.2148 | 1.3439 |  | 5.297E-01 | 9.955E-01 | 3.002E-01 |
| 730 | P01834 | IGKC | Ig kappa chain C region | 106 |  | 37 | 26 | 34 | 60 |  | 23.6 | 16.6 | 21.7 | 38.2 |  | -0.3334 | 0.0970 | 2.1784 |  | 3.513E-01 | 9.857E-01 | 3.343E-09 |
| 731 | P00568 | AK1 | Adenylate kinase isoenzyme 1 | 194 |  | 45 | 72 | 61 | 13 |  | 23.6 | 37.7 | 31.9 | 6.8 |  | 0.8218 | 0.6448 | -0.2079 |  | 4.130E-03 | 4.342E-02 | 1.390E-01 |
| 732 | P11216 | PYGB | Glycogen phosphorylase, brain form | 843 |  | 26 | 48 | 38 | 0 |  | 23.2 | 42.9 | 33.9 | 0.0 |  | 1.0118 | 0.7422 | -2.9567 |  | 4.835E-03 | 6.984E-02 | 6.569E-05 |
| 733 | P0CG05 | IGLC2 | Ig lambda-2 chain C regions | 106 |  | 16 | 14 | 19 | 20 |  | 23.2 | 20.3 | 27.5 | 29.0 |  | -0.0215 | 0.4464 | 1.7944 |  | 9.630E-01 | 5.097E-01 | 6.314E-03 |
| 734 | P24752 | ACAT1 | Acetyl-CoA acetyltransferase, mitochondrial | 427 |  | 24 | 33 | 45 | 2 |  | 23.1 | 31.7 | 43.3 | 1.9 |  | 0.5969 | 1.0895 | -1.4677 |  | 1.683E-01 | 4.038E-03 | 6.488E-03 |
| 735 | Q15019 | SEPT2 | Septin-2 | 361 |  | 12 | 17 | 23 | 0 |  | 23.1 | 32.7 | 44.2 | 0.0 |  | 0.6186 | 1.0875 | -1.9156 |  | 3.324E-01 | 4.317E-02 | 1.533E-02 |
| 736 | Q01105 | SET | Protein SET | 290 |  | 12 | 23 | 17 | 0 |  | 23.1 | 44.2 | 32.7 | 0.0 |  | 1.0291 | 0.6770 | -1.9156 |  | 5.132E-02 | 3.001E-01 | 1.533E-02 |
| 737 | P07355 | ANXA2 | Annexin A2 | 339 |  | 135 | 142 | 157 | 152 |  | 23.0 | 24.2 | 26.8 | 25.9 |  | 0.2300 | 0.4335 | 1.6786 |  | 2.893E-01 | 3.053E-02 | 3.704E-13 |
| 738 | P08670 | VIM | Vimentin | 466 |  | 293 | 329 | 369 | 283 |  | 23.0 | 25.8 | 29.0 | 22.2 |  | 0.3273 | 0.5544 | 1.4723 |  | 1.380E-02 | 1.403E-05 | 1.802E-17 |
| 739 | Q96QK1 | VPS35 | Vacuolar protein sorting-associated protein 35 | 796 |  | 7 | 11 | 10 | 3 |  | 22.6 | 35.5 | 32.3 | 9.7 |  | 0.7269 | 0.6624 | 0.5343 |  | 3.756E-01 | 4.781E-01 | 8.443E-01 |
| 740 | Q9Y6C2 | EMILIN1 | EMILIN-1 | 1016 |  | 43 | 41 | 61 | 46 |  | 22.5 | 21.5 | 31.9 | 24.1 |  | 0.0897 | 0.7087 | 1.5910 |  | 9.554E-01 | 2.604E-02 | 2.014E-04 |
| 741 | P21397 | MAOA | Amine oxidase [flavin-containing] A | 527 |  | 20 | 35 | 24 | 10 |  | 22.5 | 39.3 | 27.0 | 11.2 |  | 0.9280 | 0.4640 | 0.5741 |  | 2.723E-02 | 4.164E-01 | 9.613E-01 |
| 742 | P02647 | APOA1 | Apolipoprotein A-I | 267 |  | 50 | 35 | 57 | 83 |  | 22.2 | 15.6 | 25.3 | 36.9 |  | -0.3439 | 0.4004 | 2.2194 |  | 2.549E-01 | 2.532E-01 | 1.345E-12 |
| 743 | P04004 | VTN | Vitronectin | 478 |  | 28 | 36 | 17 | 45 |  | 22.2 | 28.6 | 13.5 | 35.7 |  | 0.5058 | -0.4664 | 2.1581 |  | 2.259E-01 | 2.562E-01 | 4.136E-07 |
| 744 | P63241 | EIF5A | Eukaryotic translation initiation factor 5A-1 | 184 |  | 10 | 11 | 15 | 9 |  | 22.2 | 24.4 | 33.3 | 20.0 |  | 0.2793 | 0.7456 | 1.3578 |  | 8.480E-01 | 2.833E-01 | 2.499E-01 |
| 745 | P07988 | SFTPB | Pulmonary surfactant-associated protein B | 381 |  | 28 | 44 | 39 | 15 |  | 22.2 | 34.9 | 31.0 | 11.9 |  | 0.7870 | 0.6763 | 0.6440 |  | 3.320E-02 | 9.517E-02 | 8.183E-01 |
| 746 | Q01518 | CAP1 | Adenylyl cyclase-associated protein 1 | 475 |  | 18 | 32 | 23 | 8 |  | 22.2 | 39.5 | 28.4 | 9.9 |  | 0.9459 | 0.5483 | 0.4341 |  | 3.199E-02 | 3.306E-01 | 9.467E-01 |
| 747 | O00264 | PGRMC1 | Membrane-associated progesterone receptor component 1 | 195 |  | 2 | 0 | 7 | 0 |  | 22.2 | 0.0 | 77.8 | 0.0 |  | -1.2223 | 1.5590 | 0.1125 |  | 5.056E-01 | 1.292E-01 | 8.264E-01 |
| 748 | Q13751 | LAMB3 | Laminin subunit beta-3 | 1172 |  | 2 | 7 | 0 | 0 |  | 22.2 | 77.8 | 0.0 | 0.0 |  | 1.5006 | -1.1640 | 0.1125 |  | 1.387E-01 | 5.166E-01 | 8.264E-01 |
| 749 | P02743 | APCS | Serum amyloid P-component | 223 |  | 60 | 61 | 67 | 83 |  | 22.1 | 22.5 | 24.7 | 30.6 |  | 0.1801 | 0.3719 | 1.9616 |  | 6.451E-01 | 2.520E-01 | 2.918E-10 |
| 750 | P17844 | DDX5 | Probable ATP-dependent RNA helicase DDX5 | 614 |  | 15 | 32 | 17 | 4 |  | 22.1 | 47.1 | 25.0 | 5.9 |  | 1.1905 | 0.3824 | -0.1391 |  | 8.222E-03 | 6.285E-01 | 4.204E-01 |
| 751 | Q13228 | SELENBP1 | Selenium-binding protein 1 | 472 |  | 73 | 92 | 132 | 34 |  | 22.1 | 27.8 | 39.9 | 10.3 |  | 0.4868 | 1.0632 | 0.4177 |  | 5.203E-02 | 1.165E-06 | 9.710E-01 |
| 752 | P10909 | CLU | Clusterin | 449 |  | 26 | 26 | 22 | 44 |  | 22.0 | 22.0 | 18.6 | 37.3 |  | 0.1565 | -0.0144 | 2.2287 |  | 8.706E-01 | 9.541E-01 | 2.500E-07 |
| 753 | P00387 | CYB5R3 | NADH-cytochrome b5 reductase 3 | 301 |  | 11 | 11 | 23 | 5 |  | 22.0 | 22.0 | 46.0 | 10.0 |  | 0.1564 | 1.2008 | 0.5205 |  | 9.863E-01 | 2.653E-02 | 8.449E-01 |
| 754 | P0CG47 | UBB | Polyubiquitin-B | 229 |  | 25 | 31 | 30 | 29 |  | 21.7 | 27.0 | 26.1 | 25.2 |  | 0.4538 | 0.4668 | 1.6992 |  | 3.261E-01 | 3.460E-01 | 1.692E-03 |
| 755 | P00738 | HP | Haptoglobin | 406 |  | 8 | 1 | 5 | 23 |  | 21.6 | 2.7 | 13.5 | 62.2 |  | -1.8837 | -0.3511 | 2.8850 |  | 4.883E-02 | 7.183E-01 | 3.844E-06 |
| 756 | P25325 | MPST | 3-mercaptopyruvate sulfurtransferase | 297 |  | 3 | 6 | 5 | 0 |  | 21.4 | 42.9 | 35.7 | 0.0 |  | 0.9270 | 0.7712 | -0.2746 |  | 4.269E-01 | 6.119E-01 | 5.442E-01 |
| 757 | P09493 | TPM1 | Tropomyosin alpha-1 chain | 284 |  | 57 | 75 | 94 | 41 |  | 21.3 | 28.1 | 35.2 | 15.4 |  | 0.5464 | 0.9273 | 1.0314 |  | 4.948E-02 | 3.019E-04 | 5.453E-02 |
| 758 | P04899 | GNAI2 | Guanine nucleotide-binding protein G(i) subunit alpha-2 | 355 |  | 10 | 12 | 14 | 11 |  | 21.3 | 25.5 | 29.8 | 23.4 |  | 0.3926 | 0.6539 | 1.6153 |  | 6.822E-01 | 3.794E-01 | 8.918E-02 |
| 759 | P13796 | LCP1 | Plastin-2 | 627 |  | 33 | 47 | 57 | 19 |  | 21.2 | 30.1 | 36.5 | 12.2 |  | 0.6519 | 0.9828 | 0.7342 |  | 6.701E-02 | 3.146E-03 | 5.920E-01 |
| 760 | P17931 | LGALS3 | Galectin-3 | 250 |  | 12 | 15 | 19 | 11 |  | 21.1 | 26.3 | 33.3 | 19.3 |  | 0.4510 | 0.8272 | 1.3791 |  | 5.468E-01 | 1.667E-01 | 1.752E-01 |
| 761 | P08123 | COL1A2 | Collagen alpha-2(I) chain | 1366 |  | 75 | 84 | 92 | 115 |  | 20.5 | 23.0 | 25.1 | 31.4 |  | 0.3184 | 0.5070 | 2.1147 |  | 2.445E-01 | 5.197E-02 | 1.380E-15 |
| 762 | P02461 | COL3A1 | Collagen alpha-1(III) chain | 1466 |  | 14 | 16 | 23 | 16 |  | 20.3 | 23.2 | 33.3 | 23.2 |  | 0.3343 | 0.8846 | 1.6708 |  | 6.805E-01 | 9.892E-02 | 2.666E-02 |
| 763 | P20231 | TPSB2 | Tryptase beta-2 | 275 |  | 18 | 29 | 42 | 0 |  | 20.2 | 32.6 | 47.2 | 0.0 |  | 0.8092 | 1.3844 | -2.4548 |  | 7.996E-02 | 6.137E-04 | 1.475E-03 |
| 764 | P35749 | MYH11 | Myosin-11 | 1945 |  | 103 | 180 | 229 | 0 |  | 20.1 | 35.2 | 44.7 | 0.0 |  | 0.9602 | 1.3677 | -4.8971 |  | 1.435E-07 | 1.851E-15 | 6.911E-18 |
| 765 | Q96HC4 | PDLIM5 | PDZ and LIM domain protein 5 | 487 |  | 1 | 0 | 4 | 0 |  | 20.0 | 0.0 | 80.0 | 0.0 |  | -0.6918 | 1.4373 | 0.6431 |  | 9.666E-01 | 3.044E-01 | 7.108E-01 |
| 766 | Q15165 | PON2 | Serum paraoxonase/arylesterase 2 | 354 |  | 3 | 6 | 6 | 0 |  | 20.0 | 40.0 | 40.0 | 0.0 |  | 0.9270 | 0.9854 | -0.2746 |  | 4.269E-01 | 4.055E-01 | 5.442E-01 |
| 767 | Q9NR45 | NANS | Sialic acid synthase | 359 |  | 2 | 4 | 4 | 0 |  | 20.0 | 40.0 | 40.0 | 0.0 |  | 0.8483 | 0.9067 | 0.1125 |  | 6.090E-01 | 5.879E-01 | 8.264E-01 |
| 768 | Q99426 | TBCB | Tubulin-folding cofactor B | 244 |  | 1 | 2 | 2 | 0 |  | 20.0 | 40.0 | 40.0 | 0.0 |  | 0.6869 | 0.7453 | 0.6431 |  | 9.423E-01 | 9.251E-01 | 7.108E-01 |
| 769 | P10599 | TXN | Thioredoxin | 105 |  | 5 | 12 | 8 | 0 |  | 20.0 | 48.0 | 32.0 | 0.0 |  | 1.2409 | 0.7805 | -0.8311 |  | 1.006E-01 | 4.525E-01 | 2.422E-01 |
| 770 | P53396 | ACLY | ATP-citrate synthase | 1101 |  | 1 | 4 | 0 | 0 |  | 20.0 | 80.0 | 0.0 | 0.0 |  | 1.3789 | -0.6334 | 0.6431 |  | 3.176E-01 | 9.567E-01 | 7.108E-01 |
| 771 | P08133 | ANXA6 | Annexin A6 | 673 |  | 52 | 70 | 73 | 68 |  | 19.8 | 26.6 | 27.8 | 25.9 |  | 0.5780 | 0.6963 | 1.8786 |  | 4.491E-02 | 1.632E-02 | 4.953E-08 |
| 772 | P02452 | COL1A1 | Collagen alpha-1(I) chain | 1464 |  | 93 | 112 | 129 | 140 |  | 19.6 | 23.6 | 27.2 | 29.5 |  | 0.4232 | 0.6848 | 2.0932 |  | 6.282E-02 | 1.532E-03 | 2.559E-18 |
| 773 | P50895 | BCAM | Basal cell adhesion molecule | 628 |  | 10 | 15 | 3 | 23 |  | 19.6 | 29.4 | 5.9 | 45.1 |  | 0.6872 | -1.1901 | 2.6025 |  | 3.121E-01 | 1.324E-01 | 2.184E-05 |
| 774 | Q14894 | CRYM | Thiomorpholine-carboxylate dehydrogenase | 314 |  | 10 | 14 | 27 | 0 |  | 19.6 | 27.5 | 52.9 | 0.0 |  | 0.5955 | 1.5443 | -1.6794 |  | 4.133E-01 | 2.693E-03 | 3.355E-02 |
| 775 | Q16555 | DPYSL2 | Dihydropyrimidinase-related protein 2 | 572 |  | 60 | 78 | 111 | 61 |  | 19.4 | 25.2 | 35.8 | 19.7 |  | 0.5296 | 1.0928 | 1.5213 |  | 5.206E-02 | 4.994E-06 | 4.390E-05 |
| 776 | P23526 | AHCY | Adenosylhomocysteinase | 432 |  | 6 | 8 | 17 | 0 |  | 19.4 | 25.8 | 54.8 | 0.0 |  | 0.5079 | 1.5473 | -1.0453 |  | 6.716E-01 | 1.743E-02 | 1.626E-01 |
| 777 | P32119 | PRDX2 | Peroxiredoxin-2 | 198 |  | 45 | 57 | 58 | 73 |  | 19.3 | 24.5 | 24.9 | 31.3 |  | 0.4902 | 0.5734 | 2.1838 |  | 1.300E-01 | 8.258E-02 | 5.882E-11 |
| 778 | P21333 | FLNA | Filamin-A | 2647 |  | 215 | 369 | 429 | 102 |  | 19.3 | 33.1 | 38.5 | 9.1 |  | 0.9441 | 1.2244 | 0.4290 |  | 1.186E-13 | 3.963E-23 | 9.816E-01 |
| 779 | P35580 | MYH10 | Myosin-10 | 1976 |  | 115 | 185 | 198 | 102 |  | 19.2 | 30.8 | 33.0 | 17.0 |  | 0.8418 | 0.9990 | 1.3305 |  | 2.160E-06 | 1.742E-08 | 6.401E-06 |
| 780 | P54819 | AK2 | Adenylate kinase 2, mitochondrial | 232 |  | 10 | 22 | 21 | 0 |  | 18.9 | 41.5 | 39.6 | 0.0 |  | 1.2045 | 1.1994 | -1.6794 |  | 2.783E-02 | 3.462E-02 | 3.355E-02 |
| 781 | P15502 | ELN | Elastin | 786 |  | 90 | 163 | 132 | 93 |  | 18.8 | 34.1 | 27.6 | 19.5 |  | 1.0099 | 0.7647 | 1.5482 |  | 1.669E-07 | 3.507E-04 | 2.227E-07 |
| 782 | P35625 | TIMP3 | Metalloproteinase inhibitor 3 | 211 |  | 25 | 38 | 22 | 48 |  | 18.8 | 28.6 | 16.5 | 36.1 |  | 0.7377 | 0.0396 | 2.4056 |  | 6.448E-02 | 9.362E-01 | 7.714E-09 |
| 783 | Q99729 | HNRNPAB | Heterogeneous nuclear ribonucleoprotein A/B | 332 |  | 3 | 5 | 8 | 0 |  | 18.8 | 31.3 | 50.0 | 0.0 |  | 0.7128 | 1.3370 | -0.2746 |  | 6.368E-01 | 1.620E-01 | 5.442E-01 |
| 784 | P21980 | TGM2 | Protein-glutamine gamma-glutamyltransferase 2 | 687 |  | 43 | 46 | 74 | 68 |  | 18.6 | 19.9 | 32.0 | 29.4 |  | 0.2514 | 0.9833 | 2.1463 |  | 5.438E-01 | 7.316E-04 | 5.146E-10 |
| 785 | P04179 | SOD2 | Superoxide dismutase [Mn], mitochondrial | 222 |  | 10 | 14 | 30 | 0 |  | 18.5 | 25.9 | 55.6 | 0.0 |  | 0.5955 | 1.6901 | -1.6794 |  | 4.133E-01 | 6.635E-04 | 3.355E-02 |
| 786 | Q9Y490 | TLN1 | Talin-1 | 2541 |  | 49 | 84 | 84 | 48 |  | 18.5 | 31.7 | 31.7 | 18.1 |  | 0.9216 | 0.9802 | 1.4673 |  | 5.619E-04 | 3.275E-04 | 5.580E-04 |
| 787 | P51884 | LUM | Lumican | 338 |  | 24 | 36 | 42 | 29 |  | 18.3 | 27.5 | 32.1 | 22.1 |  | 0.7182 | 0.9926 | 1.7553 |  | 8.039E-02 | 1.093E-02 | 1.127E-03 |
| 788 | Q12905 | ILF2 | Interleukin enhancer-binding factor 2 | 390 |  | 8 | 21 | 15 | 0 |  | 18.2 | 47.7 | 34.1 | 0.0 |  | 1.4235 | 1.0282 | -1.3969 |  | 1.295E-02 | 1.291E-01 | 7.367E-02 |
| 789 | P01620 | IGKV1-5 | Ig kappa chain V-III region SIE | 109 |  | 5 | 4 | 7 | 12 |  | 17.9 | 14.3 | 25.0 | 42.9 |  | -0.0953 | 0.6154 | 2.5769 |  | 9.001E-01 | 6.335E-01 | 2.690E-03 |
| 790 | P12429 | ANXA3 | Annexin A3 | 323 |  | 37 | 49 | 74 | 49 |  | 17.7 | 23.4 | 35.4 | 23.4 |  | 0.5510 | 1.1939 | 1.8909 |  | 1.149E-01 | 5.819E-05 | 3.464E-06 |
| 791 | O75955 | FLOT1 | Flotillin-1 | 427 |  | 3 | 7 | 7 | 0 |  | 17.6 | 41.2 | 41.2 | 0.0 |  | 1.1135 | 1.1719 | -0.2746 |  | 2.768E-01 | 2.599E-01 | 5.442E-01 |
| 792 | P12111 | COL6A3 | Collagen alpha-3(VI) chain | 1036 |  | 135 | 176 | 222 | 244 |  | 17.4 | 22.7 | 28.6 | 31.4 |  | 0.5395 | 0.9345 | 2.3730 |  | 2.546E-03 | 1.953E-08 | 5.048E-38 |
| 793 | P12109 | COL6A1 | Collagen alpha-1(VI) chain | 1028 |  | 63 | 90 | 93 | 121 |  | 17.2 | 24.5 | 25.3 | 33.0 |  | 0.6647 | 0.7702 | 2.4361 |  | 8.600E-03 | 2.614E-03 | 1.415E-20 |
| 794 | P04233 | CD74 | HLA class II histocompatibility antigen gamma chain | 296 |  | 6 | 12 | 17 | 0 |  | 17.1 | 34.3 | 48.6 | 0.0 |  | 1.0267 | 1.5473 | -1.0453 |  | 1.729E-01 | 1.743E-02 | 1.626E-01 |
| 795 | P27658 | COL8A1 | Collagen alpha-1(VIII) chain | 744 |  | 23 | 38 | 47 | 27 |  | 17.0 | 28.1 | 34.8 | 20.0 |  | 0.8522 | 1.2091 | 1.7146 |  | 3.340E-02 | 1.249E-03 | 2.268E-03 |
| 796 | P12110 | COL6A2 | Collagen alpha-2(VI) chain | 1019 |  | 61 | 79 | 96 | 123 |  | 17.0 | 22.0 | 26.7 | 34.3 |  | 0.5244 | 0.8613 | 2.5056 |  | 5.290E-02 | 6.553E-04 | 7.969E-22 |
| 797 | P08572 | COL4A2 | Collagen alpha-2(IV) chain | 1042 |  | 10 | 16 | 16 | 17 |  | 16.9 | 27.1 | 27.1 | 28.8 |  | 0.7734 | 0.8319 | 2.1914 |  | 2.314E-01 | 2.077E-01 | 1.983E-03 |
| 798 | P98160 | HSPG2 | Basement membrane-specific heparan sulfate proteoglycan core protein | 4391 |  | 125 | 273 | 208 | 136 |  | 16.8 | 36.8 | 28.0 | 18.3 |  | 1.2864 | 0.9507 | 1.6274 |  | 9.125E-17 | 3.352E-08 | 3.115E-11 |
| 799 | P21810 | BGN | Biglycan | 368 |  | 19 | 23 | 29 | 42 |  | 16.8 | 20.4 | 25.7 | 37.2 |  | 0.4168 | 0.7946 | 2.5919 |  | 4.627E-01 | 9.318E-02 | 8.911E-09 |
| 800 | Q15063 | POSTN | Periostin | 836 |  | 30 | 58 | 80 | 11 |  | 16.8 | 32.4 | 44.7 | 6.1 |  | 1.0813 | 1.5970 | 0.1401 |  | 9.873E-04 | 1.029E-07 | 5.756E-01 |
| 801 | Q8WWI1 | LMO7 | LIM domain only protein 7 | 1557 |  | 3 | 14 | 0 | 1 |  | 16.7 | 77.8 | 0.0 | 5.6 |  | 2.0003 | -1.5510 | 0.5736 |  | 7.503E-03 | 2.641E-01 | 8.187E-01 |
| 802 | P78417 | GSTO1 | Glutathione S-transferase omega-1 | 241 |  | 2 | 6 | 4 | 0 |  | 16.7 | 50.0 | 33.3 | 0.0 |  | 1.3141 | 0.9067 | 0.1125 |  | 2.337E-01 | 5.879E-01 | 8.264E-01 |
| 803 | P52434 | POLR2H | DNA-directed RNA polymerases I, II, and III subunit RPABC3 | 150 |  | 1 | 5 | 0 | 0 |  | 16.7 | 83.3 | 0.0 | 0.0 |  | 1.6305 | -0.6334 | 0.6431 |  | 1.760E-01 | 9.567E-01 | 7.108E-01 |
| 804 | Q16853 | AOC3 | Membrane primary amine oxidase | 763 |  | 24 | 37 | 36 | 49 |  | 16.4 | 25.3 | 24.7 | 33.6 |  | 0.7565 | 0.7767 | 2.4909 |  | 6.170E-02 | 6.508E-02 | 1.820E-09 |
| 805 | O00151 | PDLIM1 | PDZ and LIM domain protein 1 | 329 |  | 10 | 23 | 23 | 5 |  | 16.4 | 37.7 | 37.7 | 8.2 |  | 1.2653 | 1.3237 | 0.6434 |  | 1.869E-02 | 1.541E-02 | 8.622E-01 |
| 806 | P18206 | VCL | Vinculin | 1066 |  | 64 | 105 | 149 | 80 |  | 16.1 | 26.4 | 37.4 | 20.1 |  | 0.8629 | 1.4246 | 1.8173 |  | 2.733E-04 | 3.319E-11 | 1.046E-08 |
| 807 | Q13813 | SPTAN1 | Spectrin alpha chain, brain | 2472 |  | 43 | 66 | 79 | 90 |  | 15.5 | 23.7 | 28.4 | 32.4 |  | 0.7620 | 1.0764 | 2.5480 |  | 1.069E-02 | 1.496E-04 | 8.426E-17 |
| 808 | P04264 | KRT1 | Keratin, type II cytoskeletal 1 | 644 |  | 111 | 202 | 213 | 194 |  | 15.4 | 28.1 | 29.6 | 26.9 |  | 1.0198 | 1.1555 | 2.3163 |  | 4.167E-09 | 2.924E-11 | 1.951E-29 |
| 809 | Q01130 | SFRS2 | Serine/arginine-rich splicing factor 2 | 221 |  | 2 | 4 | 7 | 0 |  | 15.4 | 30.8 | 53.8 | 0.0 |  | 0.8483 | 1.5590 | 0.1125 |  | 6.090E-01 | 1.292E-01 | 8.264E-01 |
| 810 | Q01995 | TAGLN | Transgelin | 201 |  | 41 | 101 | 108 | 18 |  | 15.3 | 37.7 | 40.3 | 6.7 |  | 1.4357 | 1.5904 | 0.3576 |  | 3.264E-08 | 7.104E-10 | 9.050E-01 |
| 811 | P00352 | ALDH1A1 | Retinal dehydrogenase 1 | 501 |  | 14 | 17 | 36 | 25 |  | 15.2 | 18.5 | 39.1 | 27.2 |  | 0.4157 | 1.5048 | 2.2780 |  | 5.536E-01 | 6.820E-04 | 8.363E-05 |
| 812 | Q05682 | CALD1 | Caldesmon | 563 |  | 12 | 35 | 34 | 0 |  | 14.8 | 43.2 | 42.0 | 0.0 |  | 1.6099 | 1.6280 | -1.9156 |  | 3.764E-04 | 4.345E-04 | 1.533E-02 |
| 813 | P51888 | PRELP | Prolargin | 382 |  | 16 | 34 | 34 | 33 |  | 13.7 | 29.1 | 29.1 | 28.2 |  | 1.1886 | 1.2471 | 2.4853 |  | 6.472E-03 | 4.951E-03 | 9.167E-07 |
| 814 | P35555 | FBN1 | Fibrillin-1 | 2871 |  | 39 | 91 | 133 | 34 |  | 13.1 | 30.6 | 44.8 | 11.4 |  | 1.3566 | 1.9595 | 1.3032 |  | 5.402E-07 | 1.311E-15 | 1.396E-02 |
| 815 | O15230 | LAMA5 | Laminin subunit alpha-5 | 3695 |  | 20 | 36 | 31 | 70 |  | 12.7 | 22.9 | 19.7 | 44.6 |  | 0.9673 | 0.8175 | 3.2473 |  | 1.984E-02 | 7.308E-02 | 1.316E-18 |
| 816 | Q09666 | AHNAK | Neuroblast differentiation-associated protein AHNAK | 5890 |  | 38 | 72 | 84 | 107 |  | 12.6 | 23.9 | 27.9 | 35.5 |  | 1.0590 | 1.3373 | 2.9707 |  | 3.055E-04 | 2.337E-06 | 1.520E-24 |
| 817 | P11166 | SLC2A1 | Solute carrier family 2, facilitated glucose transporter member 1 | 492 |  | 1 | 0 | 0 | 7 |  | 12.5 | 0.0 | 0.0 | 87.5 |  | -0.6918 | -0.6334 | 3.3667 |  | 9.666E-01 | 9.567E-01 | 4.139E-03 |
| 818 | P26447 | S100A4 | Protein S100-A4 | 101 |  | 5 | 6 | 12 | 18 |  | 12.2 | 14.6 | 29.3 | 43.9 |  | 0.3705 | 1.2993 | 3.1168 |  | 8.897E-01 | 9.046E-02 | 1.484E-05 |
| 819 | O00468 | AGRN | Agrin | 2045 |  | 1 | 2 | 2 | 4 |  | 11.1 | 22.2 | 22.2 | 44.4 |  | 0.6869 | 0.7453 | 2.7141 |  | 9.423E-01 | 9.251E-01 | 8.270E-02 |
| 820 | P31146 | CORO1A | Coronin-1A | 461 |  | 4 | 7 | 17 | 8 |  | 11.1 | 19.4 | 47.2 | 22.2 |  | 0.8086 | 2.0131 | 2.3094 |  | 4.574E-01 | 3.257E-03 | 3.087E-02 |
| 821 | Q99439 | CNN2 | Calponin-2 | 309 |  | 5 | 19 | 21 | 0 |  | 11.1 | 42.2 | 46.7 | 0.0 |  | 1.8533 | 2.0477 | -0.8311 |  | 3.422E-03 | 9.500E-04 | 2.422E-01 |
| 822 | Q01082 | SPTBN1 | Spectrin beta chain, brain 1 | 2364 |  | 20 | 45 | 39 | 78 |  | 11.0 | 24.7 | 21.4 | 42.9 |  | 1.2801 | 1.1377 | 3.4022 |  | 8.132E-04 | 5.471E-03 | 9.456E-22 |
| 823 | Q14767 | LTBP2 | Latent-transforming growth factor beta-binding protein 2 | 1821 |  | 5 | 21 | 20 | 0 |  | 10.9 | 45.7 | 43.5 | 0.0 |  | 1.9893 | 1.9813 | -0.8311 |  | 1.182E-03 | 1.644E-03 | 2.422E-01 |
| 824 | P45880 | VDAC2 | Voltage-dependent anion-selective channel protein 2 | 294 |  | 2 | 8 | 9 | 0 |  | 10.5 | 42.1 | 47.4 | 0.0 |  | 1.6657 | 1.8723 | 0.1125 |  | 8.061E-02 | 4.182E-02 | 8.264E-01 |
| 825 | P11047 | LAMC1 | Laminin subunit gamma-1 | 1609 |  | 5 | 10 | 0 | 33 |  | 10.4 | 20.8 | 0.0 | 68.8 |  | 1.0047 | -2.1076 | 3.9506 |  | 2.300E-01 | 7.092E-02 | 7.923E-12 |
| 826 | P09960 | LTA4H | Leukotriene A-4 hydrolase | 611 |  | 5 | 11 | 31 | 2 |  | 10.2 | 22.4 | 63.3 | 4.1 |  | 1.1276 | 2.5839 | 0.5478 |  | 1.536E-01 | 2.782E-06 | 8.382E-01 |
| 827 | Q6YN16 | HSDL2 | Hydroxysteroid dehydrogenase-like protein 2 | 418 |  | 1 | 4 | 5 | 0 |  | 10.0 | 40.0 | 50.0 | 0.0 |  | 1.3789 | 1.6889 | 0.6431 |  | 3.176E-01 | 1.666E-01 | 7.108E-01 |
| 828 | P02042 | HBD | Hemoglobin subunit delta | 147 |  | 102 | 147 | 202 | 640 |  | 9.3 | 13.5 | 18.5 | 58.7 |  | 0.6819 | 1.1998 | 4.2327 |  | 5.339E-04 | 2.232E-11 | 0.000E+00 |
| 829 | P55268 | LAMB2 | Laminin subunit beta-2 | 1798 |  | 17 | 44 | 30 | 94 |  | 9.2 | 23.8 | 16.2 | 50.8 |  | 1.4682 | 0.9917 | 3.8900 |  | 2.096E-04 | 3.305E-02 | 4.476E-30 |
| 830 | P29373 | CRABP2 | Cellular retinoic acid-binding protein 2 | 138 |  | 1 | 10 | 0 | 0 |  | 9.1 | 90.9 | 0.0 | 0.0 |  | 2.4788 | -0.6334 | 0.6431 |  | 7.387E-03 | 9.567E-01 | 7.108E-01 |
| 831 | Q8IWL1 | SFTPA2 | Pulmonary surfactant-associated protein A2 | 248 |  | 7 | 35 | 16 | 22 |  | 8.8 | 43.8 | 20.0 | 27.5 |  | 2.2937 | 1.2795 | 2.9892 |  | 4.227E-06 | 5.189E-02 | 3.435E-06 |
| 832 | P55083 | MFAP4 | Microfibril-associated glycoprotein 4 | 255 |  | 14 | 58 | 62 | 30 |  | 8.5 | 35.4 | 37.8 | 18.3 |  | 2.1174 | 2.2704 | 2.5304 |  | 2.274E-08 | 1.407E-09 | 2.049E-06 |
| 833 | P00325 | ADH1B | Alcohol dehydrogenase 1B | 375 |  | 12 | 23 | 36 | 71 |  | 8.5 | 16.2 | 25.4 | 50.0 |  | 1.0291 | 1.7077 | 3.9495 |  | 5.132E-02 | 1.699E-04 | 1.622E-23 |
| 834 | P12724 | RNASE3 | Eosinophil cationic protein | 160 |  | 1 | 4 | 4 | 3 |  | 8.3 | 33.3 | 33.3 | 25.0 |  | 1.3789 | 1.4373 | 2.4091 |  | 3.176E-01 | 3.044E-01 | 2.127E-01 |
| 835 | P01024 | C3 | Complement C3 | 1663 |  | 4 | 2 | 17 | 36 |  | 6.8 | 3.4 | 28.8 | 61.0 |  | -0.5357 | 2.0131 | 4.3239 |  | 7.588E-01 | 3.257E-03 | 6.776E-14 |
| 836 | Q99714 | HSD17B10 | 3-hydroxyacyl-CoA dehydrogenase type-2 | 261 |  | 1 | 9 | 5 | 0 |  | 6.7 | 60.0 | 33.3 | 0.0 |  | 2.3445 | 1.6889 | 0.6431 |  | 1.420E-02 | 1.666E-01 | 7.108E-01 |
| 837 | Q16787 | LAMA3 | Laminin subunit alpha-3 | 3333 |  | 2 | 12 | 0 | 19 |  | 6.1 | 36.4 | 0.0 | 57.6 |  | 2.1845 | -1.1640 | 4.1337 |  | 7.930E-03 | 5.166E-01 | 8.504E-08 |
| 838 | Q6NZI2 | PTRF | Polymerase I and transcript release factor | 390 |  | 7 | 19 | 18 | 82 |  | 5.6 | 15.1 | 14.3 | 65.1 |  | 1.4526 | 1.4379 | 4.8397 |  | 1.628E-02 | 2.162E-02 | 2.638E-32 |
| 839 | P05997 | COL5A2 | Collagen alpha-2(V) chain | 1499 |  | 1 | 5 | 5 | 9 |  | 5.0 | 25.0 | 25.0 | 45.0 |  | 1.6305 | 1.6889 | 3.6802 |  | 1.760E-01 | 1.666E-01 | 5.212E-04 |
| 840 | Q9NZN4 | EHD2 | EH domain-containing protein 2 | 543 |  | 3 | 15 | 11 | 59 |  | 3.4 | 17.0 | 12.5 | 67.0 |  | 2.0920 | 1.7425 | 5.3264 |  | 4.249E-03 | 3.460E-02 | 1.625E-25 |
|  |  |  |  |  |  | 23891 | 21438 | 20578 | 8498 |  |  |  |  |  |  |  |  |  |  |  |  |  |

Table S4: The STRING network enrichment results on KEGG pathways.

| GO_id | Term | Number of Genes | *p*-value_fdr |
| --- | --- | --- | --- |
|  |  |  |  |
| A. LPIA |  |  |  |
|  |  |  |  |
| 3010 | Ribosome | 19 | 2.58E-18 |
| 970 | Aminoacyl-tRNA biosynthesis | 9 | 1.06E-09 |
| 5100 | Bacterial invasion of epithelial cells | 8 | 2.33E-06 |
| 1100 | Metabolic pathways | 25 | 7.74E-06 |
| 5200 | Pathways in cancer | 13 | 1.00E-05 |
| 4520 | Adherens junction | 7 | 1.71E-05 |
| 5130 | Pathogenic Escherichia coli infection | 6 | 4.40E-05 |
| 5205 | Proteoglycans in cancer | 10 | 5.94E-05 |
| 640 | Propanoate metabolism | 5 | 6.01E-05 |
| 4062 | Chemokine signaling pathway | 9 | 7.23E-05 |
| 3050 | Proteasome | 5 | 2.11E-04 |
| 4670 | Leukocyte transendothelial migration | 7 | 2.11E-04 |
| 280 | Valine, leucine and isoleucine degradation | 5 | 2.11E-04 |
| 5213 | Endometrial cancer | 5 | 3.70E-04 |
| 4144 | Endocytosis | 8 | 7.17E-04 |
| 52 | Galactose metabolism | 4 | 7.36E-04 |
| 4015 | Rap1 signaling pathway | 8 | 9.72E-04 |
| 4917 | Prolactin signaling pathway | 5 | 1.38E-03 |
| 4530 | Tight junction | 6 | 2.77E-03 |
| 4012 | ErbB signaling pathway | 5 | 2.99E-03 |
| 520 | Amino sugar and nucleotide sugar metabolism | 4 | 3.06E-03 |
| 531 | Glycosaminoglycan degradation | 3 | 3.06E-03 |
| 4120 | Ubiquitin mediated proteolysis | 6 | 3.06E-03 |
| 5206 | MicroRNAs in cancer | 6 | 4.07E-03 |
| 4915 | Estrogen signaling pathway | 5 | 4.07E-03 |
| 4660 | T cell receptor signaling pathway | 5 | 4.98E-03 |
| 4630 | Jak-STAT signaling pathway | 6 | 5.48E-03 |
| 4066 | HIF-1 signaling pathway | 5 | 5.77E-03 |
| 4014 | Ras signaling pathway | 7 | 5.77E-03 |
| 5212 | Pancreatic cancer | 4 | 6.19E-03 |
| 5214 | Glioma | 4 | 6.19E-03 |
| 410 | beta-Alanine metabolism | 3 | 7.78E-03 |
| 4722 | Neurotrophin signaling pathway | 5 | 8.12E-03 |
| 1230 | Biosynthesis of amino acids | 4 | 9.48E-03 |
| 4110 | Cell cycle | 5 | 9.54E-03 |
| 5203 | Viral carcinogenesis | 6 | 9.54E-03 |
| 5166 | HTLV-I infection | 7 | 9.79E-03 |
| 5412 | Arrhythmogenic right ventricular cardiomyopathy (ARVC) | 4 | 9.92E-03 |
| 5160 | Hepatitis C | 5 | 1.11E-02 |
| 4914 | Progesterone-mediated oocyte maturation | 4 | 1.26E-02 |
| 4510 | Focal adhesion | 6 | 1.44E-02 |
| 4540 | Gap junction | 4 | 1.44E-02 |
| 5215 | Prostate cancer | 4 | 1.54E-02 |
| 4141 | Protein processing in endoplasmic reticulum | 5 | 2.69E-02 |
| 500 | Starch and sucrose metabolism | 3 | 2.69E-02 |
| 5110 | Vibrio cholerae infection | 3 | 2.78E-02 |
| 5223 | Non-small cell lung cancer | 3 | 3.19E-02 |
| 5221 | Acute myeloid leukemia | 3 | 3.29E-02 |
| 330 | Arginine and proline metabolism | 3 | 3.54E-02 |
| 5145 | Toxoplasmosis | 4 | 3.54E-02 |
| 4919 | Thyroid hormone signaling pathway | 4 | 3.81E-02 |
| 10 | Glycolysis / Gluconeogenesis | 3 | 3.87E-02 |
| 4142 | Lysosome | 4 | 4.25E-02 |
| 5120 | Epithelial cell signaling in Helicobacter pylori infection | 3 | 4.64E-02 |
| 4320 | Dorso-ventral axis formation | 2 | 4.64E-02 |
|  |  |  |  |
| B. MIA |  |  |  |
|  |  |  |  |
| 5205 | Proteoglycans in cancer | 17 | 3.99E-17 |
| 500 | Starch and sucrose metabolism | 6 | 1.51E-06 |
| 4151 | PI3K-Akt signaling pathway | 10 | 8.25E-06 |
| 4512 | ECM-receptor interaction | 6 | 1.91E-05 |
| 4015 | Rap1 signaling pathway | 7 | 1.95E-04 |
| 4810 | Regulation of actin cytoskeleton | 6 | 1.93E-03 |
| 5200 | Pathways in cancer | 7 | 1.93E-03 |
| 4014 | Ras signaling pathway | 6 | 1.93E-03 |
| 5218 | Melanoma | 4 | 1.93E-03 |
| 4514 | Cell adhesion molecules (CAMs) | 5 | 1.93E-03 |
| 4510 | Focal adhesion | 5 | 1.11E-02 |
| 1100 | Metabolic pathways | 11 | 2.08E-02 |
| 4145 | Phagosome | 4 | 2.26E-02 |
| 5133 | Pertussis | 3 | 2.71E-02 |
| 5412 | Arrhythmogenic right ventricular cardiomyopathy (ARVC) | 3 | 2.97E-02 |
| 5410 | Hypertrophic cardiomyopathy (HCM) | 3 | 3.89E-02 |
| 534 | Glycosaminoglycan biosynthesis - heparan sulfate / heparin | 2 | 4.23E-02 |
| 5414 | Dilated cardiomyopathy | 3 | 4.23E-02 |
|  |  |  |  |
|  |  |  |  |
| C. AIS |  |  |  |
|  |  |  |  |
| 4510 | Focal adhesion | 17 | 2.69E-16 |
| 5100 | Bacterial invasion of epithelial cells | 11 | 3.72E-13 |
| 4670 | Leukocyte transendothelial migration | 12 | 9.79E-13 |
| 4520 | Adherens junction | 10 | 6.45E-12 |
| 4810 | Regulation of actin cytoskeleton | 11 | 1.70E-08 |
| 4530 | Tight junction | 9 | 4.94E-08 |
| 5412 | Arrhythmogenic right ventricular cardiomyopathy (ARVC) | 6 | 1.23E-05 |
| 4360 | Axon guidance | 7 | 1.33E-05 |
| 5213 | Endometrial cancer | 5 | 3.67E-05 |
| 650 | Butanoate metabolism | 4 | 7.09E-05 |
| 5203 | Viral carcinogenesis | 7 | 1.29E-04 |
| 5205 | Proteoglycans in cancer | 7 | 3.97E-04 |
| 280 | Valine, leucine and isoleucine degradation | 4 | 4.71E-04 |
| 5130 | Pathogenic Escherichia coli infection | 4 | 8.18E-04 |
| 5146 | Amoebiasis | 5 | 8.18E-04 |
| 4062 | Chemokine signaling pathway | 6 | 9.44E-04 |
| 4611 | Platelet activation | 5 | 1.78E-03 |
| 4015 | Rap1 signaling pathway | 6 | 1.78E-03 |
| 640 | Propanoate metabolism | 3 | 3.42E-03 |
| 380 | Tryptophan metabolism | 3 | 5.87E-03 |
| 72 | Synthesis and degradation of ketone bodies | 2 | 6.59E-03 |
| 71 | Fatty acid degradation | 3 | 7.13E-03 |
| 1212 | Fatty acid metabolism | 3 | 9.43E-03 |
| 310 | Lysine degradation | 3 | 1.02E-02 |
| 5200 | Pathways in cancer | 6 | 1.38E-02 |
| 5131 | Shigellosis | 3 | 1.38E-02 |
| 4370 | VEGF signaling pathway | 3 | 1.47E-02 |
| 4612 | Antigen processing and presentation | 3 | 1.92E-02 |
| 4145 | Phagosome | 4 | 1.92E-02 |
| 3320 | PPAR signaling pathway | 3 | 1.94E-02 |
| 4390 | Hippo signaling pathway | 4 | 1.94E-02 |
| 4917 | Prolactin signaling pathway | 3 | 1.94E-02 |
| 5166 | HTLV-I infection | 5 | 2.08E-02 |
| 900 | Terpenoid backbone biosynthesis | 2 | 2.31E-02 |
| 4146 | Peroxisome | 3 | 2.52E-02 |
| 4012 | ErbB signaling pathway | 3 | 2.88E-02 |
| 5152 | Tuberculosis | 4 | 2.88E-02 |
| 5216 | Thyroid cancer | 2 | 3.40E-02 |
| 410 | beta-Alanine metabolism | 2 | 3.72E-02 |
| 5322 | Systemic lupus erythematosus | 3 | 3.72E-02 |
| 1200 | Carbon metabolism | 3 | 4.55E-02 |
|  |  |  |  |
|  |  |  |  |
