# Supplementary figures and images for: A proteomic profiling of laser-microdissected lung adenocarcinoma cells of early lepidic-types
Source: Clin Transl Med. 2015 Jul 3;4:24. doi: 10.1186/s40169-015-0064-3 (PMC4501340; doi:10.1186/s40169-015-0064-3)

## Slide 1
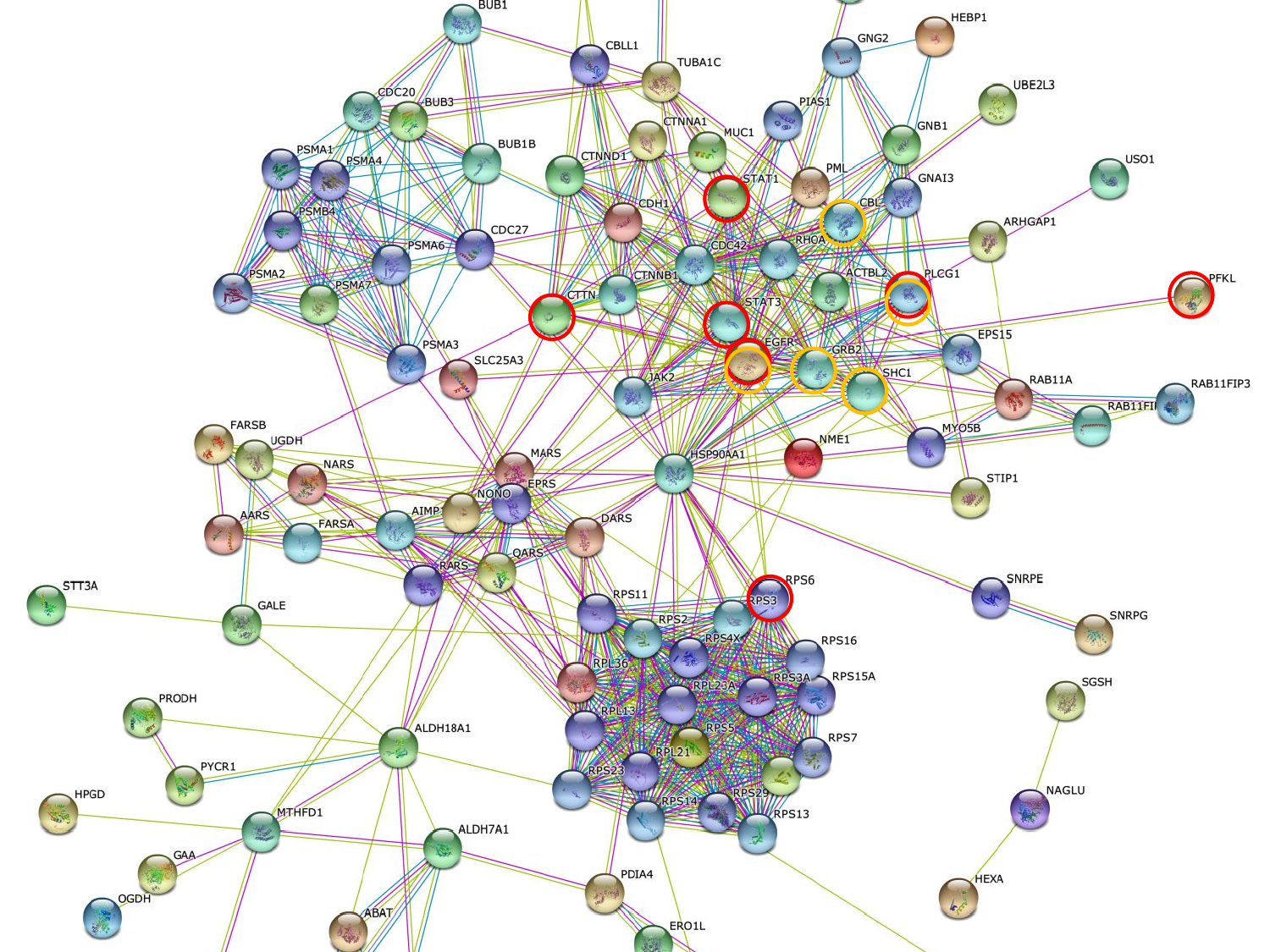

Supplement: Additional file 2: Figure S1. — The high-resolution evidence-view of STRING PPI networks obtained on LPIA by using 70 proteins significantly expressed (listed in Table 1), which were generated using default setting in network depth of 50 interactions under medium confidence (0.4) and standard criteria for linkage only with experiments, databases, and text mining. [file 40169_2015_64_MOESM2_ESM.ppt]

## Slide 1
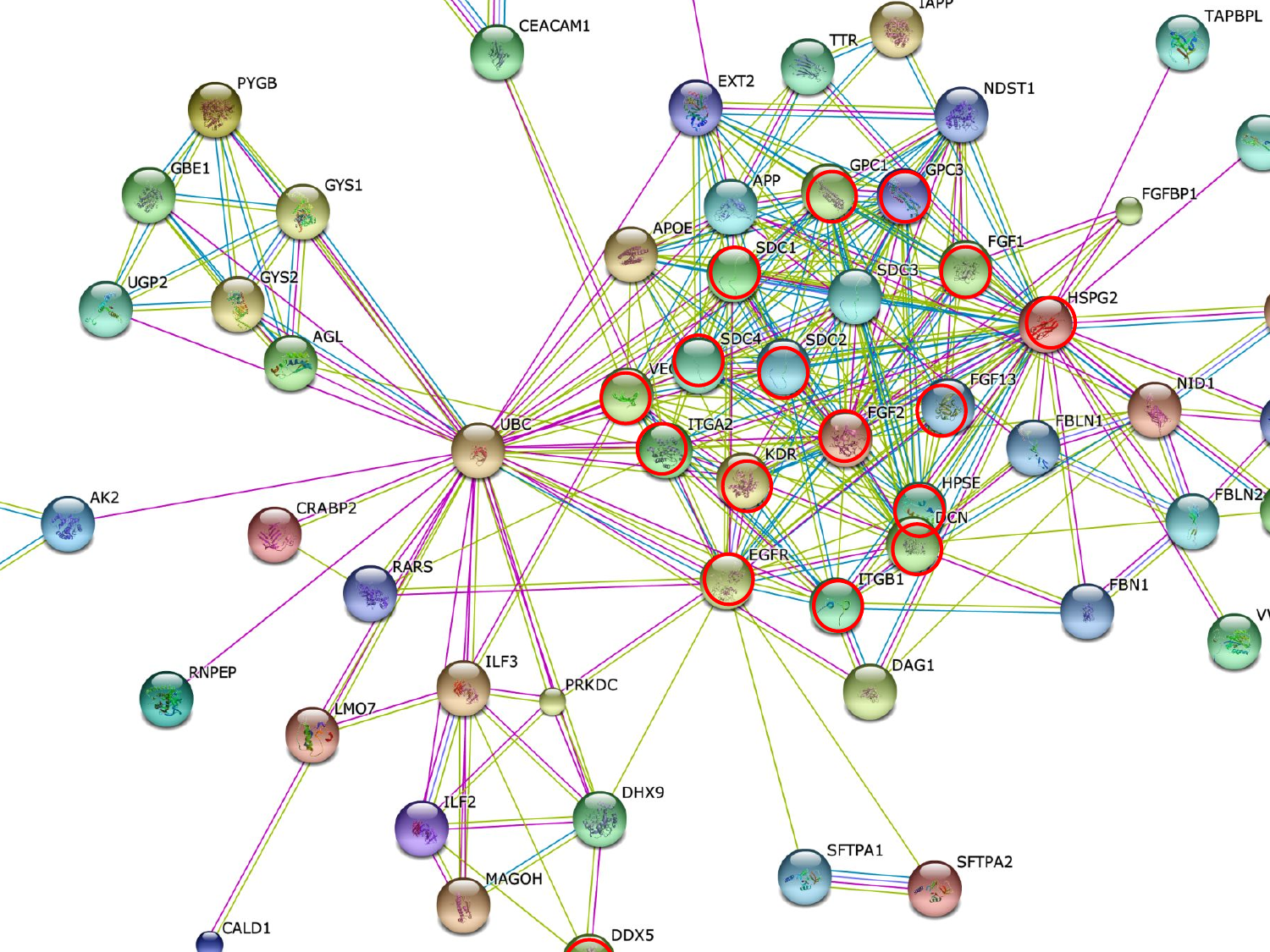

Supplement: Additional file 3: Figure S2. — The high-resolution evidence-view of STRING PPI networks obtained on AIS by using 26 proteins significantly expressed (listed in Additional file 1: Table S1), which were generated using default setting in network depth of 50 interactions under medium confidence (0.4) and standard criteria for linkage only with experiments, databases, and text mining. [file 40169_2015_64_MOESM3_ESM.ppt]

## Slide 1
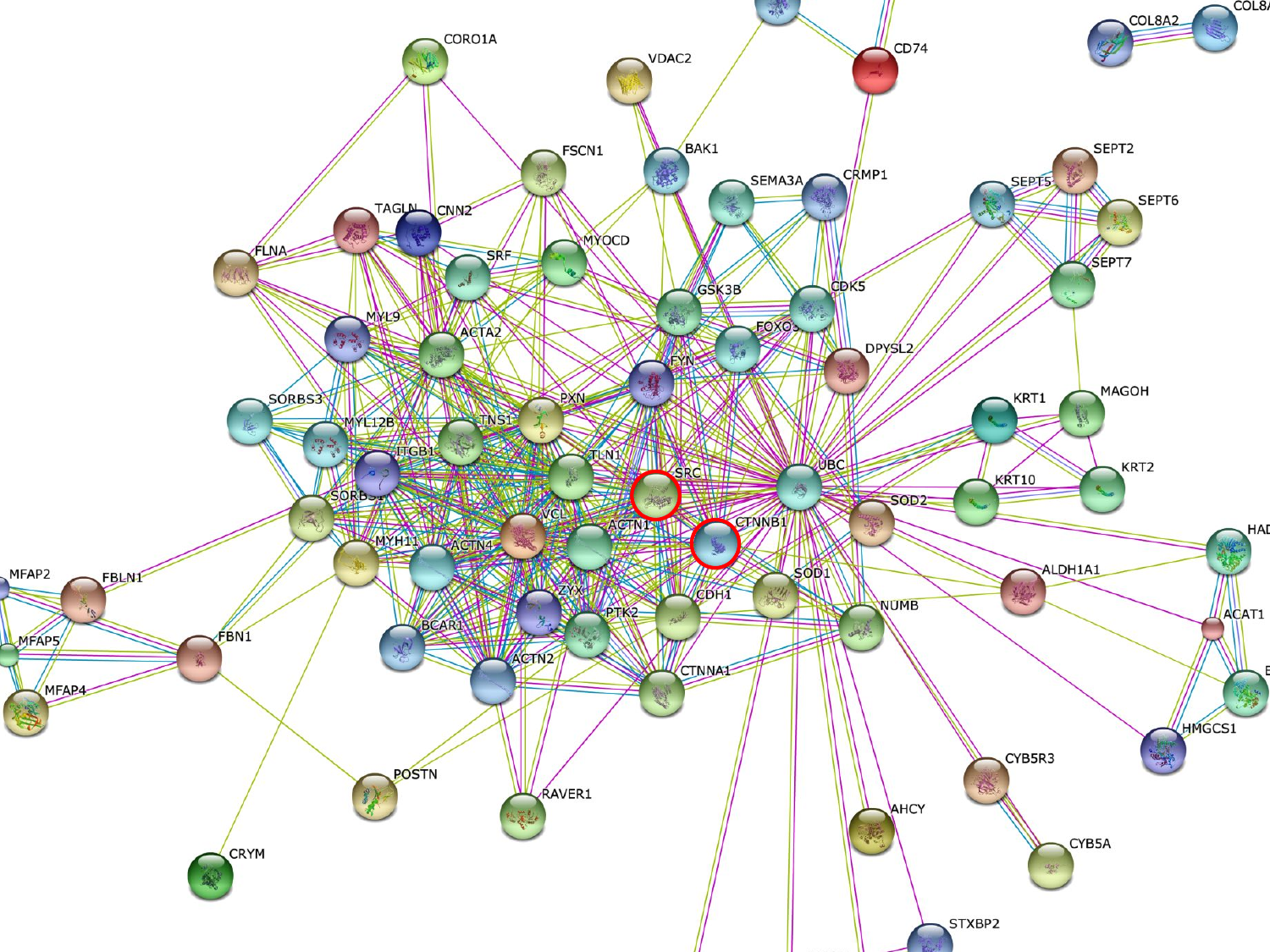

Supplement: Additional file 4: Figure S3. — The high-resolution evidence-view of STRING PPI networks obtained on MIA by using 15 proteins significantly expressed (listed in Additional file 1: Table S2), which were generated using default setting in network depth of 50 interactions under medium confidence (0.4) and standard criteria for linkage only with experiments, databases, and text mining. [file 40169_2015_64_MOESM4_ESM.ppt]
